# Supplementary material for: The Spectrum of Disease-Associated Alleles in Countries with a Predominantly Slavic Population
Source: Int J Mol Sci. 2024 Aug 28;25(17):9335. doi: 10.3390/ijms25179335 (PMC11394759; doi:10.3390/ijms25179335)
Supplement: Supplementary file 1 [file ijms-25-09335-s001.zip › ijms-3137350-supplementary.pdf]

Electronic supplementary files

Supplementary Table S1 – Recurrent pathogenic alleles of presumably Slavic origin

Supplementary Table S2 – Spectrum and share of recurrent pathogenic alleles characteristic for Slavs and four well-known founder populations (Jewish, Icelandic, Finnish, French Canadians)

Supplementary Table S1. Recurrent pathogenic alleles of presumably Slavic origin

Gene, RefSeq accession number, variant name (HGVS, other names), dbSNP identifier, ClinVar accession number	Disease, OMIM disease ID, type of inheritance, prevalence (Orphanet)	Prevalence of allele in patients from Slavic countries (in subjects with clinical diagnosis of genetic disease, or in carriers of all pathogenic alleles of the involved gene)	Prevalence of allele in non-Slavic populations	MAF in Slavic populations (literature data and Ruseq database)**	Highest MAF (gnomAD v2.1.1)	Haplotype	Comments
**AAAS**<br>NM_015665.6<br><br>c.787T>C<br>(p.Ser263Pro)<br><br>rs121918550<br>VCV000005045.3<br><br>Pathogenic / Likely pathogenic	Allgrove syndrome (triple A syndrome)<br><br>#231550<br><br>AR<br><br><1:1000000	Croatia: 4/10 (40%) [Dumic et al., 2012]	This mutation is occasionally found in non-Slavic countries [Milenković et al., 2008]	Russia: 5/10930 (0.0005)	Finns: 18/25078 (0.00072)<br>NFE: 16/127710 (0.00013)<br>Estonians: 3/4818 (0.00062)<br>Bulgarians: 1/2654 (0.00038)	At least 66% alleles share the same haplotype [Milenković et al., 2008]	Central European (?) or Slavic (?) mutation
**AAAS**<br>NM_015665.6<br><br>c.1159C>T<br>(p.Gln387*)<br><br>rs763820204<br>VCV000264990.5<br><br>Pathogenic		Croatia: 3/10 (30%) [Dumic et al., 2012]	This mutation was not reported outside Slavic countries				Regional (Croatian) mutation
**AAAS**<br>NM_015665.6<br><br>c.43C>A<br>(p.Gln15Lys)<br><br>Also reported as p.Gly14Valfs*45		Croatia: 2/10 (20%) [Dumic et al., 2012]<br>Russia: 1 homozygous proband [Volkova et al., 2020]	This mutation is found in various European populations and also in India, but appears to be more frequent in Slavic populations	Russia: 1/10908 (0.0001)	SAS: 17/30616 (0.00055)<br>NFE: 26/129106 (0.0002)<br>Bulgarians: 2/2664 (0.00075)		Slavic mutation (?)

reflecting the impact of this substitution on splicing<br><br>rs121918549<br>VCV000005044.13<br><br>Pathogenic							
**ADSL**<br>NM_000026.4<br><br>c.1277G>A (p.Arg426His)<br><br>rs119450941<br>VCV000002462.5<br><br>Pathogenic	Adenylosuccinase deficiency<br><br>#103050<br><br>AR<br><br><1:1000000	Poland: 6/12 (50%) [Jurecka et al., 2008]<br>Czech Republic: 3/10 (30%) [Kmoch et al., 2000]	The mutation is considered to be the most frequent (in Europe), but the majority of reported patients are from Germany, Poland and Czech Republic		NFE: 42/129196 (0.00033)<br>Swedish: 19/26134 (0.00073)<br>Bulgarians: 0/2670		Pan-European mutation, with particularly high frequency in Poland
**ADPRS**<br>(also known as ADPRHL2)<br>NM_017825.3<br><br>c.1004T>G (p.Val335Gly)<br><br>rs201735454<br>VCV000599343.30<br><br>Pathogenic / Uncertain significance	Stress-induced childhood-onset neurodegeneration with variable ataxia and seizures<br><br>#618170<br><br>AR	Poland, Cosovan Serbs: 4/4 (100%) [Danhauser et al., 2018]		Russia: 4/8126 (0.0005)	NFE: 26/129188 (0.0002)<br>Bulgarians: 2/2670 (0.00075)		Slavic mutation
**AIRE**<br>NM_000383.4<br><br>c.769C>T (p.Arg257*)<br><br>rs121434254<br>VCV000003307.12	Autoimmune polyendocrinopathy –candidiasis– ectodermal dystrophy (APECED)<br><br>#240300	Slovenia: 14/20 (70%) alleles [Podkrajsek et al., 2005]<br>Poland: 20/28 (71%) [Stolarski et al., 2006]<br>Serbia: 22/24 (92%) [Fierabracci et al., 2021]<br>Russia: 64/92 (70%) [Orlova et al., 2010]	Major Finnish founder mutation, also occurs in other populations of European descent	Poland: 8/4016 (0.002) [Stolarski et al., 2006]<br>Russia: 33/10920 (0.003)	Finns: 125/25064 (0.005)<br>NFE: 84/126976 (0.00066)<br>Estonians: 14/4806 (0.00291)<br>Bulgarians: 3/2592 (0.00116)	The most (but not all) carriers share the same haplotype [Wang et al., 1998]	Finnish (?) / Balto-Slavic mutation

Pathogenic	AR<br><br>1:1000000 – 9:1000000<br>1:25000 in Finland						
**ALMS1**<br>NM_015120.4<br><br>c.11880_11881delTT (p.Ser3961Leufs)<br><br>Also reported as c.11881_11882delTT (p.Val3959Leufs)<br><br>rs758732551<br>Not reported in ClinVar<br><br>Uncertain significance / Pathogenic	Alström syndrome<br><br>#203800<br><br>AR<br><br>1:1000000 - 9:1000000	Poland: 5/16 (31%) [Zmyslowska et al., 2016]	Not reported outside Poland		NFE: 1/113454 (<0.00001)<br>Bulgarians: 1/2666 (0.00038)		Regional (Polish ?) mutation
**ALOXE3**<br>NM_001165960.1<br><br>c.1096C>T (p.Arg366*)<br><br>Also reported as c.700C>T (p. Arg234*)<br><br>rs121434233<br>VCV000003408.28<br><br>Pathogenic	Autosomal recessive congenital ichthyosis-3 (lamellar ichthyosis; self-healing collodion baby)<br><br>#606545<br><br>AR<br><br>1:1000000 - 9:1000000	Czech Republic: 9/18 (50%) [Borská et al., 2019]; 6/12 (50%) [Bučková et al., 2016]	Major recurrent mutaton worldwide, e.g., 11/31 (35%) in a large international study [Eckl et al., 2009]	Russia: 31/10830 (0.0029)	OTH: 3/7228 (0.00042)<br>NFE: 38/129192 (0.00029)<br>Bulgarians: 6/2670 (0.00225)	At least some carriers of this recurrent allele do not share the same haplotype [Eckl et al., 2009]	Slavic founder mutation (?)<br>Hotspot mutation (?)
**AP4B1**<br>NM_001253852.3	Hereditary spastic paraplegia (SPG47)	Poland: 8/8 (100%) (4 homozygous probands)	Reported once in an individual of mixed	Russia: 18/10728	ASJ: 7/10358 (0.00068)	All carriers share the same	Slavic mutation

c.1160_1161del (p.Thr387Argfs)<br><br>rs587779388 VCV000156414.59<br><br>Pathogenic / Likely pathogenic	#614066<br><br>AR<br><br><1:1000000	[Szczałuba et al., 2020]<br>Czech Republic: 1 homozygous case [Abdollahpour et al., 2015]	ethnicity (ASJ/EAS/AFR) [Ebrahimi-Fakhari et al., 2017]	(0.0017)	NFE: 29/128882 (0.00023)<br>Bulgarians: 4/2666 (0.0015)	haplotype [Szczałuba et al., 2020]	
**ARSB** NM_000046.5<br><br>c.454C>T (p.Arg152Trp)<br><br>rs991104525 VCV000496789.11<br><br>Pathogenic	Mucopolysaccharidosis VI (Maroteaux-Lamy)<br><br>#253200<br><br>AR<br><br>1:43261 – 1:1505160	Lithuania: 4/6 (67%)<br>Estonia: 3/4 (75%)<br>Poland: 8/14 (57%)<br>Belarus: 6/16 (38%)<br>Russia: 15/32 (47%) [Jurecka et al., 2012; Jurecka et al., 2014]	The most frequent mutation, which was occasionally identified in Western Europe and Turkey, but is much more frequent in Eastern Europe [Tomanin et al., 2018]	Russia: 2/10916 (0.0002)	OTH: 2/7228 (0.00027)<br>NFE: 12/129186 (0.00009)<br>Estonians: 8/4832 (0.00166)<br>Bulgarians: 0		Balto-Slavic mutation
**ARSB** NM_000046.5<br><br>c.797A>C (p.Tyr266Ser)<br><br>rs1554086402 VCV000559814.6<br><br>Pathogenic / Likely pathogenic / Uncertain significance		Belarus: 6/16 (38%) [Jurecka et al., 2012; Jurecka et al., 2014]	Only one case was reported outside Belarus [Karageorgos et al., 2007]	Russia: 0/1600			Regional (Belarusian) mutation
**ARSB** NM_000046.5<br><br>c.1562G>A (p.Cys521Tyr)<br><br>rs1554069661 VCV000559723.1		Russia: 6/32 (19%) [Jurecka et al., 2012; Jurecka et al., 2014]	Only one case was reported outside Russia [Isbrandt et al., 1994]	Russia: 0/1600			Regional (Russian) mutation

Uncertain significance / Likely pathogenic							
**ATM**<br>NM_000051.3<br><br>c.5932G>T (p.Glu1978Ter)<br><br>rs587779852<br>VCV000127414.9<br><br>Pathogenic	Ataxia-telangiectasia<br><br>#208900<br><br>AR<br><br>1:1000000 - 9:1000000<br><br>Low penetrance breast cancer predisposition<br><br>#114480	Poland (ataxia-telangiectasia (AT) patients): 6/38 (16%) [Podralska et al., 2014]; 5/44 (11%) [Mitui et al., 2005]<br>Russia: 13/30 (43%) AT patients [Suspitsin et al., 2019]	Occasionally occurs in other Caucasian populations (ClinVar) It is also common in Mennonites, a religious community, which originated in Germany, was driven to East Europe and Russia by religious persecution and finally emigrated to North America	Poland: 1/4020 (0.0002)<br>Belarus: 1/2038 (0.0005) [Bogdanova et al., 2009]<br>Russia: 8/10672 (0.0007)	OTH: 1/6114 (0.00016)<br>NFE: 10/113596 (0.00009)<br>Bulgarians: 1/2670 (0.00037)	Russian and Polish carriers share the same haplotype [Mitui et al., 2005]	Slavic mutation
**ATM**<br>NM_000051.3<br><br>c.5971G>T (p.Glu1991Ter)<br><br>Not reported in dbSNP<br>Not reported in ClinVar<br><br>Uncertain significance / Likely pathogenic		Poland (ATM heterozygous breast cancer patients): 2/6 (33%) [Cybulski et al., 2019]	Extremely rare outside Poland (Clinvar)		NFE: 1/113554 (<0.00001)<br>North-Western Europeans: 1/42120 (0.00002)<br>Bulgarians: 0/2666		Ultra-rare mutation, found twice in Poland
**ATM**<br>NM_000051.3<br><br>c.7630-2A>C<br><br>rs587779866<br>VCV000127447.12<br><br>Pathogenic		Poland (AT patients): 6/38 (16%) [Podralska et al., 2014]; 7/44 (16%) [Mitui et al., 2005]; 4/44 (9%) [Telatar et al., 1998]<br>Poland (breast cancer patients): 2/6 (33%) [Cybulski et al., 2019]	Occasionally reported in other Caucasian populations (ClinVar)	Russia: 1/10784 (0.0001)	NFE: 3/128914 (0.00002)<br>Bulgarians: 0/2668		Regional (Polish) mutation
**ATP7B**<br>NM_000053.4	Wilson disease	Slovakia: 28/50 (56%) [Poláková et al., 2007]	Pan-European mutation, the most prevalent in Europe	Russia: 11/1794	ASJ: 55/10356 (0.00531)	All carriers share the same	Pan-European mutation of Balto-

c.3207C>A (p.His1069Gln)<br><br>rs76151636<br>VCV000003848.19<br><br>Pathogenic	#277900<br><br>AR<br><br>1:100000 - 9:100000	Croatia: 67/125 (54%) [Ljubić et al., 2016]<br>Bulgaria: 94/152 (62%) [Todorov et al., 2005]<br>Czech Republic: 228/321 (71%) [Vrabelova et al., 2005]<br>Poland: 327/496 (66%) [Kluska et al., 2019]	and USA. Still, it shows clear East to West and North to South gradient, peaking in frequency in Baltic countries and Poland, and declining to virtual absence in Spain; very common in Ashkenazi Jews [Gomes and Dedoussis, 2016]	(0.0061) [Yanus et al., 2019]	NFE: 196/128580 (0.00152)<br>Estonians: 44/4824 (0.00912)<br>Bulgarians: 7/2658 (0.00263)	haplotype<br>haplotype [Firneisz et al., 2002]	Slavic origin
**ATRIP**<br>NM_130384.3<br><br>c.1152_1155del (p.Thr384_Gly385insTer)<br><br>rs753154444<br>VCV002416474.3<br><br>Uncertain significance (relevance of the gene to the diasea phenotype is not firmly established)	Low penetrance breast cancer predisposition [Cybulski et al., 2023]<br><br>Not reported in OMIM<br><br>AD?	Poland: 2/3 (66%) heterozygous ATRIP mutation carriers [Cybulski et al., 2023]		Russia: 7/10114 (0.0007)	NFE: 14/129008 (0.0001)<br>Estonians: 5/4828 (0.001)<br>Bulgarians: 1/2670 (0.00037)		Slavic mutation
**ATXN1**<br>NM_000332.3<br><br>c.589_591CAGins (p.Gln208_His209ins)<br><br>Also reported as ATXN1 (CAG)n repeat expansion<br><br>rs193922926<br>VCV000008071.2<br><br>Pathogenic	Spinocerebellar ataxia 1<br><br>#164400<br><br>AD<br><br>1:100000 - 2:100000	Poland: SCA1: n = 120 (120/161, 75%); SCA2: n = 23; SCA8: n = 14; SCA17: n = 3; SCA3: n = 1 [Sułek-Piatkowska et al., 2010; Krysa et al., 2016]<br>Russia: 5/25 (20%) AD SCA patients (no SCA2, SCA3 patients identified) [Illarioshkin et al., 1996]; Russia has unusually high prevalence of SCA1<br>Serbia: SCA1: 13/54 (24%); SCA2: 5/54 (9%); Friedreich ataxia: 4/54 (7%) [Dragasević et al., 2006]	SCA1 is rare in the majority of world populations; it is frequent in Poland, Russia and South Africa. SCA2 is the most prevalent type in Europe; SCA3 is the most common type worldwide (predominant type in China and Japan) [Scott et al., 2020]	Poland: Central Poland, Mazowiecki e region: 1:41031; Other regions: 10-100 times lower [Krysa et al., 2016]		A common haplotype in a significant proportion of cases (at the very least, 1/3 of the cases) [Krysa et al., 2016]	Hotspot mutation, with unusually high frequency in Slavic populations due to founder effect
**BARD1**<br>NM_000465.4	Low penetrance breast cancer	Poland: 1/3 (33%) heterozygous BARD1 mutation carriers	Recurrent mutation in various populations of	Poland: 7/9414	NFE: 7/129052 (0.00005)		Regional (Western Slavic and

c.1690C>T<br>(p.Q564*)<br><br>rs587780021<br>VCV000127720.62<br><br>Pathogenic /<br>Uncertain<br>significance	predisposition<br><br>#114480<br><br>AD, low penetrance	[Ratajska et al., 2012]	European descent<br>[Susswein et al., 2016]	(0.0007)<br>Belarus:<br>0/2378<br>[Suszynska et al., 2019]<br>Russia:<br>2/10800<br>(0.0002)	Estonians: 1/4828<br>(0.0002)<br>Bulgarians: 0/2668		Belarusian?) mutation
**BEST1**<br>NM_004183.4<br><br>c.313G>C<br>(p.Arg105Gly)<br><br>c.133C>G<br>p.Arg45Gly,<br>according to<br>NM_001139443.2<br><br>Not reported in dbSNP<br>VCV000932077.3<br><br>Uncertain<br>significance / Likely<br>pathogenic	Best vitelliform<br>macular dystrophy,<br>type 2<br><br>#153700<br><br>AD<br><br>1:100000 –<br>9:100000	Slovenia: 4/5 kindreds (80%)<br>[Glavač et al., 2016]					Regional (Slovenian)<br>mutation
**BLM**<br>NM_000057.4<br><br>c.1642C>T<br>(p.Gln548*)<br><br>rs200389141<br>VCV000127478<br><br>Pathogenic	Bloom syndrome<br><br>#210900<br><br>AR<br><br>Very rare, 1:48000<br>in ASJ<br><br>Low penetrance<br>breast cancer<br>predisposition in<br>heterozygotes (?)	Poland: 3/3 (100%)<br>heterozygous BLM mutation<br>carriers [Cybulski et al., 2019]<br>Slovakia: 1 family with 3<br>homozygous cases [Vojtková et al., 2016]<br>Czech Republic: 1 family, two<br>homozygous members<br>[Trizuljak et al., 2020]		Poland:<br>26/9396<br>(0.0028)<br>[Kluźniak et al., 2019]<br>Belarus:<br>2/1235<br>(0.0008)<br>[Prokofyeva et al., 2013]<br>Russia:<br>2/2186 (0.001)<br>[Sokolenko et al., 2012];	NFE: 43/126942<br>(0.00034)<br>Estonians: 10/4826<br>(0.00207)<br>Bulgarians: 5/2650<br>(0.00189)		Slavic mutation

	AD			25/10700 (0.0023) (Ruseq)			
**BRCA1** NM_007294.4<br><br>c.5266dupC (p.Gln1756ProfsX74)<br><br>BIC nomenclature: 5382insC (p.Gln1777fs)<br><br>rs80357906 VCV000017677.19<br><br>Pathogenic	Hereditary breast and ovarian cancer (HBOC)<br><br>#604370<br><br>AD<br><br>~1:300 (?)	Russia: 68% [Hamel et al., 2011]<br>Belarus: 52% [Savanevich et al, 2021]; 41/79 (52%) [Yanus et al., 2022]<br>Poland: 204/370 (55%) [Cybulski et al., 2019]; Gdansk, ovarian cancer patients: 8/16 (80%) [Ratajska et al., 2015]; Northern Poland: 22/43; 51% [Ratajska et al., 2008]<br>Serbia: 37% [Krivokuca et al, 2021]<br>Slovenia: 9/112 (8%) [Krajc et al., 2014]<br>Macedonia: 4/22 (18%) [Jakimovska et al., 2018]<br>Bulgaria: 22/39 (56%) [Dodova et al., 2015]<br>Croatia: 1/11 (9%) [Levanat et al., 2012]<br>Czech Republic: 329/1021 (32%) [Machackova et al., 2019]; 7/14 (50%) [Mateju et al., 2010]<br>Slovakia: 5/20 (25%) of all BRCA1/BRCA2 mutations [Cierniková et al., 2006]	Major recurrent mutation in Hungary (up to 57%) and Greece (47%); relatively common in France, Germany, Austria, Denmark and Italy (7.5-18.8%), occasionally appears in other European population. One of 3 major founder HBOC mutations in Askenazi Jews [Hamel et al., 2011]	Poland: 29/33698 (0.0009) [Brozek et al., 2011]; 14/8000 (0.0018) [Górski et al., 2005]<br>Russia: 18/10922 (0.0016)	ASJ: 24/10370 (0.002314)<br>NFE: 25/129200 (0.00019)<br>Estonians: 5/4834 (0.0010)<br>Bulgarians: 2/2670 (0.00075)	All c.5266dupC carriers share the same haplotype, which has most likely originated in Northern Europe (in Russia or, possibly, Denmark) between 1800 and 1500 years ago [Hamel et al., 2011]	Central / Eastern European founder mutation of Slavic or, possibly, Danish origin; less common in Southern Slavs
**BRCA1** NM_007294.4<br><br>c.181T>G (Cys61Gly)<br><br>BIC nomenclature: 300T>G<br><br>rs28897672 VCV000017661.18<br><br>Pathogenic		Russia: one of the most common minor recurrent BRCA1 mutations [Gayther 1997; Sokolenko et al., 2020]<br>Slovenia: 28/112 (25%) [Krajc et al., 2014]<br>Ukraine: frequent mutation [Gorodetska et al., 2017]<br>Macedonia: 5/22 (23%) [Jakimovska et al., 2018];<br>Croatia: 1/11 (9%) [Levanat et al., 2012]<br>Czech Republic: 69/1021 (7%) [Machackova et al., 2019]; 3/14 (21%) [Mateju et al., 2010]<br>Poland: 84/370 (23%) [Cybulski et	One of major founder mutations in Hungary, Austria, Germany, Baltic countries, however, rare outside Central Europe [Janavičius et al., 2010]	Poland: 11/26924 (0.0004) [Brozek et al., 2011]; 2/8000 (0.00025) [Górski et al., 2005];<br>Russia: 1/10572 (0.0001)	NFE: 7/113480 (0.00006)<br>Bulgarians: 1/2670 (0.00037)	All carriers share the same haplotype [Kaufman et al., 2009]	Slavic or Central European mutation

		al., 2019]; Gdansk, ovarian cancer patients: 2/16 (13%) [Cybulski et al., 2019]; Northern Poland: 9/43 (20%) [Ratajska et al., 2008]					
**BRCA1**<br>NM_007294.4<br><br>c.4035delA (p.Glu1346fs)<br><br>BIC nomenclature: 4153delA<br><br>rs80357711<br>VCV000037560.13<br><br>Pathogenic		Russia: the second most common BRCA1 mutation [Sokolenko et al., 2020]<br>Belarus: 33% [Savanevich et al, 2021]<br>13/79 (16%) [Yanus et al., 2022]<br>Poland: 15/370 (4%) [Cybulski et al., 2019]	Major founder mutation in Baltic countries, e.g.: Lithuania: 196/219 (49%) [Janavičius et al., 2014]	Poland: 1/8000 (0.0001) [Górski et al., 2005]<br>Russia: 1/10776 (0.0001)	NFE: 12/129020 (0.00009)<br>Estonians: 7/4832 (0.00145)<br>Bulgarians: 0/2668	All carriers share the same haplotype. This mutation has most probably emerged in Lithuania around 5th century [Janavičius et al., 2013]	Lithuanian (Baltic) founder mutation; minor recurrent variant in “Northern Slavic” countries (Poland, Belarus, Russia)
**BRCA1**<br>NM_007294.4<br><br>c.1687C>T (p.Gln563Ter)<br><br>BIC nomenclature: 1806C>T<br><br>rs80356898<br>VCV000037426.13<br><br>Pathogenic		Slovenia: 21/112 (19%) [Krajc et al., 2014]	Minor founder mutation in Northern/Central Europe (Sweden, Norway, Austria), occasionally found in North America [Wagner et al., 1998; Meisel et al., 2017]	Russia: 1/10790 (0.0001)	NFE: 7/113222 (0.00006)<br>Swedish: 4/26102 (0.00015)<br>Bulgarians: 0/2666	The same haplotype is shared by Austrian and Swedish carriers [Wagner et al., 1998]	Minor Central European founder mutation with unusually high frequency in Slovenia
**BRCA1**<br>NM_007294.4<br><br>c.843_846delCTCA (p.Ser282Tyrfs*15)<br><br>BIC nomenclature: 962del4		Slovenia: 3/14 (21%) [Gornjec et al., 2019]<br>Croatia: 1/11 (9%) [Levanat et al., 2012]	Occasionally occurs in various European populations [Meisel et al., 2017; Rudaitis et al., 2020]				Minor Central European mutation with unusually high frequency in several Balkan Slavic populations

rs80357919<br>RCV000019253.9<br><br>Pathogenic							
**BRCA1**<br>NM_007294.4<br><br>c.844_850dup<br>(p.Gln284fs)<br><br>BIC nomenclature:<br>969ins7<br><br>rs80357989<br>VCV000055735.3<br><br>Pathogenic		Slovenia: 11/112 (10%) [Krajc et al., 2014]	Occasionally occurs in various European populations [Meisel et al., 2017; Rudaitis et al., 2020]				Minor Central European mutation with high frequency in Balkan Slavic populations (Slovenia)
**BRCA1**<br>NM_007294.4<br><br>c.3695_3699delGTAA (p.Val1234fs)<br><br>Also reported as c.3700_3704delGTAAA<br><br>BIC nomenclature:<br>3819_3823<br>delGTAAA<br>(3819del5)<br><br>rs80357609<br>VCV000037542.14<br><br>Pathogenic		Macedonia: 3/22 (14%) [Jakimovska et al., 2018]<br>Czech Republic: 114/1021 (11%) [Machackova et al., 2019]<br>Poland: 10/370 (3%) [Cybulski et al., 2019]; Gdansk, ovarian cancer patients: 3/16 (19%) [Ratajska et al., 2015]; Northern Poland: 4/43 (9%) [Ratajska et al., 2008]<br><br>Russia: one of minor recurrent mutations [Sokolenko et al., 2020]	Extremely frequent in Albanians of Kosovo and North Macedonia: 14/15 (93%) [Kostovska et al., 2023]<br><br>It is also frequent in Mordovia, a region of Russia inhabitated mostly by people of Non-Slavic ethnic descent [Shubin et al., 2011]<br><br>Occasionally occurs in various European populations (Germany, Norway, etc) [Meisel et al., 2017; Heramb et al., 2018]	Poland: 4/7846 (0.0005) [Brozek et al., 2011]			Minor Slavic (?) mutation<br><br>Albanian founder mutation?<br><br>It has not been investigated whether Albanan and Slavic carriers of this mutation share the same haplotype
**BRCA1**<br>NM_007294.4		Poland: 6/1018 (0.6%) [Cybulski et al., 2019]<br>Russia: rare recurrent allele	Recurrent mutation, occasionally reported in various populations		EAS: 1/19954 (0.00005)<br>NFE: 1/129192		Minor recurrent mutation of unclear origin, probably a

c.5251C > T (p.Arg1751Ter)<br><br>BIC nomenclature: 5370C>T<br><br>rs80357123<br>VCV000055480.10<br><br>Pathogenic		[Sokolenko et al., 2020]	(ClinVar)		(<0.00001)<br>Bulgarians: 0/2670		hotspot; increased frequency in Slavs
**BRCA2**<br>NM_000059.3<br><br>c.658_659del (p.Val220Ilefs*4)<br><br>BIC nomenclature: 886_887delGT/886delGT<br><br>rs80359604<br>VCV000009342.10<br><br>Pathogenic	Hereditary breast and ovarian cancer (HBOC)<br><br>#612555<br><br>AD<br><br>~1:300 (?)<br><br>Fanconi anemia, complementation group D1<br><br>AR	Czech Republic: 14/497 (3%) [Machackova et al., 2019]<br>Poland: 3/51 (6%) [Cybulski et al., 2019]	Major Lithuanian founder mutation (50% of all BRCA2 pathogenic alleles) [Janavičius et al., 2014], occasionally reported in other populations	Russia: 2/10440 (0.0002)	AFR: 3/23770 (0.00013)<br>NFE: 7/126178 (0.00006)<br>Bulgarians: 0/2662	Carriers share the same haplotype [Janavičius et al., 2014]	Lithuanian mutation, which is frequent in neighboring Slavic countries
**BRCA2**<br>NM_000059.3<br><br>c.7806-2A>G<br><br>BIC nomenclature: IVS16-2A>G<br><br>rs81002836<br>VCV000052418.8<br><br>Pathogenic		Slovenia: 15/44 (34%) [Krajc et al., 2014]	Occasionally reported in non-Slavic pateints [Palmero et al., 2018; Singh et al., 2018]				Regional (Slovenian) mutation
**BRCA2**<br>NM_000059.3		Macedonia: 6/27 (22%) [Jakimovska et al., 2018]<br>Poland: 1 case [Balabas et al.,	Occasionally found in non-Slavic, mostly European populations	Russia: 2/10888 (0.0002)			Regional (Macedonian) mutation

c.7879A>T<br>(p.Ile2627Phe)<br><br>BIC nomenclature:<br>8107A>T<br><br>rs80359014<br>VCV000052430.12<br><br>Pathogenic		2010];<br>Slovenia: 1 case [Stegel et al., 2011]	[Rebbeck et al., 2018; Meisel et al., 2017]				
**BRCA2**<br>NM_000059.3<br><br>c.7913_7917del<br>(p.Ala2637_Phe2638insTer)<br><br>BIC nomenclature:<br>8138del5<br><br>Also reported as<br>8141del5<br><br>rs80359686<br>VCV000038126.8<br><br>Pathogenic		Czech Republic: 37/497 (7%) [Machackova et al., 2019]<br>Poland: 3/51 (6%) [Cybulski et al., 2019]	Occasionally reported in European and US patients (ClinVar)				Regional (Western Slavic) mutation
**BRCA2**<br>NM_000059.3<br><br>c.8317_8330del14<br>(p.Ser2773Aspfs)<br><br>rs397507976<br>VCV000052544.1<br><br>Pathogenic		Macedonia: 5/27 (19%) [Jakimovska et al., 2018]	Occasionally reported in European populations (Clinvar)				Regional (Macedonian) mutation

**BRCA2**<br>NM_000059.3<br><br>c.8537_8538del<br>(p.Glu2846Glyfs*22)<br><br>BIC nomenclature:<br>8761delAG<br><br>Also reported as<br>8765delAG<br><br>rs80359714<br>VCV000009328.9<br><br>Pathogenic		Czech Republic: 61/497 (12%)<br>[Machackova et al., 2019]	A hotspot mutation, which independently emerged in Northern Sardinia, Yemenite Jews, and French Canadians [Palomba et al., 2007]				Hotspot mutation, reaching noticeable frequency in various regions of the world due to genetic drift / founder effect.<br><br>Regional (Czech) mutation
**BRCA2**<br>NM_000059.3<br><br>c.9098_9099insA<br>(p.Gln3034fs)<br><br>BIC nomenclature:<br>9326insA<br><br>rs80359747<br>VCV000052747.1<br><br>Pathogenic		Bulgaria: 4/11 (37%) [Dodova et al., 2015]	Occasionally reported in European populations (Clinvar)				Regional (Bulgarian) mutation
**BRCA2**<br>NM_000059.3<br><br>c.9403del<br>(p.Leu3135Phefs*28)<br><br>BIC nomenclature:<br>9631delC<br><br>rs80359760		Czech Republic: 13/497 (3%)<br>[Machackova et al., 2019]<br>Poland: 5/51 (10%)<br>[Cybulski et al., 2019]<br>Slovakia: 2/12 (17%) [Konecny et al., 2011]	Occasionally reported in European populations (Clinvar)				Regional (Western Slavic) mutation

VCV000052831.7<br><br>Pathogenic							
**BRCA2**<br>NM_000059.3<br><br>c.5286T>G (p.Tyr1762*)<br><br>BIC nomenclature: 5514T>A<br><br>rs80358754<br>VCV000051836.15<br><br>Pathogenic		Russia: 17/238 (7%) [Kechin et al., 2023]<br>Russia (except Arkhangelsk Oblast): 13/574 (2.3%)<br>Russia (Arkhangelsk Oblast): 7/15 (46.7%) [Yanus et al., 2024]	Occasionally reported in European populations (Clinvar)	Russia: 3/10712 (0.0003)		Carriers share the same haplotype [Kechin et al., 2023]	Regional (Northern Russian?) mutation
**C2**<br>NM_000063.5<br><br>c.841_849 + 19del (p.Val281Profs)<br><br>rs9332736<br>VCV000050634.7<br><br>Pathogenic / Likely pathogenic	C2 complement deficiency<br><br>#217000<br><br>AR<br><br>1:10000 - 1:20000 [Grumach and Kirschfink, 2014]	Slovenia: 17/20 (85%) of all pathogenic alleles [Blazina et al., 2018]	Major mutation in Caucasians, e.g., France: 117/128 (95%) of all pathogenic alleles [El Sissy et al., 2019]	Russia: 33/1794 (0.0184) [Yanus et al., 2019]	ASJ: 120/10364 (0.01158)<br>NFE: 923/128928 (0.007159)<br>Estonians: 86/4822 (0.01783)<br>Bulgarians: 12/2668 (0.004498)	More than 90% patients share the same haplotype [Johnson et al., 1992]	Pan-European mutation of Balto-Slavic origin?<br><br>Frequency of this pan-European mutation is higher in Russians and Estonians than in NFE
**C8B**<br>NM_000066.3<br><br>c.1282C>T (p.Arg428*)<br><br>Also reported as p.R437X<br><br>rs41286844<br>VCV000017038.5<br><br>Pathogenic	Complement component 8B deficiency<br><br>#613789<br><br>AR<br><br>Frequency is unknown<br><br>The combined prevalence of C8B	Russia: 90% [Saucedo et al. 1995]<br>Slovenia: 18/18 (100%) [Blazina et al., 2018]	France: 13/24 (54%) [El Sissy et al., 2019]<br>USA: 74% of all pathogenic alleles [Saucedo et al. 1995]	Russia: 10/1794 (0.00557) [Yanus et al., 2019]; 77/10866 (0.0071) (Ruseq)	NFE: 244/128964 (0.001892)<br>Bulgarians: 20/2668 (0.007496)		Pan-European mutation of Slavic origin?<br><br>Frequency of this mutation is higher in Slavic populations than in NFE

	and C7 deficiencies approached 1:8333 in former USSR [Platonov et al. 1993]						
**C19Orf12** NM_001031726.3<br><br>c.204_214del11 (p.Gly69Argfs)<br><br>Also reported as c.171_181del (p.Gly58fs)<br><br>rs515726204 VCV000031155.5<br><br>Pathogenic	Neurodegeneration with brain iron accumulation 4<br><br>#614298<br><br>AR	Poland: 29/38 (76%) [Hartig et al., 2011]<br>Czech Republic: 6/8 (80%) [Dusek et al., 2020]<br>Russia: 2/2 (100%) [Zakharova et al, 2014]<br>Reported in Bosnia, Ukraine [Hogarth et al., 2013]	Never reported in patients without East European ancestry	Russia: 12/10930 (0.0011)	NFE: 26/126906 (0.0002)<br>Estonians: 10/4820 (0.00207)<br>Bulgarians: 1/2668 (0.00037)	All carrier shared the same haplotype [Hartig et al., 2011]	Balto-Slavic mutaton
**CAPN3** NM_000070.3<br><br>c.550delA (p.Thr184Argfs)<br><br>rs80338800 VCV000017621.14<br><br>Pathogenic	Limb-girdle muscular dystrophy, type 2A<br><br>#253600<br><br>AR<br><br>1:100000 – 9:100000	Bulgaria: 22/37 (59%) [Todorova et al., 2007]<br>Croatia: 43/58 (74%) [Milic et al., 2005]<br>Serbia: 27/38 (71%) [Peric et al., 2019]<br>Czech Republic: 10/26 (38%) [Stehlíková et al., 2017]<br>Czech Republic: 2/12 (17%) [Chrobáková et al., 2004]<br>Poland: 23/44 (52%) [Fichna et al., 2018]<br>Russia: 16/30 (53%) [Pogoda et al., 2000]	Frequent in North-Eastern Italy, Turkey, Germany [Richard et al., 1999]	Croatia: 0.0017 [Canki-Klain et al., 2004]<br>Poland: 0.004 [Dorobek et al., 2015]<br>Russia: 0.0033 [Pogoda et al., 2000]; 30/10926 (0.0027) (Ruseq)		CAPN3 c.550delA allele was found on 5 different haplotypes, but 3 of the most prevalent ones are closely related, so 66-82% of 550delA alleles show a common origin [Milic et al., 2005]	Slavic mutation
**CA5A** NM_001739.2<br><br>c.555G>A (p.Lys185=: this substitution affects a splice site and leads to exon 4 skipping, p.Leu154_Lys185del)	Hyperammonemia due to carbonic anhydrase VA deficiency<br><br>#615751<br><br>AR<br><br>Roughly two dozen		Never reported in non-Russian patients	Russia: 0.0047 [Semenova et al., 2022]; 27/10632 (0.0025) (Ruseq)	NFE 42/128746 (0.0003262)<br>Bulgarians: 2/2668 (0.0007496)<br>Estonians: 3/4774 (0.0006284)		Regional (Russian?) mutation

rs147623570<br>VCV000127088.8<br><br>Likely pathogenic / Uncertain significance	of patients have been reported to date [Semenova et al., 2022]						
**CDKN2A**<br>NM_000077.5<br><br>c.307_308del (p.Arg103fs)<br><br>rs886041162<br>VCV000279749.8<br><br>Pathogenic	Melanoma-pancreatic cancer syndrome<br><br>#606719<br><br>AD	Russia: 3/6 (50%) [Danishevich et al., 2023]	Several cases outside Russia have been reported (ClinVar)				Regional (Russian) mutation
**CEP290**<br>NM_025114.4<br><br>c.5493del (p.Ala1832fs)<br><br>rs386834158<br>VCV000056739.32<br><br>Pathogenic	Joubert syndrome 5<br><br>Meckel syndrome 4<br><br>Senior-Loken syndrome 6<br><br>#610188 #611134 #610189<br><br>AR<br><br>Incidence of all forms of nephronophthisis is about 1:80000 (Finland)	Kosovo and Albania: 3 unrelated families [Spahiu et al., 2023]<br><br>Albanians are not considered to have Slavic ancestry. Still, relatively high population frequency of this allele in Slavs (especially Bulgarians) suggests Slavic origin of this mutation.	Occasionally reported in non-Slavic patients	Russia: 1/10414 (0.0001)	NFE: 5/102202 (0.00004892)<br>Bulgarians: 3/2582 (0.001162)		Regional (Southern Slavic?) mutation<br><br>Albanian mutation?
**CFAP300** (formerly C11orf70)<br>NM_032930.3<br><br>c.198_200delTTTinsCC (p.Phe67Profs)<br><br>rs1555069023	Ciliary dyskinesia, primary, 38<br><br>#618063<br><br>AR<br><br>All primary ciliary	Poland: 31/34 (91%) [Zietkiewicz et al., 2019]	One Ashkenazi Jewish and one German carriers have been reported [Höben et al., 2018]		Finns: 141/24690 (0.00571)<br>NFE: 33/123438 (0.00027)<br>Bulgarians: 4/2612 (0.00153)<br>Estonians: 4/4794 (0.00083)		Balto-Slavic (?) or Finnish (?) mutation

VCV000549862.1<br><br>Pathogenic	dyskinesias combined: 1:15000 – 1:30000						
**CFTR**<br>NM_000492.3<br><br>c.54-5940_273+10250del<br><br>Also reported as CFTRdelex2,3<br><br>VCV000066105.5<br><br>Pathogenic	Cystic fibrosis<br><br>#219700<br><br>AR<br><br>1:3000 in Europe (1:400-1:25000)	Macedonia: 5/316 (1.6%) [Terzic et al., 2019]<br>Ukraine: 11/221 (5%) [Makukh et al., 2010]<br>Slovakia: 19/550 (3%) [Soltysova et al., 2018]<br>Czech Republic: 69/1200 (6%) [Křenková et al., 2013]; 6.4% [Dörk et al., 2000]<br>Poland: 66/1476 (4%) [Ziętkiewicz et al., 2014]; 1.5% [Dörk et al., 2000]<br>Russia: 5.2% [Dörk et al., 2000]<br>Belarus: 3.3% [Dörk et al., 2000]	Austria (2.6%), Germany (1.5%) [Dörk et al., 2000]		NFE: 1/7624 (0.00013)	All carriers share the same haplotype [Dörk et al., 2000]	Western and Eastern Slavic / Central European mutation
**CFTR**<br>NM_000492.3<br><br>c.2052dupA (p.Gln685Thrfs)<br><br>Also reported as 2184insA; c.2052_2053insA<br><br>rs121908746<br>VCV000035838.36<br><br>Pathogenic	Cystic fibrosis<br><br>#219700<br><br>AR<br><br>1:3000 in Europe (1:400-1:25000)	Ukraine: 19/221 (9%) [Makukh et al., 2010]<br>Slovakia: 10/550 (2%) [Soltysova et al., 2018]	Occasionally reported in other European populations	Russia: 1/1450 (0.0007)	SAS: 2/30558 (0.00007)<br>NFE: 4/112124 (0.00004)<br>Bulgarians: 1/2660 (0.00038)		Regional (Ukrainian) mutation
**CHEK2**<br>NM_007194.4<br><br>c.1100del (p.Thr367Metfs)<br><br>Also reported as p.Thr410Metfs<br><br>rs555607708	Low penetrance breast, kidney, thyroid and testicular cancer predisposition<br><br>#114480<br><br>AD	Poland: 8/37 (22%) [Cybulski et al., 2019]<br>Czech Republic: 3/7 (43%) [Walsh et al., 2006]; 7/61 (12%) [Kleiblova et al., 2019].<br>Slovenia: 9/49 (18%) [Nizic-Kos et al., 2020]	Very frequent variant worldwide (Clinvar)	Poland: 10/8000 (0.00125) [Górski et al., 2005]	Finns: 219/25124 (0.00872)<br>NFE: 327/127908 (0.00256)<br>Swedish: 99/26086 (0.0038)<br>Bulgarians: 2/2658 (0.00075)	All carriers share the same haplotype [CHEK2 Breast Cancer Case-Control Consortium, 2004]	North-Eastern European founder mutation, with a particularly high frequency in Finland and Sweden; also very common in Slavic populations

VCV000128042.108<br><br>Pathogenic / Uncertain significance							
**CHEK2**<br>NM_007194.4<br><br>c.(908+1_909-1)_(1095+1_1096-1)del<br><br>Also reported as 5395 bp deletion; 5567 deletion; deletion of exons 9 and 10; L303Fdel304_391<br><br>Not reported in dbSNP<br>VCV000584576.2<br><br>Pathogenic		Poland: 14/37 (38%) [Cybulski et al., 2019]<br>Czech Republic: 2/7 (29%) [Walsh et al., 2006]; 18/61 (30%) [Kleiblova et al., 2019]<br>Slovenia: 6/49 (12%) [Nizic-Kos et al., 2020]<br>Serbia: at least one case [Krivokuca et al., 2013]	This mutation is observed in Latvia, and, at a very low frequency, in Germany [Hackmann et al., 2016; Plonis et al., 2016]	Poland: 24/10992 (0.0022) [Cybulski et al., 2007]	Europeans: 1/7624 (0.00013)	All carriers share the same haplotype [Walsh et al., 2006]	Slavic mutation
**CHEK2**<br>NM_007194.4<br><br>c.444+1G>A<br><br>Also reported as IVS3+1G>A; IVS2+1G>A<br><br>rs121908698<br>VCV000128075.90<br><br>Pathogenic / Likely pathogenic		Poland: 13/37 (35%) [Cybulski et al., 2019]<br>Czech Republic: 5/61 (8%) [Kleiblova et al., 2019]<br>Slovenia: 15/49 (31%) [Nizic-Kos et al., 2020]	Occasionally reported in several European countries (e.g. Finland, Germany) [Dufault et al., 2004; Nurmi et al., 2019]	Poland: 14/8000 (0.00175) [Górski et al., 2005]<br>Russia: 11/10882 (0.001)	NFE: 1/113574 (<0.00001)<br>Bulgarians: 1/2668 (0.00037)		Slavic mutation
**CHST6**<br>NM_021615.5	Macular corneal dystrophy	Czech Republic: 7/12 (58%) [Liskova et al., 2008]<br>Poland: 4/9 (44%) [Nowinska et	This mutation is a pan-European founder allele, but it is more frequent in	Russia: 3/10790 (0.0003)	NFE: 59/125324 (0.00047)<br>Bulgarians: 2/2628	This mutation is usually found in cis to	Pan-European mutation, which shows unusually high

c.599T>G (p.Leu200Arg)<br><br>rs28937879 VCV000005075.6<br><br>Pathogenic	#217800<br><br>AR<br><br>1:100000 - 9:100000	al., 2014]	certain Slavic countries, compared to others [El-Ashry et al., 2002; Gruenauer-Kloevekorn et al., 2008]		(0.00076)	p.Arg162Gly polymorphism, indicating shared haplotype	prevalence in certain Slavic countries
**CLDN16** NM_006580.3<br><br>c.453G>T (p.Leu151Phe)<br><br>rs104893729 VCV000005934.6<br><br>Pathogenic / Uncertain significance	Hypomagnesemia 3, renal (Familial hypomagnesaemia with hypercalciuria and nephro-calcinosis (FHHNC))<br><br>#248250<br><br>AR<br><br><1:1000000	Poland: 26/36 (72%) [Sikora et al., 2014]<br>Serbia: 4/4 (100%) [Peco-Antić et al., 2010]<br>Macedonia: 2/2 (100%) [Tasic et al., 2005]	This mutation is frequent in Germany and Eastern Europe [Weber et al., 2001]	Russia: 1/10900 (0.00001)	OTH: 1/7226 (0.00014)<br>NFE: 17/129188 (0.00013)<br>Bulgarians: 2/2670 (0.00075)	All carriers share the same haplotype	Central European (German) and Slavic founder mutation
**COL4A5** NM_033380.3<br><br>c.1871G>A (p.Gly624Asp)<br><br>rs104886142 VCV000024455.12<br><br>Pathogenic / Likely pathogenic	X-linked Alport syndrome-1 (benign familial hematuria; thin basement membrane nephropathy)<br><br>#301050<br><br>p.Gly624Asp is a hypomorphic allele, but 10-15% patients proceed to end-stage kidney disease<br><br>1:100000 – 9:100000	Slovenia: 6/17 (35%) families [Slajpah et al., 2007]<br>Poland: 44/113 (39%) [Żurowska et al., 2021]<br>Russia: 7/44 (16%) families [Shagam, 2017]	Common in South-Eastern Europe: Cyprus: 2/6 (33%); Hungary: 3/10 (30%); occurs in other populations of European descent Australia: 2/18 (11%) USA: 1/41 (2%) [Martin et al., 1998; Demosthenous et al., 2012; Kovács et al., 2016; Mallett et al., 2017]	Russia: 13/10828 (0.0012)	NFE: 16/81695 (0.0002)<br>Bulgarians: 1/1980 (0.00051)	All carriers share the same haplotype haplotype [Demosthenous et al., 2012; Żurowska et al., 2021]	Slavic / Balkan founder mutation
**COL7A1** NM_000094.4	Dystrophic epidermolysis bullosa	Czech Republic: 12/80 (16%) [Kopečková et al., 2016]; 6/54 (30%) [Jerábková et al., 2010]	Relatively frequent in Germany and Hungary: 11 of 86 alleles (13%)	Russia: 2/10878 (0.0002)			Central European (?) or Slavic (?) mutation

c.425A>G (p.Lys142Arg)<br><br>VCV000029636.46<br>rs121912856<br><br>Pathogenic	#226600<br><br>AR<br><br>1:1000000 - 9:1000000	Poland: 25/68 (37%) [Wertheim-Tysarowska et al., 2012]<br>Russia: 34/332 (10%) [Savostyanov et al., 2022]	[Csikós et al., 2005]. Rare outside Central/Eastern Europe				
**COL7A1**<br>NM_000094.4<br><br>c.682+1G>A<br><br>rs775288140<br>VCV000372329.32<br><br>Pathogenic / Likely pathogenic		Czech Republic: 2/54 (4%) [Jerábková et al., 2010]<br>Poland: 6/68 (9%) [Wertheim-Tysarowska et al., 2012]<br>Russia: 19/332 (6%) [Savostyanov et al., 2022]	Occasionally reported outside Slavic populations (ClinVar)	Russia: 5/10874 (0.0005)	NFE: 5/129080 (0.00003874)<br>Estonians: 1/4820 (0.0002075)		Central European (?) or Slavic (?) mutation
**COL7A1**<br>NM_000094.4<br><br>c.6146G>A (p.Gly2049Glu)<br><br>rs1410793870<br>VCV001047980.2<br><br>Pathogenic		Czech Republic: 6/80 (8%) [Kopečková et al., 2016]; 5/54 (9%) [Jerábková et al., 2010]	Not reported outside Czech Republic	Russia: 0/1600			Regional (Czech?) mutation
**CYP4F22**<br>NM_173483.4<br><br>c.59dupG (p.Ile21Hisfs)<br><br>rs531800013<br>VCV000279799.26<br><br>Pathogenic	Autosomal recessive congenital ichthyosis-5 (unusual nonlamellar, nonerythrodermic ichthyosis phenotype)<br><br>#604777<br><br>AR	Czech Republic: 5/10 (50%) [Borská et al., 2019]	This mutation was reported in a large Scandinavian cohort [Pigg et al., 2016]		NFE: 22/129112 (0.00017)<br>Bulgarians: 1/2670 (0.00037)		Regional (Czech?) mutation

	1:1000000 -<br>9:1000000						
**CYP24A1**<br>NM_000782.5<br><br>c.1186C>T<br>(p.Arg396Trp)<br><br>rs114368325<br>VCV000029679.5<br><br>Pathogenic	Idiopathic infantile hypercalcemia<br><br>#143880<br><br>AR<br><br><1:1000000	Poland: 11/18 (61%) [Pronicka et al., 2017]<br>Russia: 5/10 (50%) [Tikhonovich et al, 2017]	Italy: 5/14 (36%) [Gigante et al., 2016];<br>France: 3/14 (21%) [Figueres et al., 2015];<br>Germany:1/6 (17%) [Schlingmann et al., 2011]	Russia:<br>63/10778<br>(0.0058)	Finns: 37/24972 (0.00148)<br>NFE: 148/129106 (0.00115)<br>Bulgarians: 12/2670 (0.00449)		Pan-European mutation with an unusually high prevalence in Slavic populations
**DGUOK**<br>NM_080916.3<br><br>c.3G>A (p.Met1?)<br><br>rs534297082<br>VCV001324223.4<br><br>Pathogenic	Mitochondrial DNA depletion syndrome 3 (hepatocerebral type)<br><br>#251880<br><br>AR<br><br><1:1000000	Poland: 7/10 (70%) [Jankowska et al., 2020]<br>Poland: 3/7 (43%) [Pronicka et al., 2011]<br>Russia: 10/11 (91%) [Bychkov et al, 2021]	Never reported in non-Polish patients	Russia:<br>10/10910<br>(0.0009)	OTH: 1/7212 (0.00014)<br>NFE: 6/128898 (0.00005)<br>Bulgarians: 2/2670 (0.00075)		Slavic mutation
**DGUOK**<br>NM_080916.3<br><br>c.494A>T<br>(p.Glu165Val)<br><br>rs770950831<br>VCV000595262.1<br><br>Pathogenic		Poland: 2/7 (29%) [Pronicka et al., 2011]	Never reported in non-Polish patients	Russia:<br>1/10918<br>(0.0001)	NFE: 2/113764 (0.00002)<br>Bulgarians: 0/2670		Regional (Polish?) mutation
**DHCR7**<br>NM_001360.3<br><br>c.452G>A<br>(p.Trp151*)<br><br>rs11555217<br>VCV000021273.15	Smith-Lemli-Opitz syndrome<br><br>#270400<br><br>AR<br><br>1:20000 – 1:40000	Czech Republic: 11/25 (44%) [Blahakova et al., 2007]<br>10/20 (50%) [Kozák et al., 2000]<br>Poland: 10/30 (33%) [Witsch-Baumgartner et al., 2001]	Relatively frequent in Germany (17%), USA (10%), rarely found in other European populations [Witsch-Baumgartner et al., 2008]	Carrier frequencies:<br>Poland: 1:83<br>Czech Republic: 1:56<br>[Witsch-Baumgartner et al., 2008]<br>MAF in Russia:	NFE: 181/127446 (0.00142)<br>Bulgarians: 12/2662 (0.00451)	All carriers share the same haplotype [Witsch-Baumgartner et al., 2008]	Slavic mutation

Pathogenic / Likely pathogenic				62/10924 (0.0057)			
**DHCR7**<br>NM_001360.2<br><br>c.976G>T (p.Val326Leu)<br><br>rs80338859<br>VCV000006785.6<br><br>Pathogenic		Czech Republic:<br>5/20 (25%) [Kozák et al., 2000]<br>Poland: 7/30 (23%) [Witsch-Baumgartner et al., 2001]	Rarely identified in non-Slavic populations [Witsch-Baumgartner et al., 2008]	Russia:<br>6/10528 (0.0006)	NFE: 4/105792 (0.00003)<br>Bulgarians: 1/2082 (0.00048)	All carriers share the same haplotype [Witsch-Baumgartner et al., 2008]	Slavic mutation
**DNAI1**<br>NM_012144.4<br><br>c.1612G>A (p.Ala538Thr)<br><br>rs368248592<br>VCV000583106.3<br><br>Pathogenic	Primary ciliary dyskinesia, type 1<br><br>#244400<br><br>AR<br><br>1:15000-1:30000 (all forms)	Poland: 8/22 (36%) [Ziętkiewicz et al., 2010]	Rarely reported outside Slavic populations [Zariwala et al., 2006]	Russia:<br>2/10914 (0.0002)	NFE: 18/129040 (0.00014)<br>Bulgarians: 3/2670 (0.00112)		Slavic mutation
**DNAJC30**<br>NM_032317.3<br><br>c.152A>G (p.Tyr51Cys)<br><br>rs61732167<br>VCV000976691.5<br><br>Pathogenic	Leber hereditary optic neuropathy<br><br>#619382<br><br>AR<br><br>1:27:000 – 1:54000 (in Europe)	Russia: 52/52 (100%)<br>Czech Republic: 30/30 (100%)<br>Poland: 6/6 (100%)<br>Ukraine: 6/6 (100%)<br>[Stenton et al., 2021; Stenton et al., 2022]	Sometimes detected in patients from Europe (Romania, Germany, etc), USA, Canada and Turkey	Russia:<br>10/2072 (0.0048) [Stenton et al., 2021];<br>69/8204 (0.0084) (Ruseq)	Finns: 119/24972 (0.00477)<br>ASH: 34/10320 (0.0033)<br>NFE: 187/128340 (0.0015)<br>Estonians: 40/4822 (0.008)<br>Bulgarians: 11/2668 (0.004)	All carriers share the same haplotype. This allele have emerged ~85 generations ago	Eastern/Western Slavic mutation
**EPCAM**<br>NM_002354.2<br><br>c.858 + 2478_*4507del	Lynch syndrome<br><br>#120435<br><br>AD<br><br>Prevalence is	Poland: 5% Lynch syndrome pedigrees (8 families) [Dymerska et al., 2017]	Several cases also reported in Hungary and Netherlands	Russia:<br>0/1600		All carriers share the same haplotype [Dymerska et al., 2017]	Regional (Polish) mutation

Not reported in dbSNP<br>Not reported in ClinVar<br><br>Uncertain significance / Pathogenic	variable across Europe						
**F8**<br>NM_000132.4<br><br>c.1901A>G (p.His634Arg)<br><br>Not reported in dbSNP<br>Not reported in ClinVar<br><br>Uncertain significance / Likely pathogenic	Hemophilia A<br><br>#306700<br><br>XLR<br><br>1:6000 males	Russia (Sverdovskaya oblast: Middle and Northern Ural): 7/28 (25%) [Salomashkina et al., 2022]	Not reported outside Ural			All carriers share the same haplotype, suggesting emergence of mutation in XVII century in local inhabitants [Lipari et al., 2020]	Regional Russian mutation (Sverdlovsk / Ekatherinburg)
**FHOD3**<br>NM_001281740.3<br><br>c.1646+2T>C<br><br>Not reported in dbSNP<br>VCV001805290.6<br><br>Uncertain significance / Likely pathogenic [Vodnjov et al., 2023]	Familial hypertophic cardiomyopathy (fHCM), type 28<br><br>#619402<br><br>AD<br><br>Up to 1:200-1:500 (all varieties of fHCM)	Slovenia (all carriers had ancestors from Bosnia, Serbia and Montenegro): 8/8 (100%); 8/51 (16%) of all fHCM cases with known genetic cause [Vodnjov et al., 2023]	Occasionally identified in various populations			Slovenian carriers share the same haplotype [Vodnjov et al., 2023]	Regional (South-Western Slavic) mutation
**FRAS1**<br>NM_025074.7<br><br>c.6963_6964dup (p.Val2322Glyfs)<br><br>AR	Fraser syndrome<br><br>#219000<br><br>AR	Poland: 5/10 (50%) (2 families from Poland, 3 compound heterozygous probands with one or both parents originating from Poland) [Midro et al., 2020]	Two of four cases reported outside Poland were definitely of Polish origin, in two others ethnic descent was not reported [Midro et al.,		Finns: 1/25024 (0.00004)<br>NFE: 2/128100 (0.00002)<br>Estonians: 2/4832 (0.00041)		Regional (Polish?) mutation

rs730882179<br>VCV000002815.22<br><br>Pathogenic	Prevalence is unknown		2020]		Bulgarians: 0/2662		
**GCK**<br>NM_000162.5<br><br>c.118G>A<br>(p.Glu40Lys)<br><br>rs794727236<br>VCV000195024.1<br><br>Pathogenic	MODY, type II / Diabetes mellitus, permanent neonatal 1<br><br>#125851 / #606176<br><br>AD / AR<br><br>1:10000 (all MODY types combined)	Czech Republic: 21/152 (14%) [Dusatkova et al., 2012]	This mutation was also reported in Japan [Kawakita et al., 2014]			All carriers share the same haplotype [Dusatkova et al., 2012]	Regional (Czech) mutation
**GCK**<br>NM_000162.5<br><br>c.944T>A<br>(p.Leu315His)<br><br>rs193922338<br>VCV000036266.1<br><br>Pathogenic		Czech Republic: 15/152 (10%) [Dusatkova et al., 2012]	Never reported outside Czech Republic			All carriers share the same haplotype [Dusatkova et al., 2012]	Regional (Czech) mutation
**GCK**<br>NM_000162.5<br><br>c.127C>T<br>(p.Arg43Cys)<br><br>rs1486280029<br>VCV000585911.1<br><br>Pathogenic		Poland: 5/68 (7%) [Borowiec et al., 2012]	Occasionally reported in non-Slavic subjects				Regional (Polish) mutation
**GCK**<br>NM_000162.5<br><br>c.952G>A<br>(p.Gly318Arg)		Czech Republic: 13/152 (9%) [Dusatkova et al., 2012]<br>Southern Poland: 13/68 (19%) [Dusatkova et al., 2012]<br>Poland: 9/68 (13%) [Borowiec et al., 2012]	Occasionally reported in non-Slavic subjects		Finns: 1/18916 (0.00005)<br>NFE: 0/112934	All carriers share the same haplotype [Dusatkova et al., 2012]	Regional (Czech and Southern Polish) mutation

rs193922340<br>VCV000585929.1<br><br>Pathogenic							
**GCK** NM_000162.5<br><br>c.98T>C<br>(p.Val33Ala)<br><br>rs1554335954<br>VCV000585930.1<br><br>Pathogenic		Czech Republic: 10/152 (7%) [Dusatkova et al., 2012]	Never reported outside Czech Republic			Controversial results of haplotyping [Dusatkova et al., 2012]	Regional (Czech) recurrent mutation (founder or hotspot?)
**GDAP1**<br>NM_018972.4<br><br>c.715C>T<br>(p.Leu239Phe)<br><br>rs104894080<br>VCV000004200.42<br><br>Pathogenic / Likely pathogenic	Charcot–Marie–Tooth, type 4A disease<br><br>#214400<br><br>AR<br><br><1:1000000	Poland: 8/12 (67%) [Kabzińska et al., 2010]<br>Russia: 5/8 (63%) [Shagina et al., 2010]	Occasionally reported in several European countries (e.g. Germany, Italy) [Ammar et al., 2003; Kabzińska et al., 2010]	Russia: 8/10904 (0.0007)	NFE: 10/129150 (0.00008)<br>Southern Europeans: 2/11606 (0.00017)<br>Bulgarians: 0/2668	7 Polish, 6 Czech, 1 Bulgarian, family, and several German and Italian families shared the same haplotype [Kabzińska et al., 2010]	Slavic mutation
**GJB1**<br>NM_000166.6<br><br>c.94A>G<br>(p.Arg32Gly)<br><br>rs1602348720<br>VCV000637614.1<br><br>Uncertain significance / Likely pathogenic	Demyelinating Charcot-Marie-Tooth (CMT1) disease<br><br>#302800<br><br>XLD<br><br>1:2500 (all CMT varieties)	Serbia: 5/10 (50%) [Keckarevic Markovic et al., 2013]	Never reported outside Serbia			All carriers share the same haplotype [Keckarevic Markovic et al., 2013]	Regional (Serbian) mutation
**GNE**<br>NM_001128227.3<br><br>c.1760T>C	Nonaka myopathy<br><br>#605820	Russia: 6/54 (11%) [Murtazina et al., 2022]	One case reported outside Russia				Regional (Russian) mutation

(p.Leu587Ser)<br><br>Also reported as c.1667T>C (p.Leu556Ser)<br><br>Not reported in dbSNP<br>Not reported in ClinVar<br><br>Uncertain significance / Likely pathogenic	AR<br><br>1:1000000						
**GNRHR**<br>NM_000406.3<br><br>c.416G>A (p.Arg139His)<br><br>rs104893842<br>VCV000016030.4<br><br>Pathogenic	Hypogonadotropic hypogonadism 7 without anosmia (normosmic congenital hypogonadotropic hypogonadism)<br><br>#146110<br><br>AR<br><br>1:29000 in males, 1:130000 in females (all forms)		Occasionally reported in non-Slavic subjects; it is the most frequent GNRHR mutation in Brazil [Choi et al., 2015]	Russia: 25/10886 (0.0023)	Finns: 14/25082 (0.00056)<br>NFE: 26/128682 (0.0002)<br>Bulgarians: 5/2668 (0.0019)	8 of 15 reported patients with R139H mutation in GNRHR were of Polish origin; they shared the same haplotype. The Brazilian carriers have different haplotype [Choi et al., 2015]	Slavic founder mutation (the same variant occurs in Brazil, but it has an independent origin)
**HADHA**<br>NM_000182.5<br><br>c.1528G>C (p.Glu510Gln)<br><br>rs137852769<br>VCV000100085.14<br><br>Pathogenic	Long chain 3-hydroxyacyl-CoA dehydrogenase deficiency<br><br>#609016<br><br>AR<br><br>1:250000 - 1:120000 in Poland and 1:20000 in	Ukraine: 6/6 (100%) [Barvinska et al., 2018]	This is a major founder mutation in European countries, e.g. 51/90 (57%) alleles in French study [Boutron et al., 2011].<br><br><br>Populational frequency is high in Estonia and Finland, somewhat lower in the Netherlands	Carrier frequency: 1:57 – 1:73 in Kashubians (Pomerania); 1:187 – 1:217 in other Polish regions [Piekutowska-Abramczuk et al., 2010; Nedoszytko et al., 2017];	FIN 98/25122 (0.0039)<br>NFE: 216/129166 (0.00167)<br>Estonians: 29/4830 (0.006)<br>Bulgarians: 5/2670 (0.00187)	All carriers share the same haplotype [Piekutowska-Abramczuk et al., 2010]	Regional (Kashubian) mutation, spreading to neighboring countries. Very rare elsewhere, but is still the most prevalent in Europe

	Pomerania		[Nedoszytko et al., 2017]	1:150 in Russia [Yanus et al., 2019]<br>MAF in Russia: 33/10912 (0.003)			
**HGD**<br>NM_000187.4<br><br>c.481G>A (p.Gly161Arg)<br><br>Also reported as c.648G>A (p.Gly161Arg)<br><br>rs28941783<br>VCV000003168.29<br><br>Pathogenic	Alkaptonuria<br><br>#203500<br><br>AR<br><br>Prevalence is 1:250000 - 1:1000000 worldwide, but up to 1:19000 in Slovakia	Slovakia: 23/58 (40%) [Srsen et al., 2002]	Recurrent mutation, which is observed in several European countries (UK, France) [Vilboux et al., 2009; Usher et al., 2015]	Russia: 21/10896 (0.0019)	OTH: 2/7214 (0.00028)<br>NFE: 33/128846 (0.00026)<br>Estonians: 13/4822 (0.0027)<br>Bulgarians: 4/2670 (0.0015)	Croatian carriers share the same haplotype (with a recombination event in some alleles) [Müller et al., 1999; Zatková et al., 2012]	Regional (Slovak / Czech / Balto-Slavic ?) mutation
**HGD**<br>NM_000187.4<br><br>c.1111dupC (p.His371Profs)<br><br>Also reported as c.1278insC (p.Pro370fs)<br><br>rs397515516<br>VCV000065573.5<br><br>Pathogenic		Slovakia: 10/58 (17%) [Srsen et al., 2002]	Several instances of this mutation were also reported in USA (ClinVar)	Russia: 0/1600	NFE: 1/113570 (<0.00001)<br>NWE: 1/42100 (0.00002)<br>Bulgarians: 0/ 2670	Croatian carriers share the same haplotype (with a recombination event in some alleles) [Müller et al., 1999; Zatková et al., 2012]	Regional (Slovak) mutation
**HGD**<br>NM_000187.4<br><br>c.457dupG (p.Asp153GlyfsX26)		Slovakia: 8/58 (14%) [Srsen et al., 2002]	Several carriers of this mutation were identified in France, Italy, USA [Zatková et al., 2012]	Russia: 0/1600	Latino: 3/35436 (0.00008)<br>NFE: 7/128996 (0.00005) Southern Europeans: 2/11596	Croatian carriers share the same haplotype (with a recombination event in some	Regional (Slovak) mutation

Also reported as c.625insG (p.Gly152fs)<br><br>rs397515346 VCV000003169.10<br><br>Pathogenic					(0.00017)<br>Bulgarians: 0/2668	alleles) [Müller et al., 1999; Zatková et al., 2012]	
**HGD** NM_000187.4<br><br>c.16-1G>A (p.Tyr6_Gln29del)<br><br>Also reported as c.183-1G>A; IVS1-1G>A<br><br>rs397515347 VCV000003170.11<br><br>Pathogenic		Slovakia: 6/58 (10%) [Srsen et al., 2001]	Several carriers of this mutation were identified in Algeria, USA [Zatková et al., 2012]	Russia: 6/10830 (0.0006)	NFE: 5/113414 (0.00004)<br>OTH: 4/30880 (0.00013)<br>Bulgarians: 0/ 2668	Croatian carriers share the same haplotype [Zatková et al., 2000]	Slavic (?) mutation
**HINT1** NM_005340.7<br><br>c.110G>C (p.Arg37Pro)<br><br>rs149782619 VCV000037312.54<br><br>Pathogenic	Hereditary axonal neuropathy accompanied by neuromyotonia<br><br>#137200<br><br>AR<br><br><1:1000000	Czech Republic: 36/38 (95%) [Laššuthová et al., 2015]<br>Russia: 60/62 (97%) [Shchagina et al., 2020]	Very frequent in Latvia: 12/16 (75%) [Malcorps et al., 2022]<br><br>Occasionally reported in several non-Slavic / non-Baltic European countries (ClinVar)	Carrier frequency: Czech Republic: 1:182 [Laššuthová et al., 2015]<br>Russia: 1:250 [Shchagina et al., 2020]<br>MAF in Russia: 33/10698 (0.0031)	Finns: 22/24748 (0.00089)<br>NFE: 59/128474 (0.00046)<br>Bulgarians: 10/2670 (0.00375)<br>Estonians: 15/4812 (0.00312)	All carriers share the same haplotype [Shchagina et al., 2020]	Balto-Slavic mutation
**KCNQ1** NM_000218.2<br><br>c.926C>T	Long QT syndrome<br><br>#192500	Czech Republic: 10/44 (23%) [Synková et al., 2021]	This mutation has previously been described in a Taiwanese LQTS kindred [Ko et al.,			Czech carriers share the same haplotype [Synková et al.,	Regional (Czech) mutation

(p.Thr309Ile)<br><br>rs199472743<br>VCV000053132.3<br><br>Likely pathogenic	AD<br><br>1:7000		2001]			2021]	
**LAMA2**<br>NM_000426.3<br><br>c.9095dupA<br>(p.Ile3033Aspfs)<br><br>rs1554320205<br>VCV000552139.1<br><br>Likely pathogenic	Congenital muscular dystrophy, merosin deficient or partially deficient<br><br>#607855 / #618138<br><br>AR<br><br>1:30000	Czech Republic: 4/16 (25%) [Stehlíková et al., 2017]					Regional (Czech) mutation
**LAMA2**<br>NM_000426.3<br><br>c.799G>A<br>(p.Asp267Asn)<br><br>rs748356668<br>Not reported in ClinVar<br><br>Uncertain significance / Likely pathogenic		Czech Republic: 2/16 (13%) [Stehlíková et al., 2017]	Not reported outside Czech Republic		ASJ: 1/10052 (0.00001)		Regional (Czech) mutation
**LDLR**<br>NM_000527.5<br><br>c.1775G>A<br>(p.Gly592Glu)<br><br>Also reported as p.Gly571Glu; FH Sicily; FH Foggia-1; FH Naples-4; FH Sicilia-4	Familial hypercholesterinemia<br><br>#143890<br><br>AD / AR<br><br>~1:200 – 1:250 (AD)	Czech Republic: 103/535 (19%) [Tichý et al., 2012]<br>Slovakia: 12/89 (13%) [Gabčová et al., 2017]<br>Poland: 38/169 (22%) [Chmara et al., 2010]<br>Russia: 4/24 (17%) [Semenova et al., 2020]; 6/34 (18%) [Miroshnikova et al., 2021]	Relatively frequent in patients from North-Western Greece (34%), Andalusia (11%), Italy and Portugal (3-4%); occasionally detected in other European countries [Dedoussis et al., 2004; Bourbon et al., 2008; Diakou et al., 2011; Palacios et al., 2012;	Russia: 3/10890 (0.0003)	NFE: 15/129176 (0.00012)<br>Bulgarians: 1/2670 (0.00037)		Southern European / Slavic mutation

rs137929307<br>VCV000161271.56<br><br>Pathogenic	1:1000000 –<br>9:1000000 (AR)		Wintjens et al., 2016; Pirillo et al., 2017]				
**LDLR**<br>NM_000527.5<br><br>Exon 4-8 duplication<br>c.314-?_1186+?dup<br>(p.Pro106_Val395du p)<br><br>Not reported in dbSNP<br>Not reported in ClinVar<br><br>Uncertain significance / Likely pathogenic		Poland: 16/169 (9%) [Chmara et al., 2010]<br>Czech Republic: 2/535 (0.4%) [Dusatkova et al., 2012]; 2/89 (2%) [Gabčová et al., 2017]	1 case reported in Netherlands [van der Graaf et al., 2011]				Regional (Polish) Slavic mutation
**LDLR**<br>NM_000527.5<br><br>c.662A>G<br>(p.Asp221Gly)<br><br>Not reported in dbSNP<br>VCV000183092.56<br><br>Pathogenic		Northern Poland: 13/68 (19%) [Mickiewicz et al., 2016]; 11/13 carriers were of Kashubian origin	Occasionally identified in various populations				Regional (Kashubian / Polish) mutation
**MAN2B1**<br>NM_000528.4<br><br>c.2248C>T<br>(p.Arg750Trp, also reported as p.Arg749Trp)<br><br>rs80338680	Alpha-mannosidosis<br>#248500<br><br>AR<br><br>1:500000	Poland: 12/20 (60%) [Lipiński et al., 2023]	The most frequent mutation in several European countries [Lipiński et al., 2023]	Russia: 26/10904 (0.0024)	Finns: 29/25036 (0.001158)<br>NFE: 48/128844 (0.0003725)<br>Estonians:14/4666 (0.003)<br>Bulgarians: 2/2670 (0.00075)		Slavic mutation

VCV000001687.52<br><br>Pathogenic							
**MLH1**<br>NM_000249.3<br><br>c.392C>G (p.Ser131*)<br><br>rs63749818<br>VCV000234370.7<br><br>Pathogenic	Lynch syndrome<br><br>#120435<br><br>AD<br><br>Prevalence is variable across Europe			Russia: 0/1600		All carriers share the same haplotype [Hiljadnikova-Bajro et al., 2012]	Regional (Macedonian) mutation
**MLH1**<br>NM_000249.4<br><br>c.244A>G (p.Thr82Ala)<br><br>rs587778998<br>VCV000090116.20<br><br>Likely pathogenic		Macedonia: 3/15 (20%) [Staninova-Stojovska et al., 2019]	Occasionally occurs outside Macedonia	Russia: 0/1600			Regional (Macedonian) mutation
**MLH1**<br>NM_000249.4<br><br>c.677G>T (p.Arg226Leu)<br><br>rs63751711<br>VCV000090319.30<br><br>Likely pathogenic		Slovakia: 2/9 (22%) [Zavodna et al., 2006]<br>Poland: 3/41 (7%) [Kurzawski et al., 2006]<br>Russia: 2/10 (20%) [Yanus et al., 2020]	This mutation is a minor recurrent allele, identified in various populations, however, it is significantly more frequent in Slavic patients	Russia: 0/1600			Slavic mutation
**MSH2**<br>NM_000249.4<br><br>c.2211-2A>C<br><br>rs267608001		Macedonia: 3/5 (60%) [Staninova-Stojovska et al., 2019]	Also detected in Chinese subjects [Liu et al., 2004]	Russia: 0/1600			Regional (Macedonian) mutation

VCV000090929.4<br><br>Likely pathogenic							
**MMAA**<br>NM_172250.3<br><br>c.593_596del (p.Thr198Serfs)<br><br>Also reported as c.590_593delTGAC; c.592_595del<br><br>rs796051993<br>VCV000203815.5<br><br>Pathogenic	Vitamin B12-responsive methylmalonic aciduria, type cblA<br><br>#251100<br><br>AR<br><br><1:1000000<br><br>cblA vitamin B12-responsive subtype is a subset of methylmalonic aciduria, a condition with a prevalence of 1:48000 – 1:61000 (North America); 1:26000 (China)	Poland: 6/8 (75%) [Wesół-Kucharska et al., 2020]	A minor recurrent mutation in some European studies [Dempsey-Nunez et al., 2012]	Russia: 4/10882 (0.0004)	NFE: 10/129132 (0.00008)<br>Bulgarians: 3/2670 (0.00112)		Regional Polish or pan-Slavic (?) mutation
**MUTYH**<br>NM_001128425.1<br><br>c.734G>A (p.Arg245His)<br><br>rs140342925<br>VCV000140877.13<br><br>Pathogenic / Likely pathogenic	MUTYH-associated polyposis<br><br>#608456<br><br>AR<br><br>1:20000 - 1:60000	Macedonia: 4/4 (100%) [Staninova-Stojovska et al., 2019]<br>Russia: 2/18 (11%) [Yanus et al., 2018]	Highly recurrent in Hungary (5/10. 50%) [Papp et al., 2016]; occasionally occurs in Italy (up to 25%) [de Rosa et al., 2009] and Germany (up to 6%) [Morak et al., 2010]	Russia: 11/10874 (0.001)	EAS: 5/19802 (0.00025)<br>NFE: 16/128442 (0.00012)<br>Bulgarians: 3/2572 (0.001166)		Slavic or Hungarian mutation
**MYBPC3**<br>NM_000256.3<br><br>c.2541C>G (p.Tyr847*)<br><br>rs397515974	Hypertrophic cardiomyopathy, 4; dilated cardiomyopathy, type 1MM; left ventricular noncompaction 10	Poland: 4/29 (14%) [Lipari et al., 2020] + 1 more Polish case reported by others<br>Russia: one case [Maslova et al, 2019]	Occasionally identified in various populations, with the highest reported frequency in the US data set (5/194, 2.6%) [Kapplinger et al., 2014]			All carriers share the same haplotype [Lipari et al., 2020]	Regional (Polish) mutation

VCV000042636.19<br><br>Pathogenic / Likely pathogenic	AD / AR (rare)<br><br>1:2500 (all cases of dilated cardiomyopathy; 20-30% are familial, with many underlying genes)<br>1:500-1:2000 (all varieties of hypertrophic cardiomyopathy)						
**MYBPC3**<br>NM_000256.3<br><br>c.3697C>T (p.Gln1233*)<br><br>rs397516037<br>VCV000042735.39<br><br>Pathogenic / Likely pathogenic		Russia: 8/30 (27%) [Chumakova et al., 2023]<br>Czech: 10/43 (23%) [Bonaventura et al., 2019]	Recurrent mutation in Europe. Interestingly, it has not been observed in Polish and Slovak studies [Lipari et al., 2020; Sepp et al., 2022], while it is very frequent in Hungarian patients (12/55, 22%) [Sepp et al., 2022]		NFE: 2/113004 (0.0000177)		Slavic mutation
**MYO7A**<br>NM_000260.4<br><br>c.52C>T (p.Gln18*)<br><br>rs1555051455<br>VCV000504505.5<br><br>Pathogenic	Usher syndrome, type IB<br><br>#276900<br><br>AR<br><br>Usher syndrome type I frequency is 1:100000 – 9:100000	Slovenia: 6/12 (50%) [Bonnet et al., 2016]<br>Russia: 3/14 (21%) [Ivanova et al., 2018]	Occasionally reported in non-Slavic patients; absent in Danish, French, German, Italian and Spanish large data sets [Bonnet et al., 2016]	Russia: 4/10900 (0.0004)			Slavic mutation
**NBN (NBS1)**<br>NM_002485.5<br><br>c.657_661del (p.Lys219fs)<br><br>Also reported as c.657del5<br><br>rs587776650<br>VCV000006940.82<br><br>Pathogenic	Nijmegen breakage syndrome<br><br>#251260<br><br>AR<br><br>Prevalence is unknown<br><br>Low penetrance breast cancer predisposition in heterozygotes?	Czech Republic: 3/67 (4,5%) children with microcephaly [Seeman et al., 2004]	It is the most frequent NBN pathogenic allele in various European populations, however, its frequency is significantly higher in Slavic countries	Carrier frequency is approximately 0.5–1.0% in Ukraine, Poland, Czech Republic, Slovakia, Sorbs in Germany, Bulgaria [Seemanova et al., 2016]<br>MAF in Russia: 35/10756	NFE: 52/128774 (0.0004)<br>Bulgarians: 5/2666 (0.00188)	All carriers share the same haplotype [Rusak et al., 2019]	Slavic mutation

	[Rusak et al., 2019]			(0.0033)			
**NHLRC1**<br>NM_198586.3<br><br>c.1048_1049delGA (p.E350fs)<br><br>Not reported in dbSNP<br>Not reported in ClinVar<br><br>Uncertain significance / Pathogenic	Myoclonic epilepsy of Lafora 2B<br><br>#608072<br><br>AR<br><br>1:1000000 in Europe; NHLRC1–associated: 20% of cases.<br>In Serbians, 100% of cases are associated with NHLRC1 [Kecmanović et al., 2013]	11/20 (55%) alleles in patients of Serbian and Montenegrin origin [Kecmanović et al., 2016]	Not reported outside Serbia			All carriers share the same haplotype [Kecmanović et al., 2013]	Regional (Serbian) mutation
**NHLRC1**<br>NM_198586.3<br><br>Whole gene deletion<br><br>Not reported in dbSNP<br>Not reported in ClinVar<br><br>Uncertain significance / Pathogenic		5/20 (25%) patients of Serbian and Montenegrin origin [Kecmanović et al., 2016]				All carriers share the same haplotype [Kecmanović et al., 2016]	Regional (Serbian) mutation
**NPHS2**<br>NM_014625.4<br><br>c.868G>A (p.Val290Met)<br><br>rs200482683<br>VCV000126418.39<br><br>Pathogenic / Likely pathogenic / Uncertain significance	Nephrotic syndrome, type 2<br><br>#600995<br><br>AR<br><br>Prevalence is unknown	Czech Republic: 6/12 (50%) [Thomasová et al., 2023]<br>Poland: 2/10 (20%) [Bińczak-Kuleta et al., 2014]<br>Poland (Kashubia): 1/40 (2.5%) [Lipska et al., 2013]	Occasionally identified in European pateints, most frequently in Hungary 3/14 (21%) [Kerti et al., 2013]	Russia: 27/10876 (0.0025)	NFE: 31/128504 (0.0002412)<br>Estonians: 5/4834 (0.001034)<br>Bulgarians: 2/2666 (0.0007502)	All carriers share the same haplotype [Thomasová et al., 2023]	Balto-Slavic mutation (?)
**NPHS2**		Poland: 11/40 (28%) [Lipska et	Occasionally identified				Regional (Kashubian

NM_014625.4<br><br>c.1032delT (p.Phe344Leufs)<br><br>Not reported in dbSNP<br>Not reported in ClinVar<br><br>Uncertain significance / Pathogenic		al., 2013]	in Western European data sets [McCarthy et al., 2013]				/ Polish) mutation
**NUP93**<br>NM_014669.5<br><br>c.1772G>T (p.Gly591Val)<br><br>rs145473779<br>VCV000224964.9<br><br>Pathogenic / Likely pathogenic	Nephrotic syndrome, type 12<br><br>#616892<br><br>AR<br>1:200000 - 1:500000 (all combined)	Central and Eastern Europe (Poland, Czech, Germany, Hungary, Russia, Serbia) and Turkey: 31/62 (50%) [Jankowski et al., 2021]	Reported in Germany, Hungary, Turkey [Jankowski et al., 2021]	Russia: 14/8046 (0.0017)	NFE: 38/128796 (0.000295)<br>Bulgarians: 2/2670 (0.0007491)	All carriers share the same haplotype [Jankowski et al., 2021]	Slavic mutation
**PAH**<br>NM_000277.1<br><br>c.1222C>T (p.Arg408Trp)<br><br>rs5030858<br>VCV000000577.11<br><br>Pathogenic	Phenylketonuria (PKU)<br><br>#261600<br><br>AR<br><br>1:15000	Slovenia: 28% alleles [Groselj et al., 2012]<br>Serbia: 19/116 (16%) [Djordjevic et al., 2012]<br>Ukraine: 51.5% [Pampukha et al., 2016]<br>Slovakia: 196/404 (49%) [Polak et al., 2013]<br>Czech Republic: 560/1323 (42%) [Réblová et al., 2013]; 105/152 (55.3%) [Kozak et al., 1995]<br>Poland: 1590/2572 (62%) [Bik-Multanowski et al., 2010]; 90/114 (79%) [Jaruzelska et al., 1993]<br>Russia: 51% [Gundorova et al, 2019]	This mutation is very frequent in Ireland and in Eastern Europe, but it is virtually absent from Southern Europe [Tighe et al., 2003]	Russia: 22/1794 (0.01226) [Yanus et al., 2019]; 99/10896 (0.0091) (Ruseq)	NFE: 222/129172 (0.00172)<br>Estonians: 44/4830 (0.00911)<br>Bulgarians: 5/2668 (0.00187)	A common haplotype in Balto-Slavic carriers and an independent haplotype in Irish carriers [Tighe et al., 2003]	Balto-Slavic mutaton
**PAH**<br>NM_000277.1		Serbia: 36/116 (31%) [Djordjevic et al., 2012]<br>Czech Republic: 25/1323 (2%)	A minor recurrent mutation in various European populations	Russia: 2/10894 (0.0002)	Latino: 9/35440 (0.00025)<br>NFE: 22/129138		Regional (Serbian) mutation

c.143T>C (p.Leu48Ser)<br><br>rs5030841 VCV000000608.91<br><br>Pathogenic		[Réblová et al., 2013]; 4/152 (2.1%) [Kozák et al., 1995]	(e.g. Spain, Italy, Germany) and in Turkey [Couce et al., 2013; Dobrowolski et al., 2010]		(0.00017)<br>Bulgarians: 6/2670 (0.00225)		
**PALB2** NM_024675.4<br><br>c.168_171delTTGT (p.Gln60fs)<br><br>Also reported as c.172_175delTTGT, according to NM_024675.3<br><br>rs180177143 VCV000126623.12<br><br>Pathogenic / Likely pathogenic	Breast cancer susceptibility<br><br>#114480<br><br>AD<br><br>Fanconi anemia, complementation group N<br><br>#610832<br><br>AR	Czech Republic: 4/13 (31%) [Janatova et al., 2013]<br>Poland: 7/23 (30%) [Cybulski et al., 2019]	Occasionally reported in other European populations (ClinVar)	Poland: 4/3380 (0.0019) [Kluska et al., 2017]	ASJ: 1/10368 (0.0001)<br>NFE: 10/129184 (0.00008)<br>Bulgarians: 0/2670	Czech carriers share the same haplotype [Janatova et al., 2013]	Slavic mutation
**PALB2** NM_024675.4<br><br>c.509_510delGA (p.Arg170Ilefs)<br><br>rs515726123 VCV000126757.16<br><br>Pathogenic		Poland: 12/23 (52%) [Cybulski et al., 2019]<br>Czech Republic: 1/13 (8%) [Janatova et al., 2013]<br>Major founder allele in Russia and Belarus [Noskowicz et al., 2014]	Frequent in Germany [Noskowicz et al., 2014]; occasionally reported in various populations (ClinVar)	Poland: 1/2620 (0.0004) [Dansonka-Mieszkowska et al., 2010]; 0/3380 [Kluska et al., 2017]<br>Russia: 1/10928 (0.0001)	OTH: 1/6138 (0.00016)<br>NFE: 8/113732 (0.00007)<br>Bulgarians: 0/2670		Slavic mutation
**PCDH15** NM_001142769.1<br><br>c.1103delT p.Leu368Trpfs*58	Usher syndrome, type IF<br><br>#276900	Slovenia: 4/4 (100%) [Bonnet et al., 2016]	Founder mutation in Hutterites (a religious group, which originated from Switzerland, moved to Moravia, and after			Hutterite carriers share the same haplotype; no data about Slovenian carriers [Chong et	A “transit” mutation, originating either in early Hutterite communities or in Slavic populations of

c.1088delT (p.Leu363fs) according to NM_033056.4<br><br>Also reported as c.1471delT<br><br>rs199469706 VCV000004932.2<br><br>Pathogenic	AR<br><br>Usher syndrome type I frequency is 1:100000 – 9:100000		several hundred years of migrations across Hungary, Balkan Slavic countries, Ukraine and Russia was finally driven by religious prosecutions to North America) [Chong et al., 2012]			al., 2012]	former Austro-Hungarian Empire
**PMS2** NM_000535.3<br><br>c.(803+1_804-1)_(*1_?)del p.(?)<br><br>Not reported in dbSNP Not reported in ClinVar<br><br>Uncertain significance / Pathogenic	Lynch syndrome<br><br>#120435<br><br>AD<br><br>Prevalence is variable across Europe	Macedonia: 3/5 (60%) [Staninova-Stojovska et al., 2019]	Not reported outside Macedonia	Russia: 0/1600			Regional (Macedonian) mutation
**PROP1** NM_006261.4 c.301_302delGA (p.Leu102Cysfs)<br><br>rs193922688 VCV000008098.31<br><br>Pathogenic / Likely pathogenic	Combined pituitary hormone deficiency-2<br><br>#262600<br><br>AR<br><br>1:3000-1:4000 (all genetic types)	Czech Republic: 23/32 (72%) [Obermannova et al., 2011] Poland: 49/56 (88%) [Obermannova et al., 2011] Russia: 11/14 (79%) [Obermannova et al., 2011]	This pan-European mutation is less frequent in non-Slavic and non-Iberic parts of Europe	9/1794 (0.00502) [Yanus et al., 2019]	NFE: 42/128934 (0.00033) Estonians: 6/4766 (0.00126) Bulgarians: 3/2660 (0.00113)	In the majority of European patients, this allele is located within the same haplotype (originated ~101 generations ago in Lithuania). Patients from the Iberian Peninsula have different haplotype (originated ~23 generations ago). Both haplotypes have been transmitted to Latin American	Balto-Slavic founder mutation (with identical South American-Iberic variant of possibly independent origin)

						patients [Dusatkova et al., 2016]	
**PROP1** NM_006261.4<br><br>c.150del (p.Arg53Aspfs)<br><br>rs587776683 VCV000008102.5<br><br>Pathogenic		Czech Republic: 8/32 (25%) [Obermannova et al., 2011]<br>Russia: 1/14 (7%) [Obermannova et al., 2011]	This pan-European mutation is less frequent in non-Slavic regions of Europe		NFE: 25/123778 (0.0002)<br>OTH: 13/31626 (0.00041)<br>Bulgarians: 1/2552 (0.00039)	PROP1 c.150delA allele is linked to a haplotype, which emrged ~ 44 generations ago in Belarus [Dusatkova et al., 2016]	Slavic mutation
**PROP1** NM_006261.4<br><br>c.150_151del (p.Gly52fs)<br><br>rs587776681 VCV000008097.1<br><br>Pathogenic		Poland: 6/56 (11%) [Obermannova et al., 2011]	Never reported outside Slavic populations				Regional (Polish) Slavic mutation
**PRNP** NM_000311.5<br><br>c.598G>A (p.Glu200Lys)<br><br>rs28933385 VCV000013398.38<br><br>Pathogenic	Creutzfeldt-Jakob disease (familial)	Slovakia: 23 Slovak and 1 Polish kindred with E200K mutation [Lee et al., 1999]<br>Poland: 5/8 (63%) [Zimowski et al., 2012]	This mutation occurs in more than 70% of families with CJD worldwide. Prevalence is very high in Chile, Libyan and Tunisian Jews.	Russia: 0/1600	Latino: 1/34592 (0.00003)<br>NFE: 0	Slavic E200K allele has an independent origin, which is distinct from Lybian Jewish, Sephardic Jewish, Spanish and other Mediterranean instances of this allele; the haplotypes in families from Germany, Sicily, Austria, and Japan are also different [Lee et al., 1999]	Regional (Slovakian, Polish ?) mutation<br><br>It is also a hotspot mutation: identical substitutions of independent origin were reported in other parts of the world
**RECQL** NM_002907.4<br><br>c.1667_1667+3del	Low penetrance breast cancer predisposition [Cybulski et al., 2023]	Poland: 2/4 (50%) [Cybulski et al., 2015]		Russia: 3/8028 (0.0004)	NFE: 66/127824 (0.0005163)<br>Swedish: 28/26034 (0.001076)<br>Estonians: 4/4836		Scandinavian (?), Central European (?) or Slavic (?) mutation

rs564485792<br>VCV000852047.18<br><br>Likely pathogenic / Uncertain significance	Not reported in OMIM<br><br>AD?<br><br>RECON progeroid syndrome<br><br>#620370<br><br>AR<br><br>Extremely rare				(0.0008271)<br>Bulgarians 2/2660 (0.0007519)		
**RAG2** NM_000536.3<br><br>c.1300T>C (p.Tyr434His)<br><br>Not reported in dbSNP<br>Not reported in ClinVar<br><br>Uncertain significance / Likely pathogenic	Severe combined immunodeficiency (usually incl. Omenn syndrome)<br><br>#233650 #603554 #601457<br><br>AR<br><br>1:100000 – 9:100000 (RAG1/2 deficiency)	Ukrainian, Russian, and Belarusian patients: 3/4 (75%) [Sharapova et al., 2016]<br>Russia: 5/10 (50%) of all pathogenic alleles [Sharapova et al., 2020]					Regional (Russian?) Slavic mutation
**RAG2** NM_000536.3<br><br>c.1357T>C (p.Trp453Arg)<br><br>rs1564995627<br>VCV000496630.1<br><br>Likely pathogenic		Poland: 6/16 (38%) [Sharapova et al., 2020]	Occasionally reported in non-Slavic patients [Tirosh et al., 2019]				Regional (Polish?) Slavic mutation

**RUNX2 (CBFA1)**<br>NM_001024630.4<br><br>c.577C>T (p.Arg193*)<br><br>rs1582105417<br>VCV000829868.1<br><br>Pathogenic	Cleidocranial dysplasia<br><br>#119600<br><br>AD<br><br>1:1000000 – 9:1000000	Poland: 3/4 (75%) [Kisiel et al., 2006]	Rarely identified in non-Slavic populations [Kisiel et al., 2006]				Regional (Polish) mutation
**SCO2**<br>NM_005138.2<br><br>c.418G>A (p.Glu140Lys)<br><br>Also reported as NCAPH2 (NM_152299.4) c.*619C>T; c. G1541A<br><br>rs74315511<br>VCV000005681.32<br><br>Pathogenic	Mitochondrial complex IV deficiency nuclear type 2 / Leigh syndrome etc.<br><br>#604377<br><br>AR<br><br>Prevalence is unknown	Poland: 31/36 (86%) [Pronicki et al., 2010]<br>Poland, Czech Republic, and Slovakia: 10/12 (83%) [Böhm et al., 2006]	Occasionally reported outside Slavic populations (ClinVar)	Russia: 2/1794 (0.00111) [Yanus et al., 2019]; 18/10844 (0.0017) (Ruseq)	NFE: 23/128958 (0.00018)<br>Estonians: 4/4826 (0.00083)<br>Bulgarians: 1/2656 (0.00038)		Balto-Slavic mutation
**SDHD**<br>NM_003002.4<br><br>c.33C>A (p.Cys11X)<br><br>rs104894309<br>VCV000006915.7<br><br>Pathogenic	Hereditary paraganglioma-pheochromocytoma syndrome<br><br>#171300<br><br>AD (with paternal imprinting)<br><br>Unknown (especially considering incomplete		Rarely encountered outside Poland [Peczkowska et al., 2008]		SAS: 1/30616 (0.00003)	All carriers share the same haplotype [Peczkowska et al., 2008]	Regional (Polish) Slavic founder mutation
**SDHD**<br>NM_003002.4<br><br>c.305A>G (p.His102Arg)		Russia: 3/9 (33%) [Shulskaya et al., 2018]; 11/34 (32%) [Snezhkina et al., 2023]	Rarely encountered outside Russia	Russia: Carrier frequency: 6/373 (1.6%) [Snezhkina	Southern Europeans: 1/113762 (0.000008790)		Regional (Russian) Slavic mutation

rs104894302<br>VCV000656860.6<br><br>Pathogenic / Likely pathogenic	penetrance)<br>1:50000 - 1:75000			et al., 2023]<br>MAF: 8/10870 (0.0007) (Ruseq)			
**SERPINC1**<br>NM_000488.4<br><br>c.1157T>C (p.Ile386Thr)<br><br>rs1449772752<br>VCV000863657.6<br><br>Pathogenic / Likely pathogenic	Thrombophilia 7 due to antithrombin III deficiency<br><br>#613118<br><br>AD (type I, hypomorphic)<br><br>0.02% to 0.2% [Weronska et al., 2023]	Poland: 10/62 (16%) [Weronska et al., 2023]	Occasionally identified in various Central European (German, Hungarian, Czech, French) populations [Weronska et al., 2023]		NFE: 1/1137441 (0.000008792)	All carriers share the same haplotype (regional clustering in the region of Malopolska is observed) [Weronska et al., 2023]	Regional mutation (Poland, Maloposka)
**SLC7A9**<br>NM_014270.5<br><br>c.313G>A (p.Gly105Arg)<br><br>rs121908480<br>VCV000005781.5<br><br>Pathogenic	Cystinuria<br><br>#220100<br><br>AR / AD with reduced penetrance<br><br>1:10000 – 5:10000	Czech Republic: 3/11 (27%) [Skopková et al., 2005]<br>Macedonia: 5/15 (33%)<br>Serbia: 2/18 (11%)<br>Croatia: 2/4 (50%) [Popovska-Jankovic et al., 2013]	Also frequent in Albania, Turkey and certain Southern European countries [Popovska-Jankovic et al., 2013]. This mutation is a minor recurrent allele throughout the rest of Europe	Russia: 5/10920 (0.0005)	NFE: 70/128832 (0.00054)<br>Bulgarians: 5/2664 (0.00188)		Southern European / Southern Slavic mutation
**SLC7A9**<br>NM_014270.5<br><br>c.955G>A (p.Gly319Arg)<br><br>Not reported in dbSNP<br>VCV000836401.1<br><br>Likely pathogenic		Czech Republic: 3/11 (27%) [Skopková et al., 2005]	One report from China [Shen et al., 2017]				Regional (Czech) mutation
**SLC26A3**<br>NM_000111.2	Diarrhea 1, secretory chloride,	Poland: 16/34 (47%) [Höglund et al., 1998]	Extremely rare outside Poland; there are case	Russia: 10/10710	NFE: 17/128892 (0.00013)	All carriers shaee the same	Regional (Polish) mutation

c.2024_2026dup (p.Ile675dup)<br><br>Also reported as c.2025_2026insATC<br><br>rs121913031 VCV000055988.3<br><br>Pathogenic	congenital<br><br>#214700<br><br>AR<br><br>Frequency is unknown		reports from Hungary and Turkey [Özbay Hoşnut et al., 2010; Dávid et al., 2019]	(0.0009)	Estonians: 2/4828 (0.00041)<br>Bulgarians: 1/2668 (0.00037)	haplotype; increased prevalence in Southern Poland [Höglund et al., 1998]	
**SOD1**<br>NM_000454.5<br><br>c.10A>G, (p.Lys4Glu; also reported as K3E)<br><br>No rs number VCV002138372.1<br><br>Pathogenic	Amyotrophic lateral sclerosis<br><br>#105400<br><br>AD<br><br>1:500000 (hereditary forms combined)	Poland: 8 unrelated cases [Kuźma-Kozakiewicz et al., 2013]	Also reported in Japan [Kuźma-Kozakiewicz et al., 2013]			Several haplotypes were identified in Polish carriers, all distinct from Japanese [Kuźma-Kozakiewicz et al., 2013]	Regional (Polish) mutation
**SOD1**<br>NM_000454.5<br><br>c.434T>C (p.Leu144Phe; also reported as p.Leu144Ser)<br><br>rs1482760341 VCV000586637.21<br><br>Pathogenic		Serbia: 21/27 (78%) unrelated cases [Marjanović et al., 2017]					Regional (Serbian) mutation
**SOD1**<br>NM_000454.5<br><br>c.434T>C (p.Leu145Ser; also reported as		Poland: 7 unrelated cases [Kuźma-Kozakiewicz et al., 2020]	Also reported in Brazil, Iran and United States [Kuźma-Kozakiewicz et al., 2020]			Polish and US carriers, and probably even Iranian ones, share the same haplotype	Regional (Polish) mutation

p.Leu144Ser)<br><br>rs121912446<br>VCV000014768.25<br><br>Pathogenic						[Kuźma-Kozakiewicz et al., 2020]	
**SLC37A4**<br>NM_001164277.2<br><br>c.81T>A<br>(p.Asn27Lys)<br><br>rs193302889<br>VCV000068290.12<br><br>Pathogenic / Likely pathogenic / Uncertain significance	Glycogen storage disease (GSD), type 1b<br><br>#232220<br><br>AR<br><br>1:33000 - 1:400000<br><br>GSD type Ib is infrequent variant of this disease, however, it is prevalent in Serbia [Skakic et al., 2018]	Serbia: 25/54 (46%) [Skakic et al., 2018]	Occasionally reported in European patients		NFE: 2/112944 (0.00002)<br>Bulgarians: 1/2664 (0.0004)		Regional (Serbian) Slavic mutation
**SLC37A4**<br>NM_001164277.2<br><br>c.1042_1043delCT<br>(p.Leu348Valfs*53)<br><br>rs80356491<br>VCV000006926.46<br><br>Pathogenic		Serbia: 21/54 (39%) [Skakic et al., 2018]	One of the two most frequent mutations reported in Europe, but its frequency is particularly high in Serbian patients	Russia: 8/10798 (0.0007)	ASJ: 3/10286 (0.00029)<br>NFE: 33/126790 (0.00026)<br>Estonians: 7/4828 (0.00145)<br>Bulgarians: 2/2640 (0.00076)		Balto-Slavic mutation
**SLURP1**<br>NM_020427.3<br><br>c.82delT<br>(p.Cys28Alafs)<br><br>rs587776601<br>VCV000004599.7<br><br>Pathogenic	Meleda disease (mal de Meleda; Mljet disease, keratosis palmoplantaris and transgradiens of Siemens)<br><br>#248300<br><br>AR	Croatia: 8/14 (57%) [Fischer et al., 2001]	A major founder mutation in Algeria and Tunisia; several cases were also identified in Kurdish population, Italy and Scotland [Bchetnia et al., 2013]	Russia: 2/10900 (0.0002)	OTH: 1/7190 (0.00014)<br>NFE: 8/128026 (0.00006)<br>OTH: 4/32506 (0.00012)<br>Bulgarians: 0/2640	Croatian and Algerian families share the same haplotype [Fischer et al., 2001]	Regional (Croatian / Algerian)
**SLURP1**		Croatia: 6/14 (43%) [Fischer et	Also reported in single	Russia:	SAS: 1/30600	Croatian families	Regional (Croatian)

NM_020427.3<br><br>c. 286C>T (p. Arg96Ter)<br><br>rs121908317 VCV000004601.1<br><br>Pathogenic	1:100000 – 9:100000	al., 2001]	families from Korea, Turkey and Pakistani emigrants in USA (ClinVar)	0/1600	(0.00003)<br>NFE: 1/112222 (<0.00001)<br>Bulgarians: 1/2642 (0.00038)	share the same haplotype [Fischer et al., 2001]	mutation
**SURF1**<br>NM_003172.4<br><br>c.841_842delCT (p.Ser282Cysfs)<br><br>Also reported as 845-846delCT<br><br>rs782316919 VCV000012770.46<br><br>Pathogenic	Mitochondrial complex IV deficiency, nuclear type 1 / Leigh syndrome etc.<br><br>#220110<br><br>AR<br><br><1:1000000	Poland, Czech Republic, and Slovakia: 49/63 (78%) [Böhm et al., 2006]<br>Poland: 45/58 (78%) [Piekutowska-Abramczuk et al., 2009]<br>Russia: 35/54 (65%) [Tsygankova et al., 2010]	Recurrent minor mutation in European patients 4/18 (22%) [Tiranti et al., 1998]	Carrier frequency in Poland: 1:357 [Pronicka and Sykut-Cegielska, 2008]<br>MAF in Russia: 15/10906 (0.0014)	NFE: 19/128694 (0.00015)<br>Estonians: 3/4828 (0.00062)<br>Bulgarians: 0/ 2666		Pan-European mutation of Balto-Slavic origin
**TGFBI**<br>NM_000358.2<br><br>c.1673T>C (Leu558Pro)<br><br>Not reported in dbSNP<br>Not reported in ClinVar<br><br>Uncertain significance / Pathogenic	Lattice corneal dystrophy, type IV (atypical)<br><br>This specific type is not reported in OMIM<br><br>AD<br><br>Prevalence is unknown	Ukraine: 4 families [Pampukha et al., 2009]	The disease is relatively common in Spain [Campos-Mollo et al., 2019]			Ukrainian carriers share the same haplotype [Pampukha et al., 2009]	Regional (Ukrainian) mutation
**TGM5**<br>NM_201631.4<br><br>c.337G>T (p.Gly113Cys)	Acral peeling skin syndrome<br><br>#609796	Czech Republic: 74/80 (93%) [Kopečková et al., 2016]<br>Poland: 75/104 (72%) [Szczecinska et al., 2014]	This is the most common mutation in European patients [van der Velden et al., 2012]; however, its prevalence is higher in	Russia: 22/1794 (0.01226) [Yanus et al., 2019];	NFE: 462/128926 (0.0036)<br>Bulgarians: 15/2670 (0.00562)	All carriers share the same haplotype [Szczecinska et al., 2014]	Pan-European mutation of Slavic origin

rs112292549<br>VCV000006039.35<br><br>Pathogenic	AR<br><br><1:1000000 (underrecognized condition)		populations of Slavic descent	67/10868 (0.0062) (Ruseq)			
**TPM1**<br>NM_001018005.2<br><br>c.629A>G (p.Gln210Arg)<br><br>rs777139450<br>VCV000845983.9<br><br>Uncertain significance / Likely pathogenic	Cardiomyopathy, dilated, 1Y<br>Cardiomyopathy, hypertrophic, 3<br>Left ventricular noncompaction, 9<br><br>#611878 #115196 #611878<br><br>AD<br><br>1:2500 (all cases of dilated cardiomyopathy)<br>1:500-1:2000 (all varieties of hypertrophic cardiomyopathy)	Russia: 3/6 (50%) [Chumakova et al., 2023]			NFE: 1/113632 (0.0000088)		Regional (Russian) mutation
**TPO**<br>NM_001206744.2<br><br>c.1430_1450del (p.Ala477_Asn483del)<br><br>Also reported as 1519_1539del<br><br>Not reported in dbSNP<br>VCV000930340.1<br><br>Uncertain significance / Pathogenic	Familial thyroid dyshormonogenesis 2A<br><br>#274500<br><br>AR<br><br>1: 100000 – 9:100000 (all forms)	Slovenia, Bosnia, and Slovakia: 4/24 (16%) [Avbelj et al., 2007]	Not reported in non-Slavic populations				Regional (Southern Slavic) mutation
**TRIM32**	Muscular dystrophy, limb-	Serbia: 4/4 (100%) in Serbs and Bosnian Serbs [Johnson et al.,	The most frequent mutation in Hutterites (a religious	Russia: 1/10936	NFE: 2/113680 (0.00002)		Regional (Serbian) mutation

NM_012210.4<br><br>c.1459G>A (p.Asp487Asn)<br><br>rs111033570 VCV000007350.8<br><br>Pathogenic	girdle (LGMD), autosomal recessive 8<br><br>#254110<br><br>AR<br><br>1:14500 - 1:123000 (all LGMDs combined)	2019]	group, which originated from Switzerland, moved to Moravia, and, after several hundred years of migrations across Hungary, Balkan Slavic countries, Ukraine and Russia, was finally driven by religious prosecutions to North America [Chong et al., 2012; Johnson et al., 2019]	(0.0001)			
**TTN** NM_001267550<br><br>c.107635C>T, p.(Gln35879Ter)<br><br>rs757082154 VCV000202529.34<br><br>Pathogenic / Likely pathogenic / Uncertain significance	Muscular dystrophy, limb-girdle (LGMD), autosomal recessive 10 (adult onset distal myopathy with predominant lower limb involvement)<br><br>#608807<br><br>AR<br><br>1:14500 - 1:123000 (all LGMDs combined)	Serbia: 17/28 (61%) [Peric et al., 2017]	Occasionally reported in multiple populations as singleton cases (apparently, a hotspot)		AMR: 1/34510 (0.00003)<br>NFE: 2/112712 (0.00002)	All Serbian carriers share the same haplotype [Peric et al., 2017]	Regional (Serbian) mutation
**TTR** NM_000371.3<br><br>c.325G>C (p.Glu109Gln)<br><br>Also reported as Glu89Gln<br><br>rs121918082 VCV000013442.25<br><br>Pathogenic	Hereditary transthyretin amyloidosis<br><br>#105210<br><br>AD<br><br>Prevalence unknown	Bulgaria: 267/340 (79%) [Chamova et al., 2022]	Frequent in certain regions of Italy (Sicily), Turkey, North Macedonia	Russia: 0/1600		All carriers share the same haplotype [Kirov et al., 2019]	Mediterranean mutation, with the highest known prevalence in Bulgaria

**USH2A**<br>NM_206933.4<br><br>c.11864G>A<br>(p.Trp3955*)<br><br>rs111033364<br>VCV000002357.14<br><br>Pathogenic	Usher syndrome, type IIA<br><br>#276901<br><br>AR<br><br>Usher syndrome type II frequency is 1:100000 – 9:100000	Slovenia: 33/40 (83%) [Bonnet et al., 2016]<br>Czech Republic: >53% of all pathogenic alleles [Liskova et al., 2016]<br>Russia: 6/20 (30%) [Ivanova et al., 2018]	One of major Central-Southern European mutations (frequent in France, Germany, Italy); however, it is not the most frequent pathogenic allele in non-Slavic populations [Bonnet et al., 2016]	Russia: 27/10926 (0.0025)	NFE: 32/128914 (0.00025)<br>Estonians: 7/4828 (0.001450)<br>Bulgarians: 2/2668 (0.0007496)	4 different haplotypes [Zupan et al., 2019]	Central / Eastern European founder mutation of Slavic origin?<br><br>The frequency of this pan-European mutation is higher in (Balto)Slavic countries than in NFE<br><br>This is a hotspot mutation, with at least one haplotype spread across Slavic populations as a founder allele
**USH2A**<br>NM_206933.3<br><br>c.2610C>A<br>(p.Cys870*)<br><br>rs767078782<br>VCV000557167.3<br><br>Pathogenic		Slovenia: 7/40 (18%) [Bonnet et al., 2016]	Occasionally reported in non-Slavic patients [Le Quesne Stabej et al., 2012; Bonnet et al., 2016]	Russia: 1/10922 (0.0001)	NFE: 4/128532 (0.00003)<br>Swedish: 2/26126 (0.00007)<br>Bulgarians: 0/2668		Regional (Slovenian) mutation
**VHL**<br>NM_000551.3<br><br>c.571C>G<br>(His191Asp)<br><br>rs28940301<br>VCV000002235.9<br><br>Likely pathogenic	Familial erythrocytosis<br><br>#263400<br><br>AR<br><br>Prevalence unknown	Croatia: 4/4 (100%) (patients were remotely related, but not via supposed carriers) [Tomasic et al., 2013]	Another VHL allele is associated with Chuvash type polycythemia; one instance of c.571C>G homozygote was observed in a patient with unreported ancestry [Camps et al., 2016]	Russia: 0/1600			Regional (Croatian) mutation
**VPS16**<br>NM_022575.4<br><br>c.559C>T<br>(p.Arg187*)	Early-onset generalized dystonia (Dystonia 30)<br><br>#619291	Czech Republic: 3 unrelated individuals (Slovaks) [Ostrozovicova et al., 2021]	1 second-generation UK immigrant of uncertain, possibly Eastern European origin [Ostrozovicova et al., 2021]			All carriers share the same haplotype [Ostrozovicova et al., 2021]	Regional (Slovak) mutation

Not reported in dbSNP<br>VCV001065413.4<br><br>Pathogenic / Uncertain significance	AD<br><br>1:200000 – 1:330000 in Europe						
**WDR35**<br>NM_020779.4<br><br>c.1889T>G (p.Leu630Ter)<br><br>Also reported as c.1922T>G p.Leu641Ter<br><br>rs199952377<br>VCV000065619.23<br><br>Pathogenic / Likely pathogenic	Cranioectodermal dysplasia, also known as Sensenbrenner syndrome<br><br>#613610<br><br>AR<br><br><1:1000000	Poland: 4/12 (25%) [Walczak-Sztulpa et al., 2020]	Occasionally reported in other European and US data sets	Russia: 26/10560 (0.0025)	NFE: 42/108456 (0.00039)<br>Estonians: 14/4824 (0.0029)<br>Bulgarians: 2/2496 (0.0008)		Balto-Slavic mutation
**WDR35**<br>NM_020779.4<br><br>c.2489A>T (p.Asp830Val)<br><br>Also reported as c.2522A>T p.(Asp841Val)<br><br>rs1553316926<br>VCV000446645.2<br><br>Pathogenic / Likely pathogenic		Poland: 6/12 (50%) [Walczak-Sztulpa et al., 2020]	Reported in ClinVar from a diagnostic laboratories in USA	Russia: 1/10876 (0.0001)			Regional (Polish) Slavic mutation
**ZMYND10**<br>NM_015896.2<br><br>c.367delC	Ciliary dyskinesia, primary, 22<br><br>#615444	Poland: 4/4 (100%) [Kurkowiak et al., 2016]				All carriers share the same haplotype [Kurkowiak et al.,	Regional? (Polish) mutation

(p.His123Thrfs)<br><br>Not reported in dbSNP<br>Not reported in ClinVar<br><br>Uncertain significance / Pathogenic	AR<br><br>All primary ciliary dyskinesias combined: 1:15000 – 1:30000					2016]	

Note: A given allele can be categorized as having Slavic origin if:

1) its origin is proven by haplotyping, or
2) the mutation prevalence in patients belonging to one or several Slavic countries is higher than that in patients from other world populations, according to available publications and/or databases, or
3) the mutation prevalence in healthy population of one or several Slavic countries is higher than that in other world populations (gnomAD database)

Abbreviations: AD: autosomal dominant; AR: autosomal recessive; AFR: African; ASJ: Ashkenazi Jews; EAS: East Asians; MAF: minor allele frequency; NFE: non-Finnish Europeans; OTH: individuals who did not unambiguously cluster with the major populations in a principal component analysis; SAS: South Asians; XLD – X-linked dominant; XLR: X-linked recessive

** Data are taken from the cited literature of the Ruseq database (http://ruseq.ru/)

References to Supplementary Table S1

Abdollahpour H, Alawi M, Kortüm F, Beckstette M, Seemanova E, Komárek V, Rosenberger G, Kutsche K. An AP4B1 frameshift mutation in siblings with intellectual disability and spastic tetraplegia further delineates the AP-4 deficiency syndrome. Eur J Hum Genet. 2015 Feb;23(2):256-9. doi: 10.1038/ejhg.2014.73. Epub 2014 Apr 30.

Ammar N, Nelis E, Merlini L, Barisić N, Amouri R, Ceuterick C, Martin JJ, Timmerman V, Hentati F, De Jonghe P. Identification of novel GDAP1 mutations causing autosomal recessive Charcot-Marie-Tooth disease. Neuromuscul Disord. 2003 Nov;13(9):720-8. doi: 10.1016/s0960-8966(03)00093-2.

Avbelj M, Tahirovic H, Debeljak M, Kusekova M, Toromanovic A, Krzisnik C, Battelino T. High prevalence of thyroid peroxidase gene mutations in patients with thyroid dyshormonogenesis. Eur J Endocrinol. 2007 May;156(5):511-9. doi: 10.1530/EJE-07-0037.

Balabas A, Skasko E, Nowakowska D, Niwinska A, Blecharz P. Novel germline mutations in BRCA2 gene among breast and breast-ovarian cancer families from Poland. Fam Cancer. 2010 Sep;9(3):267-74. doi: 10.1007/s10689-010-9338-5.

Barbitoff YA, Skitchenko RK, Poleshchuk OI, Shikov AE, Serebryakova EA, Nasykhova YA, Polev DE, Shuvalova AR, Shcherbakova IV, Fedyakov MA, Glotov OS, Glotov AS, Predeus AV. Whole-exome sequencing provides insights into monogenic disease prevalence in Northwest Russia. Mol Genet Genomic Med. 2019 Nov;7(11):e964. doi: 10.1002/mgg3.964. Epub 2019 Sep 3.

Barvinska O, Olkhovych N, Gorovenko N. High Prevalence of c.1528G>C Rearrangement in Patients with Long Chain 3-Hydroxyacyl-CoA Dehydrogenase Deficiency from Ukraine. Cytol. Genet. 52, 198–203 (2018). https://doi.org/10.3103/S0095452718030027

Battelino S, Repič Lampret B, Zargi M, Podkrajšek KT. Novel connexin 30 and connexin 26 mutational spectrum in patients with progressive sensorineural hearing loss. J Laryngol Otol. 2012 Aug;126(8):763-9. doi: 10.1017/S0022215112001119. Epub 2012 Jun 15.

Bchetnia M, Laroussi N, Youssef M, Charfeddine C, Ben Brick AS, Boubaker MS, Mokni M, Abdelhak S, Zili J, Benmously R. Particular Mal de Meleda phenotypes in Tunisia and mutations founder effect in the Mediterranean region. Biomed Res Int. 2013;2013:206803. doi: 10.1155/2013/206803. Epub 2013 Sep 4.

Berginc G, Bracko M, Ravnik-Glavac M, Glavac D. Screening for germline mutations of MLH1, MSH2, MSH6 and PMS2 genes in Slovenian colorectal cancer patients: implications for a population specific detection strategy of Lynch syndrome. Fam Cancer. 2009;8(4):421-9. doi: 10.1007/s10689-009-9258-4. Epub 2009 Jun 13.

Bik-Multanowski M, Kaluzny L, Mozrzymas R, Oltarzewski M, Starostecka E, Lange A, Didycz B, Gizewska M, Ulewicz-Filipowicz J, Chrobot A, Mikoluc B, Szymczakiewicz-Multanowska A, Cichy W, Pietrzyk JJ. Molecular genetics of PKU in Poland and potential impact of mutations on BH4 responsiveness. Acta Biochim Pol. 2013;60(4):613-6. Epub 2013 Dec 17.

Bińczak-Kuleta A, Rubik J, Litwin M, Ryder M, Lewandowska K, Taryma-Leśniak O, Clark JS, Grenda R, Ciechanowicz A. Retrospective mutational analysis of NPHS1, NPHS2, WT1 and LAMB2 in children with steroid-resistant focal segmental glomerulosclerosis - a single-centre experience. Bosn J Basic Med Sci. 2014 May;14(2):89-93. doi: 10.17305/bjbms.2014.2270.

Blahakova I, Makaturova E, Kotrbova L, Soukupova M, Lastuvkova J, Kozak L. Molecular screening of Smith-Lemli-Opitz syndrome in pregnant women from the Czech Republic. J Inherit Metab Dis. 2007 Nov;30(6):964-9. doi: 10.1007/s10545-007-0710-z. Epub 2007 Nov 12.

Blazina Š, Debeljak M, Košnik M, Simčič S, Stopinšek S, Markelj G, Toplak N, Kopač P, Zakotnik B, Pokorn M, Avčin T. Functional Complement Analysis Can Predict Genetic Testing Results and Long-Term Outcome in Patients With Complement Deficiencies. Front Immunol. 2018 Mar 21;9:500. doi: 10.3389/fimmu.2018.00500. eCollection 2018.

Bogdanova N, Cybulski C, Bermisheva M, Datsyuk I, Yamini P, Hillemanns P, Antonenkova NN, Khusnutdinova E, Lubinski J, Dörk T. A nonsense mutation (E1978X) in the ATM gene is associated with breast cancer. Breast Cancer Res Treat. 2009 Nov;118(1):207-11. doi: 10.1007/s10549-008-0189-9. Epub 2008 Sep 21. PMID: 18807267

Bonaventura J, Norambuena P, Tomašov P, Jindrová D, Šedivá H, Macek M Jr, Veselka J. The utility of the Mayo Score for predicting the yield of genetic testing in patients with hypertrophic cardiomyopathy. Arch Med Sci. 2019 May;15(3):641-649. doi: 10.5114/aoms.2018.78767. Epub 2018 Oct 8.

Bonnet C, Riahi Z, Chantot-Bastaraud S, Smagghe L, Letexier M, Marcaillou C, Lefèvre GM, Hardelin JP, El-Amraoui A, Singh-Estivalet A, Mohand-Saïd S, Kohl S, Kurtenbach A, Sliesoraityte I, Zobor D, Gherbi S, Testa F, Simonelli F, Banfi S, Fakin A, Glavač D, Jarc-Vidmar M, Zupan A, Battelino S, Martorell Sampol L, Claveria MA, Catala Mora J, Dad S, Møller LB, Rodriguez Jorge J, Hawlina M, Auricchio A, Sahel JA, Marlin S, Zrenner E, Audo I, Petit C. An innovative strategy for the molecular diagnosis of Usher syndrome identifies causal biallelic mutations in 93% of European patients. Eur J Hum Genet. 2016 Dec;24(12):1730-1738. doi: 10.1038/ejhg.2016.99. Epub 2016 Jul 27.

Borowiec M, Antosik K, Fendler W, Deja G, Jarosz-Chobot P, Mysliwiec M, Zmyslowska A, Malecki M, Szadkowska A, Mlynarski W. Novel glucokinase mutations in patients with monogenic diabetes - clinical outline of GCK-MD and potential for founder effect in Slavic population. Clin Genet. 2012 Mar;81(3):278-83. doi: 10.1111/j.1399-0004.2011.01656.x. Epub 2011 Mar 18.

Borská R, Pinková B, Réblová K, Bučková H, Kopečková L, Němečková J, Puchmajerová A, Malíková M, Hermanová M, Fajkusová L. Inherited ichthyoses: molecular causes of the disease in Czech patients. Orphanet J Rare Dis. 2019 May 2;14(1):92. doi: 10.1186/s13023-019-1076-7.

Bourbon M, Alves AC, Medeiros AM, Silva S, Soutar AK; Investigators of Portuguese FH Study. Familial hypercholesterolaemia in Portugal. Atherosclerosis. 2008 Feb;196(2):633-42. doi: 10.1016/j.atherosclerosis.2007.07.019. Epub 2007 Aug 31.

Boutron A, Acquaviva C, Vianey-Saban C, de Lonlay P, de Baulny HO, Guffon N, Dobbelaere D, Feillet F, Labarthe F, Lamireau D, Cano A, de Villemeur TB, Munnich A, Saudubray JM, Rabier D, Rigal O, Brivet M. Comprehensive cDNA study and quantitative analysis of mutant HADHA and HADHB transcripts in a French cohort of 52 patients with mitochondrial trifunctional protein deficiency. Mol Genet Metab. 2011 Aug;103(4):341-8. doi: 10.1016/j.ymgme.2011.04.006. Epub 2011 Apr 19.

Brozek I, Cybulska C, Ratajska M, Piatkowska M, Kluska A, Balabas A, Dabrowska M, Nowakowska D, Niwinska A, Pamula-Pilat J, Tecza K, Pekala W, Rembowska J, Nowicka K, Mosor M, Januszkiewicz-Lewandowska D, Rachtan J, Grzybowska E, Nowak J, Steffen J, Limon J. Prevalence of the most frequent BRCA1 mutations in Polish population. J Appl Genet. 2011 Aug;52(3):325-30. doi: 10.1007/s13353-011-0040-6. Epub 2011 Apr 19.

Bruni F, Di Meo I, Bellacchio E, Webb BD, McFarland R, Chrzanowska-Lightowlers ZMA, He L, Skorupa E, Moroni I, Ardissone A, Walczak A, Tyynismaa H, Isohanni P, Mandel H, Prokisch H, Haack T, Bonnen PE, Enrico B, Pronicka E, Ghezzi D, Taylor RW, Diodato D. Clinical, biochemical, and genetic features associated with VARS2-related mitochondrial disease. Hum Mutat. 2018 Apr;39(4):563-578. doi: 10.1002/humu.23398. Epub 2018 Feb 7.

Bučková H, Nosková H, Borská R, Réblová K, Pinková B, Zapletalová E, Kopečková L, Horký O, Němečková J, Gaillyová R, Nagy Z, Veselý K, Hermanová M, Stehlíková K, Fajkusová L. Autosomal recessive congenital ichthyoses in the Czech Republic. Br J Dermatol. 2016 Feb;174(2):405-7. doi: 10.1111/bjd.13918. Epub 2015 Nov 14.

Campos-Mollo E, Varela-Conde Y, Arriola-Villalobos P, Cabrera-Beyrouti R, Benítez-Del-Castillo JM, Maldonado MJ, Escribano J. Transforming growth factor beta-induced p.(L558P) variant is associated with autosomal dominant lattice corneal dystrophy type IV in a large cohort of Spanish patients. Clin Exp Ophthalmol. 2019 Sep;47(7):871-880. doi: 10.1111/ceo.13532. Epub 2019 May 22.

Camps C, Petousi N, Bento C, Cario H, Copley RR, McMullin MF, van Wijk R, Ratcliffe PJ, Robbins PA, Taylor JC; WGS500 Consortium. Gene panel sequencing improves the diagnostic work-up of patients with idiopathic erythrocytosis and identifies new mutations. Haematologica. 2016 Nov;101(11):1306-1318. doi: 10.3324/haematol.2016.144063. Epub 2016 Sep 20.

Canki-Klain N, Milic A, Kovac B, Trlaja A, Grgicevic D, Zurak N, Fardeau M, Leturcq F, Kaplan JC, Urtizberea JA, Politano L, Piluso G, Feingold J. Prevalence of the 550delA mutation in calpainopathy (LGMD 2A) in Croatia. Am J Med Genet A. 2004 Mar 1;125A(2):152-6. doi: 10.1002/ajmg.a.20408.

Chamova T, Gospodinova M, Asenov O, Todorov T, Pavlova Z, Kirov A, Cherninkova S, Kastreva K, Taneva A, Blagoeva S, Zhelyazkova S, Antimov P, Chobanov K, Todorova A, Tournev I. Seven Years of Selective Genetic Screening Program and Follow-Up of Asymptomatic Carriers With Hereditary Transthyretin Amyloidosis in Bulgaria. Front Neurol. 2022 Apr 8;13:844595. doi: 10.3389/fneur.2022.844595. eCollection 2022.

Chmara M, Wasag B, Zuk M, Kubalska J, Wegrzyn A, Bednarska-Makaruk M, Pronicka E, Wehr H, Defesche JC, Rynkiewicz A, Limon J. Molecular characterization of Polish patients with familial hypercholesterolemia: novel and recurrent LDLR mutations. J Appl Genet. 2010;51(1):95-106. doi: 10.1007/BF03195716.

Choi JH, Balasubramanian R, Lee PH, Shaw ND, Hall JE, Plummer L, Buck CL, Kottler ML, Jarzabek K, Wołczynski S, Quinton R, Latronico AC, Dode C, Ogata T, Kim HG, Layman LC, Gusella JF, Crowley WF Jr. Expanding the Spectrum of Founder Mutations Causing Isolated Gonadotropin-Releasing Hormone Deficiency. J Clin Endocrinol Metab. 2015 Oct;100(10):E1378-85. doi: 10.1210/jc.2015-2262. Epub 2015 Jul 24.

Chong JX, Ouwenga R, Anderson RL, Waggoner DJ, Ober C. A population-based study of autosomal-recessive disease-causing mutations in a founder population. Am J Hum Genet. 2012 Oct 5;91(4):608-20. doi: 10.1016/j.ajhg.2012.08.007. Epub 2012 Sep 13.

Chorostowska-Wynimko J, Struniawski R, Sliwinski P, Wajda B, Czajkowska-Malinowska M. The national alpha-1 antitrypsin deficiency registry in Poland. COPD. 2015 May;12 Suppl 1:22-6. doi: 10.3109/15412555.2015.1021915.

Chrobáková T, Hermanová M, Kroupová I, Vondrácek P, Maríková T, Mazanec R, Zámecník J, Stanek J, Havlová M, Fajkusová L. Mutations in Czech LGMD2A patients revealed by analysis of calpain3 mRNA and their phenotypic outcome. Neuromuscul Disord. 2004 Oct;14(10):659-65. doi: 10.1016/j.nmd.2004.05.005.

Cierniková S, Tomka M, Kovác M, Stevurková V, Zajac V. Ashkenazi founder BRCA1/BRCA2 mutations in Slovak hereditary breast and/or ovarian cancer families. Neoplasma. 2006;53(2):97-102.

Couce ML, Bóveda MD, Fernández-Marmiesse A, Mirás A, Pérez B, Desviat LR, Fraga JM. Molecular epidemiology and BH4-responsiveness in patients with phenylalanine hydroxylase deficiency from Galicia region of Spain. Gene. 2013 May 25;521(1):100-4. doi: 10.1016/j.gene.2013.03.004. Epub 2013 Mar 14.

Csikós M, Szocs HI, Lászik A, Mecklenbeck S, Horváth A, Kárpáti S, Bruckner-Tuderman L. High frequency of the 425A-->G splice-site mutation and novel mutations of the COL7A1 gene in central Europe: significance for future mutation detection strategies in dystrophic epidermolysis bullosa. Br J Dermatol. 2005 May;152(5):879-86. doi: 10.1111/j.1365-2133.2005.06542.x.

Cybulski C, Kluźniak W, Huzarski T, Wokołorczyk D, Kashyap A, Rusak B, Stempa K, Gronwald J, Szymiczek A, Bagherzadeh M, Jakubowska A, Dębniak T, Lener M, Rudnicka H, Szwiec M, Jarkiewicz-Tretyn J, Stawicka M, Domagała P, Narod SA, Lubiński J, Akbari MR; Polish Hereditary Breast Cancer Consortium. The spectrum of mutations predisposing to familial breast cancer in Poland. Int J Cancer. 2019 Dec 15;145(12):3311-3320. doi: 10.1002/ijc.32492. Epub 2019 Jun 26.

Cybulski C, Wokołorczyk D, Huzarski T, Byrski T, Gronwald J, Górski B, Debniak T, Masojć B, Jakubowska A, van de Wetering T, Narod SA, Lubiński J. A deletion in CHEK2 of 5,395 bp predisposes to breast cancer in Poland. Breast Cancer Res Treat. 2007 Mar;102(1):119-22. doi: 10.1007/s10549-006-9320-y. Epub 2006 Aug 8.

Cybulski C, Zamani N, Kluźniak W, Milano L, Wokołorczyk D, Stempa K, Rudnicka H, Zhang S, Zadeh M, Huzarski T, Jakubowska A, Dębniak T, Lener M, Szwiec M, Domagała P, Samani AA, Narod S, Gronwald J, Masson JY, Lubiński J, Akbari MR. Variants in ATRIP are associated with breast cancer susceptibility in the Polish population and UK Biobank. Am J Hum Genet. 2023 Apr 6;110(4):648-662. doi: 10.1016/j.ajhg.2023.03.002. Epub 2023 Mar 27.

Danhauser K, Alhaddad B, Makowski C, Piekutowska-Abramczuk D, Syrbe S, Gomez-Ospina N, Manning MA, Kostera-Pruszczyk A, Krahn-Peper C, Berutti R, Kovács-Nagy R, Gusic M, Graf E, Laugwitz L, Röblitz M, Wroblewski A, Hartmann H, Das AM, Bültmann E, Fang F, Xu M, Schatz UA, Karall D, Zellner H, Haberlandt E, Feichtinger RG, Mayr JA, Meitinger T, Prokisch H, Strom TM, Płoski R, Hoffmann GF, Pronicki M, Bonnen PE, Morlot S, Haack TB. Bi-allelic ADPRHL2 Mutations Cause Neurodegeneration with Developmental Delay, Ataxia, and Axonal Neuropathy. Am J Hum Genet. 2018 Nov 1;103(5):817-825. doi: 10.1016/j.ajhg.2018.10.005. Epub 2018 Oct 25.

Danishevich A, Bilyalov A, Nikolaev S, Khalikov N, Isaeva D, Levina Y, Makarova M, Nemtsova M, Chernevskiy D, Sagaydak O, Baranova E, Vorontsova M, Byakhova M, Semenova A, Galkin V, Khatkov I, Gadzhieva S, Bodunova N. CDKN2A Gene Mutations: Implications for Hereditary Cancer Syndromes. Biomedicines. 2023 Dec 18;11(12):3343. doi: 10.3390/biomedicines11123343.

Dávid É, Török D, Farkas K, Nagy N, Horváth E, Kiss Z, Oroszlán G, Balogh M, Széll M. Genetic investigation confirmed the clinical phenotype of congenital chloride diarrhea in a Hungarian patient: a case report. BMC Pediatr. 2019 Jan 11;19(1):16. doi: 10.1186/s12887-019-1390-1.

Debeljak M, Toplak N, Abazi N, Szabados B, Mulaosmanović V, Radović J, Perko D, Vojnović J, Constantin T, Kuzmanovska D, Avčin T. The carrier rate and spectrum of MEFV gene mutations in central and southeastern European populations. Clin Exp Rheumatol. 2015 Nov-Dec;33(6 Suppl 94):S19-23. Epub 2015 Sep 24.

De Rosa M, Galatola M, Borriello S, Duraturo F, Masone S, Izzo P. Implication of adenomatous polyposis coli and MUTYH mutations in familial colorectal polyposis. Dis Colon Rectum. 2009 Feb;52(2):268-74. doi: 10.1007/DCR.0b013e318197d15c.

Dedoussis GV, Schmidt H, Genschel J. LDL-receptor mutations in Europe.Hum Mutat. 2004 Dec;24(6):443-59. doi: 10.1002/humu.20105.

Demosthenous P, Voskarides K, Stylianou K, Hadjigavriel M, Arsali M, Patsias C, Georgaki E, Zirogiannis P, Stavrou C, Daphnis E, Pierides A, Deltas C; Hellenic Nephrogenetics Research Consortium. X-linked Alport syndrome in Hellenic families: phenotypic heterogeneity and mutations near interruptions of the collagen domain in COL4A5. Clin Genet. 2012 Mar;81(3):240-8. doi: 10.1111/j.1399-0004.2011.01647.x. Epub 2011 Mar 13.

Dempsey-Nunez L, Illson ML, Kent J, Huang Q, Brebner A, Watkins D, Gilfix BM, Wittwer CT, Rosenblatt DS. High resolution melting analysis of the MMAA gene in patients with cblA and in those with undiagnosed methylmalonic aciduria. Mol Genet Metab. 2012 Nov;107(3):363-7. doi: 10.1016/j.ymgme.2012.09.012. Epub 2012 Sep 15. PMID: 23026888

Diakou M, Miltiadous G, Xenophontos SL, Manoli P, Cariolou MA, Elisaf M. Spectrum of LDLR gene mutations, including a novel mutation causing familial hypercholesterolaemia, in North-western Greece. Eur J Intern Med. 2011 Oct;22(5):e55-9. doi: 10.1016/j.ejim.2011.01.003. Epub 2011 Feb 11.

Djordjevic M, Klaassen K, Sarajlija A, Tosic N, Zukic B, Kecman B, Ugrin M, Spasovski V, Pavlovic S, Stojiljkovic M. Molecular Genetics and Genotype-Based Estimation of BH4-Responsiveness in Serbian PKU Patients: Spotlight on Phenotypic Implications of p.L48S. JIMD Rep. 2013;9:49-58. doi: 10.1007/8904_2012_178. Epub 2012 Oct 13.

Dobrowolski SF, Heintz C, Miller T, Ellingson C, Ellingson C, Ozer I, Gökçay G, Baykal T, Thöny B, Demirkol M, Blau N. Molecular genetics and impact of residual in vitro phenylalanine hydroxylase activity on tetrahydrobiopterin responsiveness in Turkish PKU population. Mol Genet Metab. 2011 Feb;102(2):116-21. doi: 10.1016/j.ymgme.2010.11.158. Epub 2010 Nov 18.

Dodova RI, Mitkova AV, Dacheva DR, Hadjo LB, Vlahova AI, -Hadjieva MST, Valev SS, Caulevska MM, Popova SD, Popov IE, Dikov TI, Sedloev TA, Ionkov AS, Timcheva KV, Christova SL, Kremensky IM, Mitev VI, Kaneva RP. Spectrum and frequencies of BRCA1/2 mutations in Bulgarian high risk breast cancer patients. BMC Cancer. 2015 Jul 17;15:523. doi: 10.1186/s12885-015-1516-2.

Dolzan V, Stopar-Obreza M, Zerjav-Tansek M, Breskvar K, Krzisnik C, Battelino T. Mutational spectrum of congenital adrenal hyperplasia in Slovenian patients: a novel Ala15Thr mutation and Pro30Leu within a larger gene conversion associated with a severe form of the disease. Eur J Endocrinol. 2003 Aug;149(2):137-44. doi: 10.1530/eje.0.1490137.

Dörk T, Macek M Jr, Mekus F, Tümmler B, Tzountzouris J, Casals T, Krebsová A, Koudová M, Sakmaryová I, Macek M Sr, Vávrová V, Zemková D, Ginter E, Petrova NV, Ivaschenko T, Baranov V, Witt M, Pogorzelski A, Bal J, Zékanowsky C, Wagner K, Stuhrmann M, Bauer I, Seydewitz HH, Neumann T, Jakubiczka S. Characterization of a novel 21-kb deletion, CFTRdele2,3(21 kb), in the CFTR gene: a cystic fibrosis mutation of Slavic origin common in Central and East Europe. Hum Genet. 2000 Mar;106(3):259-68. doi: 10.1007/s004390000246.

Dorobek M, Ryniewicz B, Kabzińska D, Fidziańska A, Styczyńska M, Hausmanowa-Petrusewicz I. The Frequency of c.550delA Mutation of the CANP3 Gene in the Polish LGMD2A Population. Genet Test Mol Biomarkers. 2015 Nov;19(11):637-40. doi: 10.1089/gtmb.2015.0131. Epub 2015 Oct 20.

Dragasević NT, Culjković B, Klein C, Ristić A, Keckarević M, Topisirović I, Vukosavić S, Svetel M, Kock N, Stefanova E, Romac S, Kostić VS. Frequency analysis and clinical characterization of different types of spinocerebellar ataxia in Serbian patients. Mov Disord. 2006 Feb;21(2):187-91. doi: 10.1002/mds.20687.

Dufault MR, Betz B, Wappenschmidt B, Hofmann W, Bandick K, Golla A, Pietschmann A, Nestle-Krämling C, Rhiem K, Hüttner C, von Lindern C, Dall P, Kiechle M, Untch M, Jonat W, Meindl A, Scherneck S, Niederacher D, Schmutzler RK, Arnold N. Limited relevance of the CHEK2 gene in hereditary breast cancer.Int J Cancer. 2004 Jun 20;110(3):320-5. doi: 10.1002/ijc.20073.

Dumic M, Barišic N, Kusec V, Stingl K, Skegro M, Stanimirovic A, Koehler K, Huebner A. Long-term clinical follow-up and molecular genetic findings in eight patients with triple A syndrome. Eur J Pediatr. 2012 Oct;171(10):1453-9. doi: 10.1007/s00431-012-1745-1. Epub 2012 Apr 28.

Dusatkova P, Pfäffle R, Brown MR, Akulevich N, Arnhold IJ, Kalina MA, Kot K, Krzisnik C, Lemos MC, Malikova J, Navardauskaite R, Obermannova B, Pribilincova Z, Sallai A, Stipancic G, Verkauskiene R, Cinek O, Blum WF, Parks JS, Austerlitz F, Lebl J. Genesis of two most prevalent PROP1 gene variants causing combined pituitary hormone deficiency in 21 populations. Eur J Hum Genet. 2016 Mar;24(3):415-20. doi: 10.1038/ejhg.2015.126. Epub 2015 Jun 10.

Dusatkova P, Pruhova S, Borowiec M, Vesela K, Antosik K, Lebl J, Mlynarski W, Cinek O. Ancestral mutations may cause a significant proportion of GCK-MODY. Pediatr Diabetes. 2012 Sep;13(6):489-98. doi: 10.1111/j.1399-5448.2011.00845.x. Epub 2012 Feb 15.

Dusek P, Mekle R, Skowronska M, Acosta-Cabronero J, Huelnhagen T, Robinson SD, Schubert F, Deschauer M, Els A, Ittermann B, Schottmann G, Madai VI, Paul F, Klopstock T, Kmiec T, Niendorf T, Wuerfel J, Schneider SA. Brain iron and metabolic abnormalities in C19orf12 mutation carriers: A 7.0 tesla MRI study in mitochondrial membrane protein-associated neurodegeneration. Mov Disord. 2020 Jan;35(1):142-150. doi: 10.1002/mds.27827. Epub 2019 Sep 13.

Dymerska D, Gołębiewska K, Kuświk M, Rudnicka H, Scott RJ, Billings R, Pławski A, Boruń P, Siołek M, Kozak-Klonowska B, Szwiec M, Kilar E, Huzarski T, Byrski T, Lubiński J, Kurzawski G. New EPCAM founder deletion in Polish population. Clin Genet. 2017 Dec;92(6):649-653. doi: 10.1111/cge.13026. Epub 2017 Aug 3.

Ebrahimi-Fakhari D, Cheng C, Dies K, Diplock A, Pier DB, Ryan CS, Lanpher BC, Hirst J, Chung WK, Sahin M, Rosser E, Darras B, Bennett JT; CureSPG47. Clinical and genetic characterization of AP4B1-associated SPG47. Am J Med Genet A. 2018 Feb;176(2):311-318. doi: 10.1002/ajmg.a.38561. Epub 2017 Nov 28.

Eckl KM, de Juanes S, Kurtenbach J, Nätebus M, Lugassy J, Oji V, Traupe H, Preil ML, Martínez F, Smolle J, Harel A, Krieg P, Sprecher E, Hennies HC. Molecular analysis of 250 patients with autosomal recessive congenital ichthyosis: evidence for mutation hotspots in ALOXE3 and allelic heterogeneity in ALOX12B. J Invest Dermatol. 2009 Jun;129(6):1421-8. doi: 10.1038/jid.2008.409. Epub 2009 Jan 8.

El Sissy C, Rosain J, Vieira-Martins P, Bordereau P, Gruber A, Devriese M, de Pontual L, Taha MK, Fieschi C, Picard C, Frémeaux-Bacchi V. Clinical and Genetic Spectrum of a Large Cohort With Total and Sub-total Complement Deficiencies. Front Immunol. 2019 Aug 8;10:1936. doi: 10.3389/fimmu.2019.01936. eCollection 2019.

El-Ashry MF, Abd El-Aziz MM, Wilkins S, Cheetham ME, Wilkie SE, Hardcastle AJ, Halford S, Bayoumi AY, Ficker LA, Tuft S, Bhattacharya SS, Ebenezer ND. Identification of novel mutations in the carbohydrate sulfotransferase gene (CHST6) causing macular corneal dystrophy. Invest Ophthalmol Vis Sci. 2002 Feb;43(2):377-82.

Farrell P, Férec C, Macek M, Frischer T, Renner S, Riss K, Barton D, Repetto T, Tzetis M, Giteau K, Duno M, Rogers M, Levy H, Sahbatou M, Fichou Y, Le Maréchal C, Génin E. Estimating the age of p.(Phe508del) with family studies of geographically distinct European populations and the early spread of cystic fibrosis. Eur J Hum Genet. 2018 Dec;26(12):1832-1839. doi: 10.1038/s41431-018-0234-z. Epub 2018 Aug 8.

Fierabracci A, Lanzillotta M, Vorgučin I, Palma A, Katanić D, Betterle C. Report of two siblings with APECED in Serbia: is there a founder effect of c.769C>T AIRE genotype? Ital J Pediatr. 2021 Jun 2;47(1):126. doi: 10.1186/s13052-021-01075-8.

Fichna JP, Macias A, Piechota M, Korostyński M, Potulska-Chromik A, Redowicz MJ, Zekanowski C. Whole-exome sequencing identifies novel pathogenic mutations and putative phenotype-influencing variants in Polish limb-girdle muscular dystrophy patients. Hum Genomics. 2018 Jul 3;12(1):34. doi: 10.1186/s40246-018-0167-1

Figueres ML, Linglart A, Bienaime F, Allain-Launay E, Roussey-Kessler G, Ryckewaert A, Kottler ML, Hourmant M. Kidney function and influence of sunlight exposure in patients with impaired 24-hydroxylation of vitamin D due to CYP24A1 mutations. Am J Kidney Dis. 2015 Jan;65(1):122-6. doi: 10.1053/j.ajkd.2014.06.037. Epub 2014 Nov 4.

Firneisz G, Lakatos PL, Szalay F, Polli C, Glant TT, Ferenci P. Common mutations of ATP7B in Wilson disease patients from Hungary. Am J Med Genet. 2002 Feb 15;108(1):23-8. doi: 10.1002/ajmg.10220

Fischer J, Bouadjar B, Heilig R, Huber M, Lefèvre C, Jobard F, Macari F, Bakija-Konsuo A, Ait-Belkacem F, Weissenbach J, Lathrop M, Hohl D, Prud'homme JF. Mutations in the gene encoding SLURP-1 in Mal de Meleda. Hum Mol Genet. 2001 Apr 1;10(8):875-80. doi: 10.1093/hmg/10.8.875.

Gabčová D, Vohnout B, Staníková D, Hučková M, Kadurová M, Debreová M, Kozárová M, Fábryová Ľ, Staník J, Klimeš I, Rašlová K, Gašperiková D. The molecular genetic background of familial hypercholesterolemia: data from the Slovak nation-wide survey. Physiol Res. 2017 Mar 31;66(1):75-84. doi: 10.33549/physiolres.933348. Epub 2016 Nov 8.

Gigante M, Santangelo L, Diella S, Caridi G, Argentiero L, D"Alessandro MM, Martino M, Stea ED, Ardissino G, Carbone V, Pepe S, Scrutinio D, Maringhini S, Ghiggeri GM, Grandaliano G, Giordano M, Gesualdo L. Mutational Spectrum of CYP24A1 Gene in a Cohort of Italian Patients with Idiopathic Infantile Hypercalcemia.Nephron. 2016;133(3):193-204. doi: 10.1159/000446663. Epub 2016 Jul 9.

Glavač D, Jarc-Vidmar M, Vrabec K, Ravnik-Glavač M, Fakin A, Hawlina M. Clinical and genetic heterogeneity in Slovenian patients with BEST disease. Acta Ophthalmol. 2016 Dec;94(8):e786-e794. doi: 10.1111/aos.13202. Epub 2016 Oct 24.

Gomes A, Dedoussis GV. Geographic distribution of ATP7B mutations in Wilson disease. Ann Hum Biol. 2016;43(1):1-8. doi: 10.3109/03014460.2015.1051492. Epub 2015 Jul 24.

Gornjec A, Novakovic S, Stegel V, Hocevar M, Pohar Marinsek Z, Gazic B, Krajc M, Skof E. Cytology material is equivalent to tumor tissue in determining mutations of BRCA 1/2 genes in patients with tubo-ovarian high grade serous carcinoma.BMC Cancer. 2019 Apr 2;19(1):296. doi: 10.1186/s12885-019-5535-2.

Gorodetska I, Serga S, Lahuta T, Ostapchenko L, Demydov S, Khranovska N, Skachkova O, Inomistova M, Kolesnik O, Svintsitsky V, Tsip N, Peresunko A, Kmit' N, Manzhura O, Rossokha Z, Popova O, Salomakhina H, Kyriachenko S, Kozeretska I. Prevalence of two BRCA1 mutations, 5382insC and 300T > G, in ovarian cancer patients from Ukraine. Fam Cancer. 2017 Oct;16(4):471-476. doi: 10.1007/s10689-017-9978-9.

Górski B, Cybulski C, Huzarski T, Byrski T, Gronwald J, Jakubowska A, Stawicka M, Gozdecka-Grodecka S, Szwiec M, Urbański K, Mituś J, Marczyk E, Dziuba J, Wandzel P, Surdyka D, Haus O, Janiszewska H, Debniak T, Tołoczko-Grabarek A, Medrek K, Masojć B, Mierzejewski M, Kowalska E, Narod SA, Lubiński J. Breast cancer predisposing alleles in Poland. Breast Cancer Res Treat. 2005 Jul;92(1):19-24. doi: 10.1007/s10549-005-1409-1.

Groselj U, Tansek MZ, Kovac J, Hovnik T, Podkrajsek KT, Battelino T. Five novel mutations and two large deletions in a population analysis of the phenylalanine hydroxylase gene. Mol Genet Metab. 2012 Jun;106(2):142-8. doi: 10.1016/j.ymgme.2012.03.015. Epub 2012 Apr 1.

Gruchota J, Pronicka E, Korniszewski L, Stolarski B, Pollak A, Rogaszewska M, Płoski R. Aldolase B mutations and prevalence of hereditary fructose intolerance in a Polish population. Mol Genet Metab. 2006 Apr;87(4):376-8. doi: 10.1016/j.ymgme.2005.11.010. Epub 2006 Jan 10.

Gruenauer-Kloevekorn C, Braeutigam S, Heinritz W, Froster UG, Duncker GI. Macular corneal dystrophy: mutational spectrum in German patients, novel mutations and therapeutic options. Graefes Arch Clin Exp Ophthalmol. 2008 Oct;246(10):1441-7. doi: 10.1007/s00417-008-0836-1. Epub 2008 May 24.

Grumach AS, Kirschfink M. Are complement deficiencies really rare? Overview on prevalence, clinical importance and modern diagnostic approach. Mol Immunol. 2014 Oct;61(2):110-7. doi: 10.1016/j.molimm.2014.06.030. Epub 2014 Jul 15.

Gundorova P, Stepanova AA, Kuznetsova IA, Kutsev SI, Polyakov AV. Genotypes of 2579 patients with phenylketonuria reveal a high rate of BH4 non-responders in Russia. PLoS One. 2019 Jan 22;14(1):e0211048. doi: 10.1371/journal.pone.0211048. eCollection 2019.

Hackmann K, Kuhlee F, Betcheva-Krajcir E, Kahlert AK, Mackenroth L, Klink B, Di Donato N, Tzschach A, Kast K, Wimberger P, Schrock E, Rump A. Ready to clone: CNV detection and breakpoint fine-mapping in breast and ovarian cancer susceptibility genes by high-resolution array CGH. Breast Cancer Res Treat. 2016 Oct;159(3):585-90. doi: 10.1007/s10549-016-3956-z. Epub 2016 Aug 31.

Hamel N, Feng BJ, Foretova L, Stoppa-Lyonnet D, Narod SA, Imyanitov E, Sinilnikova O, Tihomirova L, Lubinski J, Gronwald J, Gorski B, Hansen Tv, Nielsen FC, Thomassen M, Yannoukakos D, Konstantopoulou I, Zajac V, Ciernikova S, Couch FJ, Greenwood CM, Goldgar DE, Foulkes WD. On the

origin and diffusion of BRCA1 c.5266dupC (5382insC) in European populations. Eur J Hum Genet. 2011 Mar;19(3):300-6. doi: 10.1038/ejhg.2010.203. Epub 2010 Dec 1.

Hartig MB, Iuso A, Haack T, Kmiec T, Jurkiewicz E, Heim K, Roeber S, Tarabin V, Dusi S, Krajewska-Walasek M, Jozwiak S, Hempel M, Winkelmann J, Elstner M, Oexle K, Klopstock T, Mueller-Felber W, Gasser T, Trenkwalder C, Tiranti V, Kretzschmar H, Schmitz G, Strom TM, Meitinger T, Prokisch H. Absence of an orphan mitochondrial protein, c19orf12, causes a distinct clinical subtype of neurodegeneration with brain iron accumulation. Am J Hum Genet. 2011 Oct 7;89(4):543-50. doi: 10.1016/j.ajhg.2011.09.007.

Heramb C, Wangensteen T, Grindedal EM, Ariansen SL, Lothe S, Heimdal KR, Mæhle L. BRCA1 and BRCA2 mutation spectrum - an update on mutation distribution in a large cancer genetics clinic in Norway. Hered Cancer Clin Pract. 2018 Jan 10;16:3. doi: 10.1186/s13053-017-0085-6. eCollection 2018.

Hiljadnikova-Bajro M, Josifovski T, Panovski M, Dimovski AJ. A novel germline MLH1 mutation causing Lynch Syndrome in patients from the Republic of Macedonia. Croat Med J. 2012 Oct;53(5):496-501. doi: 10.3325/cmj.2012.53.496.

Höben IM, Hjeij R, Olbrich H, Dougherty GW, Nöthe-Menchen T, Aprea I, Frank D, Pennekamp P, Dworniczak B, Wallmeier J, Raidt J, Nielsen KG, Philipsen MC, Santamaria F, Venditto L, Amirav I, Mussaffi H, Prenzel F, Wu K, Bakey Z, Schmidts M, Loges NT, Omran H. Mutations in C11orf70 Cause Primary Ciliary Dyskinesia with Randomization of Left/Right Body Asymmetry Due to Defects of Outer and Inner Dynein Arms. Am J Hum Genet. 2018 May 3;102(5):973-984. doi: 10.1016/j.ajhg.2018.03.025.

Höglund P, Auranen M, Socha J, Popinska K, Nazer H, Rajaram U, Al Sanie A, Al-Ghanim M, Holmberg C, de la Chapelle A, Kere J. Genetic background of congenital chloride diarrhea in high-incidence populations: Finland, Poland, and Saudi Arabia and Kuwait. Am J Hum Genet. 1998 Sep;63(3):760-8. doi: 10.1086/301998.

Hogarth P, Gregory A, Kruer MC, Sanford L, Wagoner W, Natowicz MR, Egel RT, Subramony SH, Goldman JG, Berry-Kravis E, Foulds NC, Hammans SR, Desguerre I, Rodriguez D, Wilson C, Diedrich A, Green S, Tran H, Reese L, Woltjer RL, Hayflick SJ. New NBIA subtype: genetic, clinical, pathologic, and radiographic features of MPAN Neurology. 2013 Jan 15;80(3):268-75. doi: 10.1212/WNL.0b013e31827e07be. Epub 2012 Dec 26.

Illarioshkin SN, Slominsky PA, Ovchinnikov IV, Markova ED, Miklina NI, Klyushnikov SA, Shadrina M, Vereshchagin NV, Limborskaya SA, Ivanova-Smolenskaya IA. Spinocerebellar ataxia type 1 in Russia. J Neurol. 1996 Jul;243(7):506-10. doi: 10.1007/BF00886871.

Isbrandt D, Arlt G, Brooks DA, Hopwood JJ, von Figura K, Peters C. Mucopolysaccharidosis VI (Maroteaux-Lamy syndrome): six unique arylsulfatase B gene alleles causing variable disease phenotypes. Am J Hum Genet. 1994 Mar;54(3):454-63.

Ivanova ME, Trubilin VN, Atarshchikov DS, Demchinsky AM, Strelnikov VV, Tanas AS, Orlova OM, Machalov AS, Overchenko KV, Markova TV, Golenkova DM, Anoshkin KI, Volodin IV, Zaletaev DV, Pulin AA, Nadelyaeva II, Kalinkin AI, Barh D. Genetic screening of Russian Usher syndrome patients toward selection for gene therapy. Ophthalmic Genet. 2018 Dec;39(6):706-713. doi: 10.1080/13816810.2018.1532527. Epub 2018 Oct 25. PMID: 30358468

Jakimovska M, Maleva Kostovska I, Popovska-Jankovic K, Kubelka-Sabit K, Karadjozov M, Stojanovska L, Arsovski A, Smichkoska S, Lazarova E, Jakimovska Dimitrovska M, Plaseska-Karanfilska D. BRCA1 and BRCA2 germline variants in breast cancer patients from the Republic of Macedonia. Breast Cancer Res Treat. 2018 Apr;168(3):745-753. doi: 10.1007/s10549-017-4642-5. Epub 2018 Jan 15.

Janatova M, Kleibl Z, Stribrna J, Panczak A, Vesela K, Zimovjanova M, Kleiblova P, Dundr P, Soukupova J, Pohlreich P. The PALB2 gene is a strong candidate for clinical testing in BRCA1- and BRCA2-negative hereditary breast cancer. Cancer Epidemiol Biomarkers Prev. 2013 Dec;22(12):2323-32. doi: 10.1158/1055-9965.EPI-13-0745-T. Epub 2013 Oct 17.

Janavičius R, Rudaitis V, Feng BJ, Ozolina S, Griškevičius L, Goldgar D, Tihomirova L. Haplotype analysis and ancient origin of the BRCA1 c.4035delA Baltic founder mutation. Eur J Med Genet. 2013 Mar;56(3):125-30. doi: 10.1016/j.ejmg.2012.12.007. Epub 2012 Dec 27.

Janavičius R, Rudaitis V, Mickys U, Elsakov P, Griškevičius L. Comprehensive BRCA1 and BRCA2 mutational profile in Lithuania. Cancer Genet. 2014 May;207(5):195-205. doi: 10.1016/j.cancergen.2014.05.002. Epub 2014 May 10.

Janavičius R. Founder BRCA1/2 mutations in the Europe: implications for hereditary breast-ovarian cancer prevention and control. EPMA J. 2010 Sep;1(3):397-412. doi: 10.1007/s13167-010-0037-y. Epub 2010 Jun 27.

Jankowska I, Czubkowski P, Rokicki D, Lipiński P, Piekutowska-Abramczuk D, Ciara E, Płoski R, Kaliciński P, Szymczak M, Pawłowska J, Socha P. Acute liver failure due to DGUOK deficiency-is liver transplantation justified? Clin Res Hepatol Gastroenterol. 2020 Apr 8:S2210-7401(20)30082-6. doi: 10.1016/j.clinre.2020.02.018.

Jankowski M, Daca-Roszak P, Trautmann A, Milovanova A, Balasz-Chmielewska I, Lipska-Ziętkiewicz B. Clinical delineation of the NUP93 glomerulopathy prevalent in Central and Eastern Europe. Pediatric Nephrol. 2021, 36, 3306 (In Abstracts of the 53rd ESPN Annual Meeting, Amsterdam, The Netherlands, September 2021)

Jaruzelska J, Matuszak R, Lyonnet S, Rey F, Rey J, Filipowicz J, Borski K, Munnich A. Genetic background of clinical homogeneity of phenylketonuria in Poland. J Med Genet. 1993 Mar;30(3):232-4. doi: 10.1136/jmg.30.3.232.

Jerábková B, Kopecková L, Bucková H, Veselý K, Valícková J, Fajkusová L. Analysis of the COL7A1 gene in Czech patients with dystrophic epidermolysis bullosa reveals novel and recurrent mutations. J Dermatol Sci. 2010 Aug;59(2):136-40. doi: 10.1016/j.jdermsci.2010.05.007. Epub 2010 Jun 8.

Johnson CA, Densen P, Hurford RK Jr, Colten HR, Wetsel RA. Type I human complement C2 deficiency. A 28-base pair gene deletion causes skipping of exon 6 during RNA splicing. J Biol Chem. 1992 May 5;267(13):9347-53.

Jurecka A, Piotrowska E, Cimbalistiene L, Gusina N, Sobczyńska A, Czartoryska B, Czerska K, Õunap K, Węgrzyn G, Tylki-Szymańska A. Molecular analysis of mucopolysaccharidosis type VI in Poland, Belarus, Lithuania and Estonia. Mol Genet Metab. 2012 Feb;105(2):237-43. doi: 10.1016/j.ymgme.2011.11.003. Epub 2011 Nov 11.

Jurecka A, Zakharova E, Cimbalistiene L, Gusina N, Malinova V, Różdżyńska-Świątkowska A, Golda A, Kulpanovich A, Kaldenovna Abdilova G, Voskoboeva E, Tylki-Szymańska A. Mucopolysaccharidosis type VI in Russia, Kazakhstan, and Central and Eastern Europe. Pediatr Int. 2014 Aug;56(4):520-5. doi: 10.1111/ped.12281. Epub 2014 Apr 1.

Jurecka A, Zikanova M, Tylki-Szymanska A, Krijt J, Bogdanska A, Gradowska W, Mullerova K, Sykut-Cegielska J, Kmoch S, Pronicka E. Clinical, biochemical and molecular findings in seven Polish patients with adenylosuccinate lyase deficiency. Mol Genet Metab. 2008 Aug;94(4):435-42. doi: 10.1016/j.ymgme.2008.04.013. Epub 2008 Jun 3.

Kabzińska D, Strugalska-Cynowska H, Kostera-Pruszczyk A, Ryniewicz B, Posmyk R, Midro A, Seeman P, Báranková L, Zimoń M, Baets J, Timmerman V, Guergueltcheva V, Tournev I, Sarafov S, De Jonghe P, Jordanova A, Hausmanowa-Petrusewicz I, Kochański A. L239F founder mutation in GDAP1 is associated with a mild Charcot-Marie-Tooth type 4C4 (CMT4C4) phenotype. Neurogenetics. 2010 Jul;11(3):357-66. doi: 10.1007/s10048-010-0237-6. Epub 2010 Mar 16.

Kapplinger JD, Landstrom AP, Bos JM, Salisbury BA, Callis TE, Ackerman MJ. Distinguishing hypertrophic cardiomyopathy-associated mutations from background genetic noise. J Cardiovasc Transl Res. 2014 Apr;7(3):347-61. doi: 10.1007/s12265-014-9542-z. Epub 2014 Feb 8.

Karageorgos L, Brooks DA, Pollard A, Melville EL, Hein LK, Clements PR, Ketteridge D, Swiedler SJ, Beck M, Giugliani R, Harmatz P, Wraith JE, Guffon N, Leão Teles E, Sá Miranda MC, Hopwood JJ. Mutational analysis of 105 mucopolysaccharidosis type VI patients Hum Mutat. 2007 Sep;28(9):897-903. doi: 10.1002/humu.20534.

Kaufman B, Laitman Y, Gronwald J, Lubinski J, Friedman E. Haplotype of the C61G BRCA1 mutation in Polish and Jewish individuals. Genet Test Mol Biomarkers. 2009 Aug;13(4):465-9. doi: 10.1089/gtmb.2009.0001.

Kawakita R, Hosokawa Y, Fujimaru R, Tamagawa N, Urakami T, Takasawa K, Moriya K, Mizuno H, Maruo Y, Takuwa M, Nagasaka H, Nishi Y, Yamamoto Y, Aizu K, Yorifuji T. Molecular and clinical characterization of glucokinase maturity-onset diabetes of the young (GCK-MODY) in Japanese patients. Diabet Med. 2014 Nov;31(11):1357-62. doi: 10.1111/dme.12487. Epub 2014 May 24.

Kechin A, Boyarskikh U, Barinov A, Tanas A, Kazakova S, Zhevlova A, Khrapov E, Subbotin S, Mishukova O, Kekeeva T, Demidova I, Filipenko M. A spectrum of BRCA1 and BRCA2 germline deleterious variants in ovarian cancer in Russia. Breast Cancer Res Treat. 2023 Jan;197(2):387-395. doi: 10.1007/s10549-022-06782-2. Epub 2022 Nov 11.

Kecmanović M, Jović N, Keckarević-Marković M, Keckarević D, Stevanović G, Ignjatović P, Romac S. Clinical and genetic data on Lafora disease patients of Serbian/Montenegrin origin. Clin Genet. 2016 Jan;89(1):104-8. doi: 10.1111/cge.12570. Epub 2015 Mar 19.

Keckarevic Markovic MP, Dackovic J, Mladenovic J, Milic-Rasic V, Kecmanovic M, Keckarevic D, Romac S. An algorithm for genetic testing of Serbian patients with demyelinating Charcot-Marie-Tooth. Genet Test Mol Biomarkers. 2013 Jan;17(1):85-7. doi: 10.1089/gtmb.2012.0238. Epub 2012 Nov 19.

Kerti A, Csohány R, Szabó A, Arkossy O, Sallay P, Moriniére V, Vega-Warner V, Nyírő G, Lakatos O, Szabó T, Lipska BS, Schaefer F, Antignac C, Reusz G, Tulassay T, Tory K. NPHS2 p.V290M mutation in late-onset steroid-resistant nephrotic syndrome. Pediatr Nephrol. 2013 May;28(5):751-7. doi: 10.1007/s00467-012-2379-2. Epub 2012 Dec 14.

Kirov A, Sarafov S, Pavlova Z, Todorov T, Chamova T, Gospodinova M, Tournev I, Mitev V, Todorova A. Founder effect of the Glu89Gln TTR mutation in the Bulgarian population. Amyloid. 2019 Dec;26(4):181-185. doi: 10.1080/13506129.2019.1634539. Epub 2019 Jul 29.

Kisiel BM, Kostrzewa G, Wlasienko P, Kruczek A, Gajdulewicz M, Maciejak D, Wisniewska M, Ploski R, Korniszewski L. Cleidocranial dysplasia in a Polish population: high frequency of the R193X mutation. Clin Genet. 2006 Aug;70(2):167-9. doi: 10.1111/j.1399-0004.2006.00648.x.

Kleibl Z, Novotny J, Bezdickova D, Malik R, Kleiblova P, Foretova L, Petruzelka L, Ilencikova D, Cinek P, Pohlreich P. The CHEK2 c.1100delC germline mutation rarely contributes to breast cancer development in the Czech Republic. Breast Cancer Res Treat. 2005 Mar;90(2):165-7. doi: 10.1007/s10549-004-4023-8.

Kleiblova P, Stolarova L, Krizova K, Lhota F, Hojny J, Zemankova P, Havranek O, Vocka M, Cerna M, Lhotova K, Borecka M, Janatova M, Soukupova J, Sevcik J, Zimovjanova M, Kotlas J, Panczak A, Vesela K, Cervenkova J, Schneiderova M, Burocziova M, Burdova K, Stranecky V, Foretova L, Machackova E, Tavandzis S, Kmoch S, Macurek L, Kleibl Z. Identification of deleterious germline CHEK2 mutations and their association with breast and ovarian cancer. Int J Cancer. 2019 Oct 1;145(7):1782-1797. doi: 10.1002/ijc.32385. Epub 2019 May 20.

Kluska A, Balabas A, Piatkowska M, Czarny K, Paczkowska K, Nowakowska D, Mikula M, Ostrowski J. PALB2 mutations in BRCA1/2-mutation negative breast and ovarian cancer patients from Poland. BMC Med Genomics. 2017 Mar 9;10(1):14. doi: 10.1186/s12920-017-0251-8.

Kluska A, Kulecka M, Litwin T, Dziezyc K, Balabas A, Piatkowska M, Paziewska A, Dabrowska M, Mikula M, Kaminska D, Wiernicka A, Socha P, Czlonkowska A, Ostrowski J. Whole-exome sequencing identifies novel pathogenic variants across the ATP7B gene and some modifiers of Wilson's disease phenotype. Liver Int. 2019 Jan;39(1):177-186. doi: 10.1111/liv.13967. Epub 2018 Oct 8.

Kmoch S, Hartmannová H, Stibůrková B, Krijt J, Zikánová M, Sebesta I. Human adenylosuccinate lyase (ADSL), cloning and characterization of full-length cDNA and its isoform, gene structure and molecular basis for ADSL deficiency in six patients. Hum Mol Genet. 2000 Jun 12;9(10):1501-13. doi: 10.1093/hmg/9.10.1501.

Ko YL, Tai DY, Chen SA, Lee-Chen GJ, Chu CH, Lin MW. Linkage and mutation analysis in two Taiwanese families with long QT syndrome. J Formos Med Assoc. 2001 Nov;100(11):767-71.

Konecny M, Milly M, Zavodna K, Weismanova E, Gregorova J, Mlkva I, Ilencikova D, Kausitz J, Bartosova Z. Comprehensive genetic characterization of hereditary breast/ovarian cancer families from Slovakia. Breast Cancer Res Treat. 2011 Feb;126(1):119-30. doi: 10.1007/s10549-010-1325-x. Epub 2011 Jan 4.

Kopečková L, Bučková H, Kýrová J, Gaillyová R, Němečková J, Jeřábková B, Veselý K, Stehlíková K, Fajkusová L. Ten years of DNA diagnostics of epidermolysis bullosa in the Czech Republic.Br J Dermatol. 2016 Jun;174(6):1388-91. doi: 10.1111/bjd.14370. Epub 2016 Mar 1.

Kopitar AN, Markelj G, Oražem M, Blazina Š, Avčin T, Ihan A, Debeljak M. Flow Cytometric Determination of Actin Polymerization in Peripheral Blood Leukocytes Effectively Discriminate Patients With Homozygous Mutation in ARPC1B From Asymptomatic Carriers and Normal Controls. Front Immunol. 2019 Jul 16;10:1632. doi: 10.3389/fimmu.2019.01632. eCollection 2019.

Kostovska IM, Jakovchevska S, Özdemir MJ, Kiprijanovska S, Kubelka-Sabit K, Jasar D, Iljovska M, Lazareva E, Smichkoska S, Plaseska-Karanfilska D. The highest frequency of BRCA1 c.3700_3704del detected among Albanians from Kosovo. Rep Pract Oncol Radiother. 2022 May 19;27(2):303-309. doi: 10.5603/RPOR.a2022.0030. eCollection 2022

Kovács G, Kalmár T, Endreffy E, Ondrik Z, Iványi B, Rikker C, Haszon I, Túri S, Sinkó M, Bereczki C, Maróti Z. Efficient Targeted Next Generation Sequencing-Based Workflow for Differential Diagnosis of Alport-Related Disorders. PLoS One. 2016 Mar 2;11(3):e0149241. doi: 10.1371/journal.pone.0149241. eCollection 2016.

Kozák L, Francová H, Hrabincová E, Procházková D, Jüttnerová V, Bzdúch V, Simek P. Smith-Lemli-Opitz syndrome: molecular-genetic analysis of ten families.J Inherit Metab Dis. 2000 Jun;23(4):409-12. doi: 10.1023/a:1005616321794.

Kozak L, Hrabincova E, Kintr J, Horky O, Zapletalova P, Blahakova I, Mejstrik P, Prochazkova D. Identification and characterization of large deletions in the phenylalanine hydroxylase (PAH) gene by MLPA: evidence for both homologous and non-homologous mechanisms of rearrangement. Mol Genet Metab. 2006 Dec;89(4):300-9. doi: 10.1016/j.ymgme.2006.06.007. Epub 2006 Aug 22.

Kozák L, Kuhrová V, Blazková M, Romano V, Fajkusová L, Dvoráková D, Pijácková A. Phenylketonuria mutations and their relation to RFLP haplotypes at the PAH locus in Czech PKU families. Hum Genet. 1995 Oct;96(4):472-6. doi: 10.1007/BF00191809.

Krajc M, Zadnik V, Novaković S, Stegel V, Teugels E, Bešič N, Hočevar M, Vakselj A, De Grève J, Zgajnar J. Geographical distribution of Slovenian BRCA1/2 families according to family origin: implications for genetic screening. Clin Genet. 2014 Jan;85(1):59-63. doi: 10.1111/cge.12119. Epub 2013 Mar 11.

Křenková P, Piskáčková T, Holubová A, Balaščaková M, Krulišová V, Čamajová J, Turnovec M, Libik M, Norambuena P, Štambergová A, Dvořáková L, Skalická V, Bartošová J, Kučerová T, Fila L, Zemková D, Vávrová V, Koudová M, Macek M, Krebsová A, Macek M Jr. Distribution of CFTR mutations in the Czech population: positive impact of integrated clinical and laboratory expertise, detection of novel/de novo alleles and relevance for related/derived populations. J Cyst Fibros. 2013 Sep;12(5):532-7. doi: 10.1016/j.jcf.2012.12.002. Epub 2012 Dec 29.

Krivokuca A, Dobricic J, Brankovic-Magic M. CHEK2 1100delC and Del5395bp mutations in BRCA-negative individuals from Serbian hereditary breast and ovarian cancer families. J BUON. 2013 Jul-Sep;18(3):594-600.

Krysa W, Sulek A, Rakowicz M, Szirkowiec W, Zaremba J. High relative frequency of SCA1 in Poland reflecting a potential founder effect. Neurol Sci. 2016 Aug;37(8):1319-25. doi: 10.1007/s10072-016-2594-x. Epub 2016 May 19.

Kurkowiak M, Ziętkiewicz E, Greber A, Voelkel K, Wojda A, Pogorzelski A, Witt M. ZMYND10--Mutation Analysis in Slavic Patients with Primary Ciliary Dyskinesia.PLoS One. 2016 Jan 29;11(1):e0148067. doi: 10.1371/journal.pone.0148067. eCollection 2016.

Kurzawski G, Suchy J, Lener M, Kłujszo-Grabowska E, Kładny J, Safranow K, Jakubowska K, Jakubowska A, Huzarski T, Byrski T, Debniak T, Cybulski C, Gronwald J, Oszurek O, Oszutowska D, Kowalska E, Góźdź S, Niepsuj S, Słomski R, Pławski A, Łacka-Wojciechowska A, Rozmiarek A, Fiszer-Maliszewska Ł, Bebenek M, Sorokin D, Sasiadek MM, Stembalska A, Grzebieniak Z, Kilar E, Stawicka M, Godlewski D, Richter P, Brozek I, Wysocka B, Limon J, Jawień A, Banaszkiewicz Z, Janiszewska H, Kowalczyk J, Czudowska D, Scott RJ, Lubiński J. Germline MSH2 and MLH1 mutational spectrum including large rearrangements in HNPCC families from Poland (update study). Clin Genet. 2006 Jan;69(1):40-7. doi: 10.1111/j.1399-0004.2006.00550.x.

Kuźma-Kozakiewicz M, Andersen PM, Elahi E, Alavi A, Sapp PC, Morita M, Żekanowski C, Berdyński M. Putative founder effect in the Polish, Iranian and United States populations for the L144S SOD1 mutation associated with slowly uniform phenotype of amyotrophic lateral sclerosis. Amyotroph Lateral Scler Frontotemporal Degener. 2021 Feb;22(1-2):80-85. doi: 10.1080/21678421.2020.1803359. Epub 2020 Aug 10.

Kuźma-Kozakiewicz M, Berdyński M, Morita M, Takahashi Y, Kawata A, Kaida K, Kaźmierczak B, Lusakowska A, Goto J, Tsuji S, Zekanowski C, Kwieciński H. Recurrent K3E mutation in Cu/Zn superoxide dismutase gene associated with amyotrophic lateral sclerosis. Amyotroph Lateral Scler Frontotemporal Degener. 2013 Dec;14(7-8):608-14. doi: 10.3109/21678421.2013.812119. Epub 2013 Jul 30.

Laššuthová P, Brožková DŠ, Krůtová M, Neupauerová J, Haberlová J, Mazanec R, Dvořáčková N, Goldenberg Z, Seeman P. Mutations in HINT1 are one of the most frequent causes of hereditary neuropathy among Czech patients and neuromyotonia is rather an underdiagnosed symptom. Neurogenetics. 2015 Jan;16(1):43-54. doi: 10.1007/s10048-014-0427-8. Epub 2014 Oct 24.

Le Quesne Stabej P, Saihan Z, Rangesh N, Steele-Stallard HB, Ambrose J, Coffey A, Emmerson J, Haralambous E, Hughes Y, Steel KP, Luxon LM, Webster AR, Bitner-Glindzicz M. Comprehensive sequence analysis of nine Usher syndrome genes in the UK National Collaborative Usher Study. J Med Genet. 2012 Jan;49(1):27-36. doi: 10.1136/jmedgenet-2011-100468. Epub 2011 Dec 1.

Lee HS, Sambuughin N, Cervenakova L, Chapman J, Pocchiari M, Litvak S, Qi HY, Budka H, del Ser T, Furukawa H, Brown P, Gajdusek DC, Long JC, Korczyn AD, Goldfarb LG. Ancestral origins and worldwide distribution of the PRNP 200K mutation causing familial Creutzfeldt-Jakob disease. Am J Hum Genet. 1999 Apr;64(4):1063-70. doi: 10.1086/302340.

Levanat S, Musani V, Cvok ML, Susac I, Sabol M, Ozretic P, Car D, Eljuga D, Eljuga L, Eljuga D. Three novel BRCA1/BRCA2 mutations in breast/ovarian cancer families in Croatia. Gene. 2012 May 1;498(2):169-76. doi: 10.1016/j.gene.2012.02.010. Epub 2012 Feb 17.

Lipari M, Wypasek E, Karpiński M, Tomkiewicz-Pajak L, Laino L, Binni F, Giannarelli D, Rubiś P, Petkow-Dimitrow P, Undas A, Grammatico P, Bottillo I. Identification of a variant hotspot in MYBPC3 and of a novel CSRP3 autosomal recessive alteration in a cohort of Polish patients with hypertrophic cardiomyopathy. Pol Arch Intern Med. 2020 Feb 27;130(2):89-99. doi: 10.20452/pamw.15130. Epub 2020 Jan 9

Lipiński P, Różdżyńska-Świątkowska A, Iwanicka-Pronicka K, Perkowska B, Pokora P, Tylki-Szymańska A. Long-term outcome of patients with alpha-mannosidosis - A single center study. Mol Genet Metab Rep. 2021 Dec 9;30:100826. doi: 10.1016/j.ymgmr.2021.100826. eCollection 2022 Mar

Lipska BS, Balasz-Chmielewska I, Morzuch L, Wasielewski K, Vetter D, Borzecka H, Drozdz D, Firszt-Adamczyk A, Gacka E, Jarmolinski T, Ksiazek J, Kuzma-Mroczkowska E, Litwin M, Medynska A, Silska M, Szczepanska M, Tkaczyk M, Wasilewska A, Schaefer F, Zurowska A, Limon J. Mutational analysis in podocin-associated hereditary nephrotic syndrome in Polish patients: founder effect in the Kashubian population. J Appl Genet. 2013 Aug;54(3):327-33. doi: 10.1007/s13353-013-0147-z. Epub 2013 May 5.

Liskova P, Kousal B, Bujakowska K, Dudakova L. Molecular genetic basis of Usher syndrome in the Czech population. Acta Ophthalmol, 2016 94:. doi:10.1111/j.1755-3768.2016.0076

Liskova P, Veraitch B, Jirsova K, Filipec M, Neuwirth A, Ebenezer ND, Hysi PG, Hardcastle AJ, Tuft SJ, Bhattacharya SS. Sequencing of the CHST6 gene in Czech macular corneal dystrophy patients supports the evidence of a founder mutation. Br J Ophthalmol. 2008 Feb;92(2):265-7. doi: 10.1136/bjo.2007.125252. Epub 2007 Oct 25.

Liu SR, Zhao B, Wang ZJ, Wan YL, Huang YT. Clinical features and mismatch repair gene mutation screening in Chinese patients with hereditary nonpolyposis colorectal carcinoma. World J Gastroenterol. 2004 Sep 15;10(18):2647-51. doi: 10.3748/wjg.v10.i18.2647.

Ljubić H, Kalauz M, Telarović S, Ferenci P, Ostojić R, Noli MC, Lepori MB, Hrstić I, Vuković J, Premužić M, Radić D, Ravić KG, Sertić J, Merkler A, Barišić AA, Loudianos G, Vucelić B. ATP7B Gene Mutations in Croatian Patients with Wilson Disease. Genet Test Mol Biomarkers. 2016 Mar;20(3):112-7. doi: 10.1089/gtmb.2015.0213. Epub 2016 Jan 22.

Maas RR, Iwanicka-Pronicka K, Kalkan Ucar S, Alhaddad B, AlSayed M, Al-Owain MA, Al-Zaidan HI, Balasubramaniam S, Barić I, Bubshait DK, Burlina A, Christodoulou J, Chung WK, Colombo R, Darin N, Freisinger P, Garcia Silva MT, Grunewald S, Haack TB, van Hasselt PM, Hikmat O, Hörster F, Isohanni P, Ramzan K, Kovacs-Nagy R, Krumina Z, Martin-Hernandez E, Mayr JA, McClean P, De Meirleir L, Naess K, Ngu LH, Pajdowska M, Rahman S, Riordan G, Riley L, Roeben B, Rutsch F, Santer R, Schiff M, Seders M, Sequeira S, Sperl W, Staufner C, Synofzik M, Taylor RW, Trubicka J, Tsiakas K, Unal O, Wassmer E, Wedatilake Y, Wolff T, Prokisch H, Morava E, Pronicka E, Wevers RA, de Brouwer AP, Wortmann SB. Progressive deafness-dystonia due to SERAC1 mutations: A study of 67 cases. Ann Neurol. 2017 Dec;82(6):1004-1015. doi: 10.1002/ana.25110.

Machackova E, Claes K, Mikova M, Házová J, Sťahlová EH, Vasickova P, Trbusek M, Navrátilová M, Svoboda M, Foretová L. Twenty Years of BRCA1 and BRCA2 Molecular Analysis at MMCI - Current Developments for the Classification of Variants. Klin Onkol. 2019 Summer;32(Supplementum2):51-71. doi: 10.14735/amko2019S51.

Makukh H, Krenková P, Tyrkus M, Bober L, Hancárová M, Hnateyko O, Macek M Jr. A high frequency of the Cystic Fibrosis 2184insA mutation in Western Ukraine: genotype-phenotype correlations, relevance for newborn screening and genetic testing. J Cyst Fibros. 2010 Sep;9(5):371-5. doi: 10.1016/j.jcf.2010.06.001. Epub 2010 Jul 24.

Malcorps M, Amor-Barris S, Burnyte B, Vilimiene R, Armirola-Ricaurte C, Grigalioniene K, Ekshteyn A, Morkuniene A, Vaitkevicius A, De Vriendt E, Baets J, Scherer SS, Ambrozaityte L, Utkus A, Jordanova A, Peeters K. HINT1 neuropathy in Lithuania: clinical, genetic, and functional profiling. Orphanet J Rare Dis. 2022 Oct 14;17(1):374. doi: 10.1186/s13023-022-02541-0.

Mallett AJ, McCarthy HJ, Ho G, Holman K, Farnsworth E, Patel C, Fletcher JT, Mallawaarachchi A, Quinlan C, Bennetts B, Alexander SI. Massively parallel sequencing and targeted exomes in familial kidney disease can diagnose underlying genetic disorders. Kidney Int. 2017 Dec;92(6):1493-1506. doi: 10.1016/j.kint.2017.06.013. Epub 2017 Aug 23.

Marjanović IV, Selak-Djokić B, Perić S, Janković M, Arsenijević V, Basta I, Lavrnić D, Stefanova E, Stević Z. Comparison of the clinical and cognitive features of genetically positive ALS patients from the largest tertiary center in Serbia. J Neurol. 2017 Jun;264(6):1091-1098. doi: 10.1007/s00415-017-8495-y. Epub 2017 Apr 25.

Martin P, Heiskari N, Zhou J, Leinonen A, Tumelius T, Hertz JM, Barker D, Gregory M, Atkin C, Styrkarsdottir U, Neumann H, Springate J, Shows T, Pettersson E, Tryggvason K. High mutation detection rate in the COL4A5 collagen gene in suspected Alport syndrome using PCR and direct DNA sequencing. J Am Soc Nephrol. 1998 Dec;9(12):2291-301.

Maslova MYu, Krylova NS, Poteshkina N.G. Amiodaron-induced thyreotoxicosis in a patient with cardiomyopathy. Meditsinskiy Alphavit, 2019, 1, 3 (378): 50 (in Russian).

Mateju M, Stribrna J, Zikan M, Kleibl Z, Janatova M, Kormunda S, Novotny J, Soucek P, Petruzelka L, Pohlreich P. Population-based study of BRCA1/2 mutations: family history based criteria identify minority of mutation carriers. Neoplasma. 2010;57(3):280-5. doi: 10.4149/neo_2010_03_280.

Maurer MH, Hoffmann K, Sperling K, Varon R. High prevalence of the NBN gene mutation c.657-661del5 in Southeast Germany. J Appl Genet. 2010;51(2):211-4. doi: 10.1007/BF03195730.

McCarthy HJ, Bierzynska A, Wherlock M, Ognjanovic M, Kerecuk L, Hegde S, Feather S, Gilbert RD, Krischock L, Jones C, Sinha MD, Webb NJ, Christian M, Williams MM, Marks S, Koziell A, Welsh GI, Saleem MA; RADAR the UK SRNS Study Group. Simultaneous sequencing of 24 genes associated with steroid-resistant nephrotic syndrome. Clin J Am Soc Nephrol. 2013 Apr;8(4):637-48. doi: 10.2215/CJN.07200712. Epub 2013 Jan 24.

Meisel C, Sadowski CE, Kohlstedt D, Keller K, Stäritz F, Grübling N, Becker K, Mackenroth L, Rump A, Schröck E, Arnold N, Wimberger P, Kast K. Spectrum of genetic variants of BRCA1 and BRCA2 in a German single center study. Arch Gynecol Obstet. 2017 May;295(5):1227-1238. doi: 10.1007/s00404-017-4330-z. Epub 2017 Mar 21. PMID: 28324225

Mickiewicz A, Chmara M, Futema M, Fijalkowski M, Chlebus K, Galaska R, Bandurski T, Pajkowski M, Zuk M, Wasag B, Limon J, Rynkiewicz A, Gruchala M. Efficacy of clinical diagnostic criteria for familial hypercholesterolemia genetic testing in Poland. Atherosclerosis. 2016 Jun;249:52-8. doi: 10.1016/j.atherosclerosis.2016.03.025. Epub 2016 Mar 26.

Midro AT, Stasiewicz-Jarocka B, Borys J, Hubert E, Skotnicka B, Hassmann-Poznańska E, Sierpińska T, Panasiuk B, Schanze D, Zenker M. Two unrelated families with variable expression of Fraser syndrome due to the same pathogenic variant in the FRAS1 gene. Am J Med Genet A. 2020 Apr;182(4):773-779. doi: 10.1002/ajmg.a.61495. Epub 2020 Jan 30.

Milacic I, Barac M, Milenkovic T, Ugrin M, Klaassen K, Skakic A, Jesic M, Joksic I, Mitrovic K, Todorovic S, Vujovic S, Pavlovic S, Stojiljkovic M. Molecular genetic study of congenital adrenal hyperplasia in Serbia: novel p.Leu129Pro and p.Ser165Pro CYP21A2 gene mutations. J Endocrinol Invest. 2015 Nov;38(11):1199-210. doi: 10.1007/s40618-015-0366-8. Epub 2015 Aug 2.

Milenković T, Koehler K, Krumbholz M, Zivanović S, Zdravković D, Huebner A. Three siblings with triple A syndrome with a novel frameshift mutation in the AAAS gene and a review of 17 independent patients with the frequent p.Ser263Pro mutation. Eur J Pediatr. 2008 Sep;167(9):1049-55. doi: 10.1007/s00431-007-0640-7. Epub 2008 Jan 3.

Milic A, Canki-Klain N. Calpainopathy (LGMD2A) in Croatia: molecular and haplotype analysis. Croat Med J. 2005 Aug;46(4):657-63.

Minarik G, Ferakova E, Ficek A, Polakova H, Kadasi L. GJB2 gene mutations in Slovak hearing-impaired patients of Caucasian origin: spectrum, frequencies and SNP analysis.Clin Genet. 2005 Dec;68(6):554-7. doi: 10.1111/j.1399-0004.2005.00529.x.

Minárik G, Tretinárová D, Szemes T, Kádasi L. Prevalence of DFNB1 mutations in Slovak patients with non-syndromic hearing loss. Int J Pediatr Otorhinolaryngol. 2012 Mar;76(3):400-3. doi: 10.1016/j.ijporl.2011.12.020. Epub 2012 Jan 26.

Miroshnikova VV, Romanova OV, Ivanova ON, Fedyakov MA, Panteleeva AA, Barbitoff YA, Muzalevskaya MV, Urazgildeeva SA, Gurevich VS, Urazov SP, Scherbak SG, Sarana AM, Semenova NA, Anisimova IV, Guseva DM, Pchelina SN, Glotov AS, Zakharova EY, Glotov OS. Identification of novel variants in the LDLR gene in Russian patients with familial hypercholesterolemia using targeted sequencing. Biomed Rep. 2021 Jan;14(1):15. doi: 10.3892/br.2020.1391. Epub 2020 Nov 17.

Mitui M, Bernatowska E, Pietrucha B, Piotrowska-Jastrzebska J, Eng L, Nahas S, Teraoka S, Sholty G, Purayidom A, Concannon P, Gatti RA. ATM gene founder haplotypes and associated mutations in Polish families with ataxia-telangiectasia. Ann Hum Genet. 2005 Nov;69(Pt 6):657-64. doi: 10.1111/j.1529-8817.2005.00199.x

Morak M, Laner A, Bacher U, Keiling C, Holinski-Feder E. MUTYH-associated polyposis - variability of the clinical phenotype in patients with biallelic and monoallelic MUTYH mutations and report on novel mutations. Clin Genet. 2010 Oct;78(4):353-63. doi: 10.1111/j.1399-0004.2010.01478.x.

Murtazina A, Nikitin S, Rudenskaya G, Sharkova I, Borovikov A, Sparber P, Shchagina O, Chukhrova A, Ryzhkova O, Shatokhina O, Orlova A, Udalova V, Kanivets I, Korostelev S, Polyakov A, Dadali E, Kutsev S. Genetic and Clinical Spectrum of GNE Myopathy in Russia. Genes (Basel). 2022 Oct 31;13(11):1991. doi: 10.3390/genes13111991.

Müller CR, Fregin A, Srsen S, Srsnova K, Halliger-Keller B, Felbor U, Seemanova E, Kress W. Allelic heterogeneity of alkaptonuria in Central Europe. Eur J Hum Genet. 1999 Sep;7(6):645-51. doi: 10.1038/sj.ejhg.5200343.

Nedoszytko B, Siemińska A, Strapagiel D, Dąbrowski S, Słomka M, Sobalska-Kwapis M, Marciniak B, Wierzba J, Skokowski J, Fijałkowski M, Nowicki R, Kalinowski L. High prevalence of carriers of variant c.1528G>C of HADHA gene causing long-chain 3-hydroxyacyl-CoA dehydrogenase deficiency (LCHADD) in the population of adult Kashubians from North Poland. PLoS One. 2017 Nov 2;12(11):e0187365. doi: 10.1371/journal.pone.0187365. eCollection 2017.

Nizic-Kos T, Krajc M, Blatnik A, Stegel V, Skerl P, Novakovic S, Gazic B, Besic N. Bilateral Disease Common Among Slovenian CHEK2-Positive Breast Cancer Patients.Ann Surg Oncol. 2020 Oct 8. doi: 10.1245/s10434-020-09178-y. Online ahead of print.

Noskowicz M, Bogdanova N, Bermisheva M, Takhirova Z, Antonenkova N, Khusnutdinova E, Bremer M, Christiansen H, Park-Simon TW, Hillemanns P, Dörk T. Prevalence of PALB2 mutation c.509_510delGA in unselected breast cancer patients from Central and Eastern Europe. Fam Cancer. 2014 Jun;13(2):137-42. doi: 10.1007/s10689-013-9684-1.

Nowinska AK, Wylegala E, Teper S, Wróblewska-Czajka E, Aragona P, Roszkowska AM, Micali A, Pisani A, Puzzolo D. Phenotype and genotype analysis in patients with macular corneal dystrophy. Br J Ophthalmol. 2014 Nov;98(11):1514-21. doi: 10.1136/bjophthalmol-2014-305098. Epub 2014 Jun 11.

Nurmi A, Muranen TA, Pelttari LM, Kiiski JI, Heikkinen T, Lehto S, Kallioniemi A, Schleutker J, Bützow R, Blomqvist C, Aittomäki K, Nevanlinna H. Recurrent moderate-risk mutations in Finnish breast and ovarian cancer patients. Int J Cancer. 2019 Nov 15;145(10):2692-2700. doi: 10.1002/ijc.32309. Epub 2019 Apr 25.

Obermannova B, Pfaeffle R, Zygmunt-Gorska A, Starzyk J, Verkauskiene R, Smetanina N, Bezlepkina O, Peterkova V, Frisch H, Cinek O, Child CJ, Blum WF, Lebl J. Mutations and pituitary morphology in a series of 82 patients with PROP1 gene defects. Horm Res Paediatr. 2011;76(5):348-54. doi: 10.1159/000332693. Epub 2011 Oct 18.

Orlova EM, Bukina AM, Kuznetsova ES, Kareva MA, Zakharova EU, Peterkova VA, Dedov II. Autoimmune polyglandular syndrome type 1 in Russian patients: clinical variants and autoimmune regulator mutations. Horm Res Paediatr. 2010;73(6):449-57. doi: 10.1159/000313585. Epub 2010 Apr 20.

Ostrozovicova M, Jech R, Steel D, Pavelekova P, Han V, Gdovinova Z, Lichtner P, Kurian MA, Wiethoff S, Houlden H, Havránková P, Winkelmann J, Zech M, Skorvanek M. A Recurrent VPS16 p.Arg187* Nonsense Variant in Early-Onset Generalized Dystonia. Mov Disord. 2021 Aug;36(8):1984-1985. doi: 10.1002/mds.28647. Epub 2021 May 17.

Özbay Hoşnut F, Karadağ Öncel E, Öncel MY, Özcay F. A Turkish case of congenital chloride diarrhea with SLC26A3 gene (c.2025_2026insATC) mutation: diagnostic pitfalls. Turk J Gastroenterol. 2010 Dec;21(4):443-7.

Palacios L, Grandoso L, Cuevas N, Olano-Martín E, Martinez A, Tejedor D, Stef M. Molecular characterization of familial hypercholesterolemia in Spain. Atherosclerosis. 2012 Mar;221(1):137-42. doi: 10.1016/j.atherosclerosis.2011.12.021. Epub 2011 Dec 23.

Palmero EI, Carraro DM, Alemar B, Moreira MAM, Ribeiro-Dos-Santos Â, Abe-Sandes K, Galvão HCR, Reis RM, de Pádua Souza C, Campacci N, Achatz MI, Brianese RC, da Cruz Formiga MN, Makdissi FB, Vargas FR, Evangelista Dos Santos AC, Seuanez HN, Lobo de Souza KR, Netto CBO, Santos-Silva P, da Silva GS, Burbano RMR, Santos S, Assumpção PP, Bernardes IMM, Machado-Lopes TMB, Bomfim TF, Toralles MBP, Nascimento I, Garicochea B, Simon SD, Noronha S, de Lima FT, Chami AM, Bittar CM, Bines J, Artigalas O, Esteves-Diz MDP, Lajus TBP, Gifoni ACLVC, Guindalini RSC, Cintra TS, Schwartz IVD, Bernardi P, Miguel D, Nogueira STDS, Herzog J, Weitzel JN, Ashton-Prolla P. The germline mutational landscape of BRCA1 and BRCA2 in Brazil. Sci Rep. 2018 Jun 15;8(1):9188. doi: 10.1038/s41598-018-27315-2.

Palomba G, Cossu A, Friedman E, Budroni M, Farris A, Contu A, Pisano M, Baldinu P, Sini MC, Tanda F, Palmieri G. Origin and distribution of the BRCA2-8765delAG mutation in breast cancer. BMC Cancer. 2007 Jul 19;7:132. doi: 10.1186/1471-2407-7-132.

Pampukha V, Nechyporenko M, Livshyts L. Analysis of EX5del4232ins268 and EX5del955 PAH gene mutations in Ukrainian patients with phenylketonuria. Genes Dis. 2016 Dec 14;4(2):108-110. doi: 10.1016/j.gendis.2016.11.004. eCollection 2017 Jun. Genes Dis. 2016 Dec 14;4(2):108-110. doi: 10.1016/j.gendis.2016.11.004. eCollection 2017 Jun.

Pampukha VM, Kravchenko SA, Tereshchenko FA, Livshits LA, Drozhyna GI. Novel L558P mutation of the gene found in Ukrainian families with atypical corneal dystrophy. Ophthalmologica. 2009;223(3):207-14. doi: 10.1159/000202645. Epub 2009 Feb 17.

Papp J, Kovacs ME, Matrai Z, Orosz E, Kásler M, Børresen-Dale AL, Olah E. Contribution of APC and MUTYH mutations to familial adenomatous polyposis susceptibility in Hungary. Fam Cancer. 2016 Jan;15(1):85-97. doi: 10.1007/s10689-015-9845-5.

Pasic S, Vujic D, Veljković D, Slavkovic B, Mostarica-Stojkovic M, Minic P, Minic A, Ristic G, Giliani S, Villa A, Sobacchi C, Lilić D, Abinun M. Severe combined immunodeficiency in Serbia and Montenegro between years 1986 and 2010: a single-center experience. J Clin Immunol. 2014 Apr;34(3):304-8. doi: 10.1007/s10875-014-9991-9. Epub 2014 Feb 1.

Peco-Antić A, Konrad M, Milosevski-Lomić G, Dimitrijević N. Familial hypomagnesaemia with hypercalciuria and nephrocalcinosis: the first four patients in Serbia. Srp Arh Celok Lek. 2010 May-Jun;138(5-6):351-5. doi: 10.2298/sarh1006351p.

Peczkowska M, Erlic Z, Hoffmann MM, Furmanek M, Cwikla J, Kubaszek A, Prejbisz A, Szutkowski Z, Kawecki A, Chojnowski K, Lewczuk A, Litwin M, Szyfter W, Walter MA, Sullivan M, Eng C, Januszewicz A, Neumann HP. Impact of screening kindreds for SDHD p.Cys11X as a common mutation associated with paraganglioma syndrome type 1. J Clin Endocrinol Metab. 2008 Dec;93(12):4818-25. doi: 10.1210/jc.2008-1290. Epub 2008 Sep 30.

Peric S, Stevanovic J, Johnson K, Kosac A, Peric M, Brankovic M, Marjanovic A, Jankovic M, Banko B, Milenkovic S, Durdic M, Bozovic I, Glumac JN, Lavrnic D, Maksimovic R, Milic-Rasic V, Rakocevic-Stojanovic V. Phenotypic and genetic spectrum of patients with limb-girdle muscular dystrophy type 2A from Serbia. Acta Myol. 2019 Sep 1;38(3):163-171. eCollection 2019 Sep.

Peric S, Glumac JN, Töpf A, Savić-Pavićević D, Phillips L, Johnson K, Cassop-Thompson M, Xu L, Bertoli M, Lek M, MacArthur D, Brkušanin M, Milenković S, Rašić VM, Banko B, Maksimović R, Lochmüller H, Stojanović VR, Straub V. A novel recessive TTN founder variant is a common cause of distal myopathy in the Serbian population. Eur J Hum Genet. 2017 May;25(5):572-581. doi: 10.1038/ejhg.2017.16. Epub 2017 Mar 15.

Perko D, Debeljak M, Toplak N, Avčin T. Clinical features and genetic background of the periodic Fever syndrome with aphthous stomatitis, pharyngitis, and adenitis: a single center longitudinal study of 81 patients. Mediators Inflamm. 2015;2015:293417. doi: 10.1155/2015/293417. Epub 2015 Mar 4.

Pichkur N.O., Olkhovych N.V., Doronina Ya.I. Classic galactosemia: features of diagnosis and treatment Zdorov'e Rebenka. 2018;13(1):48-58. doi: 10.22141/2224-0551.13.1.2018.127066 [Article in Ukrainian]

Piekutowska-Abramczuk D, Kaliszewska M, Sułek A, Jurkowska N, Ołtarzewski M, Jabłońska E, Trubicka J, Głowacka A, Ciara E, Kowalski P, Langiewicz-Wojciechowska K, Tesarova M, Zeman J, Kierdaszuk B, Kuczyński D, Chmielewski D, Szymańska E, Bakuła A, Łusakowska A, Lipowska M, Brodacki B, Pera J, Dorobek M, Rydzanicz M, Płoski R, Chrzanowska KH, Bartnik E, Placha G, Kamińska A, Kostera-Pruszczyk A, Krajewska-Walasek M,

Tońska K, Pronicka E. The frequency of mitochondrial polymerase gamma related disorders in a large Polish population cohort. Mitochondrion. 2019 Jul;47:179-187. doi: 10.1016/j.mito.2018.11.004. Epub 2018 Nov 10.

Piekutowska-Abramczuk D, Olsen RK, Wierzba J, Popowska E, Jurkiewicz D, Ciara E, Ołtarzewski M, Gradowska W, Sykut-Cegielska J, Krajewska-Walasek M, Andresen BS, Gregersen N, Pronicka E. A comprehensive HADHA c.1528G>C frequency study reveals high prevalence of long-chain 3-hydroxyacyl-CoA dehydrogenase deficiency in Poland. J Inherit Metab Dis. 2010 Dec;33 Suppl 3:S373-7. doi: 10.1007/s10545-010-9190-7. Epub 2010 Sep 3.

Piekutowska-Abramczuk D, Pronicki M, Strawa K, Karkucińska-Więckowska A, Szymańska-Dębińska T, Fidziańska A, Więckowski MR, Jurkiewicz D, Ciara E, Jankowska I, Sykut-Cegielska J, Krajewska-Walasek M, Płoski R, Pronicka E. Novel c.191C>G (p.Pro64Arg) MPV17 mutation identified in two pairs of unrelated Polish siblings with mitochondrial hepatoencephalopathy. Clin Genet. 2014 Jun;85(6):573-7. doi: 10.1111/cge.12228. Epub 2013 Jul 28

Pigg MH, Bygum A, Gånemo A, Virtanen M, Brandrup F, Zimmer AD, Hotz A, Vahlquist A, Fischer J. Spectrum of Autosomal Recessive Congenital Ichthyosis in Scandinavia: Clinical Characteristics and Novel and Recurrent Mutations in 132 Patients. Acta Derm Venereol. 2016 Nov 2;96(7):932-937. doi: 10.2340/00015555-2418.

Pirillo A, Garlaschelli K, Arca M, Averna M, Bertolini S, Calandra S, Tarugi P, Catapano AL; LIPIGEN Group. Spectrum of mutations in Italian patients with familial hypercholesterolemia: New results from the LIPIGEN study. Atheroscler Suppl. 2017 Oct;29:17-24. doi: 10.1016/j.atherosclerosissup.2017.07.002.

Platonov AE, Beloborodov VB, Vershinina IV. Meningococcal disease in patients with late complement component deficiency: studies in the U.S.S.R. Medicine (Baltimore). 1993 Nov;72(6):374-92. doi: 10.1097/00005792-199311000-00002.

Plonis J, Kalniete D, Nakazawa-Miklasevica M, Irmejs A, Vjaters E, Gardovskis J, Miklasevics E. The CHEK2 del5395 is a founder mutation without direct effects for cancer risk in the latvian population. Balkan J Med Genet. 2016 Jul 9;18(2):33-36. doi: 10.1515/bjmg-2015-0083. eCollection 2015 Dec 1.

Podkrajsek KT, Bratanic N, Krzisnik C, Battelino T. Autoimmune regulator-1 messenger ribonucleic acid analysis in a novel intronic mutation and two additional novel AIRE gene mutations in a cohort of autoimmune polyendocrinopathy-candidiasis-ectodermal dystrophy patients. J Clin Endocrinol Metab. 2005 Aug;90(8):4930-5. doi: 10.1210/jc.2005-0418. Epub 2005 May 10.

Podralska MJ, Stembalska A, Ślęzak R, Lewandowicz-Uszyńska A, Pietrucha B, Kołtan S, Wigowska-Sowińska J, Pilch J, Mosor M, Ziółkowska-Suchanek I, Dzikiewicz-Krawczyk A, Słomski R. Ten new ATM alterations in Polish patients with ataxia-telangiectasia. Mol Genet Genomic Med. 2014 Nov;2(6):504-11. doi: 10.1002/mgg3.98. Epub 2014 Jul 30.

Pogoda TV, Krakhmaleva IN, Lipatova NA, Shakhovskaya NI, Shishkin SS, Limborska SA. High incidence of 550delA mutation of CAPN3 in LGMD2 patients from Russia.Hum Mutat. 2000 Mar;15(3):295. doi: 10.1002/(SICI)1098-1004(200003)15:3<295::AID-HUMU15>3.0.CO;2-8.

Pokora P, Jezela-Stanek A, Różdżyńska-Świątkowska A, Jurkiewicz E, Bogdańska A, Szymańska E, Rokicki D, Ciara E, Rydzanicz M, Stawiński P, Płoski R, Tylki-Szymańska A. Mild phenotype of glutaric aciduria type 1 in polish patients - novel data from a group of 13 cases. Metab Brain Dis. 2019 Apr;34(2):641-649. doi: 10.1007/s11011-018-0357-5. Epub 2018 Dec 20.

Polak E, Ficek A, Radvanszky J, Soltysova A, Urge O, Cmelova E, Kantarska D, Kadasi L. Phenylalanine hydroxylase deficiency in the Slovak population: genotype-phenotype correlations and genotype-based predictions of BH4-responsiveness. Gene. 2013 Sep 10;526(2):347-55. doi: 10.1016/j.gene.2013.05.057. Epub 2013 Jun 10.

Poláková H, Katrincsáková B, Minárik G, Feráková E, Ficek A, Baldovic M, Kádasi L. Detection of His1069Gln mutation in Wilson disease by bidirectional PCR amplification of specific alleles (BI-PASA) test. Gen Physiol Biophys. 2007 Jun;26(2):91-6.

Popova DP, Kaneva R, Varbanova S, Popov TM. Prevalence of GBJ2 mutations in patients with severe to profound congenital nonsyndromic sensorineural hearing loss in Bulgarian population. Eur Arch Otorhinolaryngol. 2012 Jun;269(6):1589-92. doi: 10.1007/s00405-011-1817-2. Epub 2011 Oct 29.

Popovska-Jankovic K, Tasic V, Bogdanovic R, Miljkovic P, Golubovic E, Soylu A, Saraga M, Pavicevic S, Baskin E, Akil I, Gregoric A, Lilova M, Topaloglu R, Sukarova Stefanovska E, Plaseska-Karanfilska D. Molecular characterization of cystinuria in south-eastern European countries. Urolithiasis. 2013 Feb;41(1):21-30. doi: 10.1007/s00240-012-0531-x. Epub 2012 Dec 27.

Prokofyeva D, Bogdanova N, Dubrowinskaja N, Bermisheva M, Takhirova Z, Antonenkova N, Turmanov N, Datsyuk I, Gantsev S, Christiansen H, Park-Simon TW, Hillemanns P, Khusnutdinova E, Dörk T. Nonsense mutation p.Q548X in BLM, the gene mutated in Bloom's syndrome, is associated with breast cancer in Slavic populations. Breast Cancer Res Treat. 2013 Jan;137(2):533-9. doi: 10.1007/s10549-012-2357-1. Epub 2012 Dec 6.

Pronicka E, Ciara E, Halat P, Janiec A, Wójcik M, Rowińska E, Rokicki D, Płudowski P, Wojciechowska E, Wierzbicka A, Książyk JB, Jacoszek A, Konrad M, Schlingmann KP, Litwin M. Biallelic mutations in CYP24A1 or SLC34A1 as a cause of infantile idiopathic hypercalcemia (IIH) with vitamin D hypersensitivity: molecular study of 11 historical IIH cases. J Appl Genet. 2017 Aug;58(3):349-353. doi: 10.1007/s13353-017-0397-2. Epub 2017 May 3.

Pronicka E, Sykut-Cegielska J. [Orphanet Polska – w europejskiej sieci jako szansa oceny sytuacji chorób rzadkich na przykładzie wrodzonych wad metabolizmu u dzieci]. Pediatria Polska, 2008, V.83 (6); 704-711

Pronicka E, Węglewska-Jurkiewicz A, Taybert J, Pronicki M, Szymańska-Dębińska T, Karkucińska-Więckowska A, Jakóbkiewicz-Banecka J, Kowalski P, Piek+utowska-Abramczuk D, Pajdowska M, Socha P, Sykut-Cegielska J, Węgrzyn G. Post mortem identification of deoxyguanosine kinase (DGUOK) gene mutations combined with impaired glucose homeostasis and iron overload features in four infants with severe progressive liver failure. J Appl Genet. 2011 Feb;52(1):61-6. doi: 10.1007/s13353-010-0008-y. Epub 2010 Nov 16.

Pronicki M, Kowalski P, Piekutowska-Abramczuk D, Taybert J, Karkucinska-Wieckowska A, Szymanska-Debinska T, Karczmarewicz E, Pajdowska M, Migdal M, Milewska-Bobula B, Sykut-Cegielska J, Popowska E. A homozygous mutation in the SCO2 gene causes a spinal muscular atrophy like presentation with stridor and respiratory insufficiency. Eur J Paediatr Neurol. 2010 May;14(3):253-60. doi: 10.1016/j.ejpn.2009.09.008. Epub 2009 Oct 29.

Ramadža DP, Sarnavka V, Vuković J, Fumić K, Krželj V, Lozić B, Pušeljić S, Pereira H, Silva MJ, Tavares de Almeida I, Barić I, Rivera I. Molecular basis and clinical presentation of classic galactosemia in a Croatian population. J Pediatr Endocrinol Metab. 2018 Jan 26;31(1):71-75. doi: 10.1515/jpem-2017-0302.

Ratajska M, Antoszewska E, Piskorz A, Brozek I, Borg Å, Kusmierek H, Biernat W, Limon J. Cancer predisposing BARD1 mutations in breast-ovarian cancer families. Breast Cancer Res Treat. 2012 Jan;131(1):89-97. doi: 10.1007/s10549-011-1403-8. Epub 2011 Feb 23.

Ratajska M, Brozek I, Senkus-Konefka E, Jassem J, Stepnowska M, Palomba G, Pisano M, Casula M, Palmieri G, Borg A, Limon J. BRCA1 and BRCA2 point mutations and large rearrangements in breast and ovarian cancer families in Northern Poland. Oncol Rep. 2008 Jan;19(1):263-8.

Ratajska M, Krygier M, Stukan M, Kuźniacka A, Koczkowska M, Dudziak M, Śniadecki M, Dębniak J, Wydra D, Brozek I, Biernat W, Borg A, Limon J, Wasąg B. Mutational analysis of BRCA1/2 in a group of 134 consecutive ovarian cancer patients. Novel and recurrent BRCA1/2 alterations detected by next generation sequencing. J Appl Genet. 2015 May;56(2):193-8. doi: 10.1007/s13353-014-0254-5. Epub 2014 Nov 1.

Ravnik-Glavac M, Glavac D, Komel R, Dean M. Hum Mutat. Single-stranded conformation polymorphism analysis of the CFTR gene in Slovenian cystic fibrosis patients: detection of mutations and sequence variations. 1993;2(4):286-92. doi: 10.1002/humu.1380020408.

Rebbeck TR, Friebel TM, Friedman E, et al. Mutational spectrum in a worldwide study of 29,700 families with BRCA1 or BRCA2 mutations. Hum Mutat. 2018 May;39(5):593-620. doi: 10.1002/humu.23406. Epub 2018 Mar 12.

Réblová K, Hrubá Z, Procházková D, Pazdírková R, Pouchlá S, Zeman J, Fajkusová L. Hyperphenylalaninemia in the Czech Republic: genotype-phenotype correlations and in silico analysis of novel missense mutations. Clin Chim Acta. 2013 Apr 18;419:1-10. doi: 10.1016/j.cca.2013.01.006. Epub 2013 Jan 26.

Richard I, Roudaut C, Saenz A, Pogue R, Grimbergen JE, Anderson LV, Beley C, Cobo AM, de Diego C, Eymard B, Gallano P, Ginjaar HB, Lasa A, Pollitt C, Topaloglu H, Urtizberea JA, de Visser M, van der Kooi A, Bushby K, Bakker E, Lopez de Munain A, Fardeau M, Beckmann JS. Calpainopathy-a survey of mutations and polymorphisms. Am J Hum Genet. 1999 Jun;64(6):1524-40. doi: 10.1086/302426.

Rudaitis V, Mikliusas V, Januska G, Jukna P, Mickys U, Janavicius R. The incidence of occult ovarian neoplasia and cancer in BRCA1/2 mutation carriers after the bilateral prophylactic salpingo-oophorectomy (PBSO): A single-center prospective study. Eur J Obstet Gynecol Reprod Biol. 2020 Apr;247:26-31. doi: 10.1016/j.ejogrb.2020.01.040. Epub 2020 Jan 30.

Rukavina AS, Topić RZ, Ferencak G, Sucic M. A novel missense mutation C127R (FH Zagreb) in the LDL-receptor gene. Clin Chem Lab Med. 2001 Jun;39(6):505-8. doi: 10.1515/CCLM.2001.084

Rusak B, Kluźniak W, Wokołorczyk D, Stempa K, Kashyap A, Rudnicka H, Gronwald J, Huzarski T, Dębniak T, Jakubowska A, Szwiec M, Akbari MR, Narod SA, Lubiński J, Cybulski C; Polish Hereditary Breast Cancer Consortium. Allelic modification of breast cancer risk in women with an NBN mutation. Breast Cancer Res Treat. 2019 Nov;178(2):427-431. doi: 10.1007/s10549-019-05391-w. Epub 2019 Aug 13.

Salomashkina VV, Pshenichnikova OS, Perina FG, Surin VL. A founder effect in hemophilia A patients from Russian Ural region with a new p.(His634Arg) variant in F8 gene. Blood Coagul Fibrinolysis. 2022 Mar 1;33(2):124-129. doi: 10.1097/MBC.0000000000001073.

Saucedo L, Ackermann L, Platonov AE, Gewurz A, Rakita RM, Densen P. Delineation of additional genetic bases for C8 beta deficiency. Prevalence of null alleles and predominance of C-->T transition in their genesis. J Immunol. 1995 Nov 15;155(10):5022-8.

Savostyanov K, Murashkin N, Pushkov A, Zhanin I, Suleymanov E, Akhkiamova M, Shchagina O, Balanovska E, Epishev R, Polyakov A, Fisenko A. Targeted NGS in Diagnostics of Genodermatosis Characterized by the Epidermolysis Bullosa Symptom Complex in 268 Russian Children. Int J Mol Sci. 2022 Nov 18;23(22):14343. doi: 10.3390/ijms232214343.

Schlingmann KP, Kaufmann M, Weber S, Irwin A, Goos C, John U, Misselwitz J, Klaus G, Kuwertz-Bröking E, Fehrenbach H, Wingen AM, Güran T, Hoenderop JG, Bindels RJ, Prosser DE, Jones G, Konrad M. Mutations in CYP24A1 and idiopathic infantile hypercalcemia. N Engl J Med. 2011 Aug 4;365(5):410-21. doi: 10.1056/NEJMoa1103864. Epub 2011 Jun 15.

Schneppenheim R, Kremer Hovinga JA, Becker T, Budde U, Karpman D, Brockhaus W, Hrachovinová I, Korczowski B, Oyen F, Rittich S, von Rosen J, Tjønnfjord GE, Pimanda JE, Wienker TF, Lämmle B. A common origin of the 4143insA ADAMTS13 mutation. Thromb Haemost. 2006 Jul;96(1):3-6. doi: 10.1160/TH05-12-0817.

Ścieżyńska A, Oziębło D, Ambroziak AM, Korwin M, Szulborski K, Krawczyński M, Stawiński P, Szaflik J, Szaflik JP, Płoski R, Ołdak M. Next-generation sequencing of ABCA4: High frequency of complex alleles and novel mutations in patients with retinal dystrophies from Central Europe.Exp Eye Res. 2016 Apr;145:93-99. doi: 10.1016/j.exer.2015.11.011. Epub 2015 Nov 22.

Ścieżyńska A, Ruszkowska E, Szulborski K, Rydz K, Wierzbowska J, Kosińska J, Rękas M, Płoski R, Szaflik JP, Ołdak M. Processing of OPA1 with a novel N-terminal mutation in patients with autosomal dominant optic atrophy: Escape from nonsense-mediated decay. PLoS One. 2017 Aug 25;12(8):e0183866. doi: 10.1371/journal.pone.0183866. eCollection 2017.

Scott SSO, Pedroso JL, Barsottini OGP, França-Junior MC, Braga-Neto P. Natural history and epidemiology of the spinocerebellar ataxias: Insights from the first description to nowadays. J Neurol Sci. 2020 Oct 15;417:117082. doi: 10.1016/j.jns.2020.117082. Epub 2020 Aug 6.

Seeman P, Gebertová K, Paderová K, Sperling K, Seemanová E. Nijmegen breakage syndrome in 13% of age-matched Czech children with primary microcephaly. Pediatr Neurol. 2004 Mar;30(3):195-200. doi: 10.1016/j.pediatrneurol.2003.07.003.

Seemanova E, Varon R, Vejvalka J, Jarolim P, Seeman P, Chrzanowska KH, Digweed M, Resnick I, Kremensky I, Saar K, Hoffmann K, Dutrannoy V, Karbasiyan M, Ghani M, Barić I, Tekin M, Kovacs P, Krawczak M, Reis A, Sperling K, Nothnagel M. The Slavic NBN Founder Mutation: A Role for Reproductive Fitness? PLoS One. 2016 Dec 9;11(12):e0167984. doi: 10.1371/journal.pone.0167984. eCollection 2016.

Semenova N, Marakhonov A, Ampleeva M, Kurkina M, Baydakova G, Skoblov M, Taran N, Babak O, Shchukina E, Strokova T. Hyperammonemia in Russia Due to Carbonic Anhydrase VA Deficiency Caused by Homozygous Mutation p.Lys185Lys (c.555G>A) of the CA5A Gene. Int J Mol Sci. 2022 Nov 30;23(23):15026. doi: 10.3390/ijms232315026.

Semenova AE, Sergienko IV, García-Giustiniani D, Monserrat L, Popova AB, Nozadze DN, Ezhov MV. Verification of Underlying Genetic Cause in a Cohort of Russian Patients with Familial Hypercholesterolemia Using Targeted Next Generation Sequencing. J Cardiovasc Dev Dis. 2020 May 14;7(2):16. doi: 10.3390/jcdd7020016.

Sepp R, Hategan L, Csányi B, Borbás J, Tringer A, Pálinkás ED, Nagy V, Takács H, Latinovics D, Nyolczas N, Pálinkás A, Faludi R, Rábai M, Szabó GT, Czuriga D, Balogh L, Halmosi R, Borbély A, Habon T, Hegedűs Z, Nagy I. The Genetic Architecture of Hypertrophic Cardiomyopathy in Hungary: Analysis of 242 Patients with a Panel of 98 Genes. Diagnostics (Basel). 2022 May 3;12(5):1132. doi: 10.3390/diagnostics12051132.

Shagam L.I. Semiconductor sequencing in the diagnosis of Alport syndrome (Dissertation, in Russian), Pirogov Medical University, Moscow, 2017.

Shagina OA, Dadali EL, Fedotov VP, Tiburkova TB, Poliakov AV. [Hereditary motor and sensory neuropathy type 4A]. Zh Nevrol Psikhiatr Im S S Korsakova. 2010;110(5 Pt 1):13-6.

Sharapova SO, Guryanova IE, Pashchenko OE, Kondratenko IV, Kostyuchenko LV, Rodina YA, Varlamova TV, Bondarenko AV, Chernyshova LI, Gyseva MN, Belevtsev MV, Minakovskaya NV, Aleinikova OV. Molecular Characteristics, Clinical and Immunologic Manifestations of 11 Children with Omenn Syndrome in East Slavs (Russia, Belarus, Ukraine). J Clin Immunol. 2016 Jan;36(1):46-55. doi: 10.1007/s10875-015-0216-7. Epub 2015 Nov 23.

Sharapova SO, Skomska-Pawliszak M, Rodina YA, Wolska-Kuśnierz B, Dabrowska-Leonik N, Mikołuć B, Pashchenko OE, Pasic S, Freiberger T, Milota T, Formánková R, Szaflarska A, Siedlar M, Avčin T, Markelj G, Ciznar P, Kalwak K, Kołtan S, Jackowska T, Drabko K, Gagro A, Pac M, Naumova E, Kandilarova S, Babol-Pokora K, Varabyou DS, Barendregt BH, Raykina EV, Varlamova TV, Pavlova AV, Grombirikova H, Debeljak M, Mersiyanova IV, Bondarenko AV, Chernyshova LI, Kostyuchenko LV, Guseva MN, Rascon J, Muleviciene A, Preiksaitiene E, Geier CB, Leiss-Piller A, Yamazaki Y, Kawai T, Walter JE, Kondratenko IV, Šedivá A, van der Burg M, Kuzmenko NB, Notarangelo LD, Bernatowska E, Aleinikova OV. The Clinical and Genetic Spectrum of 82 Patients With RAG Deficiency Including a c.256_257delAA Founder Variant in Slavic Countries. Front Immunol. 2020 Jun 10;11:900. doi: 10.3389/fimmu.2020.00900. eCollection 2020.

Shchagina OA, Milovidova TB, Murtazina AF, Rudenskaya GE, Nikitin SS, Dadali EL, Polyakov AV. HINT1 gene pathogenic variants: the most common cause of recessive hereditary motor and sensory neuropathies in Russian patients. Mol Biol Rep. 2020 Feb;47(2):1331-1337. doi: 10.1007/s11033-019-05238-z. Epub 2019 Dec 17.

Shen L, Cong X, Zhang X, Wang N, Zhou P, Xu Y, Zhu Q, Gu X. Clinical and genetic characterization of Chinese pediatric cystine stone patients. J Pediatr Urol. 2017 Dec;13(6):629.e1-629.e5. doi: 10.1016/j.jpurol.2017.05.021. Epub 2017 Jun 24.

Shilkina O.S., Shnayder N.A., Artyukhov I.P. et al. Problems of differential diagnosis of myoclonus-epilepsy associated with the mutation of the POLG gene and juvenile myoclonic epilepsy: a clinical case. Russkiy zhurnal detskoy nevrologii = Russian Journal of Child Neurology 2018;13(1):57–63. [In Russian]

Shubin V.P., Loginova A.N., Pospekhova N.I., Volkova O.V., Meltsaev G.G., Karpukhin A.V. Spectrum peculiarities and BRCA1 mutation frequency among ovarian cancer patients in Mordovija [In Russian] Medical Genetics 2011, 10 № 1(103). 19-24.

Shulskaya, M. V, Shadrina, M.I., Bakilina, N.A., Zolotova, S. V and Slominsky, P.A. (2018) The spectrum of SDHD mutations in Russian patients with head and neck paraganglioma. *International Journal of Neuroscience*, **128**, 1174–1179.

Sikora P, Zaniew M, Haisch L, Pulcer B, Szczepańska M, Moczulska A, Rogowska-Kalisz A, Bieniaś B, Tkaczyk M, Ostalska-Nowicka D, Zachwieja K, Hyla-Klekot L, Schlingmann KP, Konrad M. Retrospective cohort study of familial hypomagnesaemia with hypercalciuria and nephrocalcinosis due to CLDN16 mutations. Nephrol Dial Transplant. 2015 Apr;30(4):636-44. doi: 10.1093/ndt/gfu374. Epub 2014 Dec 3.

Singh J, Thota N, Singh S, Padhi S, Mohan P, Deshwal S, Sur S, Ghosh M, Agarwal A, Sarin R, Ahmed R, Almel S, Chakraborti B, Raina V, DadiReddy PK, Smruti BK, Rajappa S, Dodagoudar C, Aggarwal S, Singhal M, Joshi A, Kumar R, Kumar A, Mishra DK, Arora N, Karaba A, Sankaran S, Katragadda S, Ghosh A, Veeramachaneni V, Hariharan R, Mannan AU. Screening of over 1000 Indian patients with breast and/or ovarian cancer with a multi-gene panel: prevalence of BRCA1/2 and non-BRCA mutations. Breast Cancer Res Treat. 2018 Jul;170(1):189-196. doi: 10.1007/s10549-018-4726-x. Epub 2018 Feb 22.

Skakic A, Djordjevic M, Sarajlija A, Klaassen K, Tosic N, Kecman B, Ugrin M, Spasovski V, Pavlovic S, Stojiljkovic M. Genetic characterization of GSD I in Serbian population revealed unexpectedly high incidence of GSD Ib and 3 novel SLC37A4 variants. Clin Genet. 2018 Feb;93(2):350-355. doi: 10.1111/cge.13093. Epub 2017 Dec 11.

Skopková Z, Hrabincová E, Stástná S, Kozák L, Adam T. Molecular genetic analysis of SLC3A1 and SLC7A9 genes in Czech and Slovak cystinuric patients. Ann Hum Genet. 2005 Sep;69(Pt 5):501-7. doi: 10.1111/j.1529-8817.2005.00185.x.

Slajpah M, Gorinsek B, Berginc G, Vizjak A, Ferluga D, Hvala A, Meglic A, Jaksa I, Furlan P, Gregoric A, Kaplan-Pavlovcic S, Ravnik-Glavac M, Glavac D. Sixteen novel mutations identified in COL4A3, COL4A4, and COL4A5 genes in Slovenian families with Alport syndrome and benign familial hematuria. Kidney Int. 2007 Jun;71(12):1287-95. doi: 10.1038/sj.ki.5002221. Epub 2007 Mar 28.

Snezhkina A, Fedorova M, Kobelyatskaya A, Markova D, Lantsova M, Ikonnikova A, Emelyanova M, Kalinin D, Pudova E, Melnikova N, Dmitriev A, Krasnov G, Pavlov V, Kudryavtseva A. The SDHD:p.H102R Variant Is Frequent in Russian Patients with Head and Neck Paragangliomas and Associated with Loss of 11p15.5 Region and Hypermethylation of H19-DMR. Int J Mol Sci. 2022 Dec 30;24(1):628. doi: 10.3390/ijms24010628.

Sokolenko AP, Iyevleva AG, Preobrazhenskaya EV, Mitiushkina NV, Abysheva SN, Suspitsin EN, Kuligina ESh, Gorodnova TV, Pfeifer W, Togo AV, Turkevich EA, Ivantsov AO, Voskresenskiy DV, Dolmatov GD, Bit-Sava EM, Matsko DE, Semiglazov VF, Fichtner I, Larionov AA, Kuznetsov SG, Antoniou AC, Imyanitov EN. High prevalence and breast cancer predisposing role of the BLM c.1642 C>T (Q548X) mutation in Russia. Int J Cancer. 2012 Jun 15;130(12):2867-73. doi: 10.1002/ijc.26342. Epub 2011 Oct 20.

Sokolenko AP, Sokolova TN, Ni VI, Preobrazhenskaya EV, Iyevleva AG, Aleksakhina SN, Romanko AA, Bessonov AA, Gorodnova TV, Anisimova EI, Savonevich EL, Bizin IV, Stepanov IA, Krivorotko PV, Berlev IV, Belyaev AM, Togo AV, Imyanitov EN. Frequency and spectrum of founder and non-founder BRCA1 and BRCA2 mutations in a large series of Russian breast cancer and ovarian cancer patients. Breast Cancer Res Treat. 2020 Aug 9. doi: 10.1007/s10549-020-05827-8. Online ahead of print.

Soltysova A, Tothova Tarova E, Ficek A, Baldovic M, Polakova H, Kayserova H, Kadasi L. Comprehensive genetic study of cystic fibrosis in Slovak patients in 25 years of genetic diagnostics. Clin Respir J. 2018 Mar;12(3):1197-1206. doi: 10.1111/crj.12651. Epub 2017 Jun 5.

Spahiu L, Sayer JA, Behluli E, Liehr T, Temaj G. Case Report: Identification of likely recurrent CEP290 mutation in a child with Joubert syndrome and cerebello-retinal-renal features. F1000Res. 2023 Mar 31;11:388. doi: 10.12688/f1000research.109628.2. eCollection 2022.

Srsen S, Müller CR, Fregin A, Srsnova K. Alkaptonuria in Slovakia: thirty-two years of research on phenotype and genotype. Mol Genet Metab. 2002 Apr;75(4):353-9. doi: 10.1016/S1096-7192(02)00002-1.

Staninova-Stojovska M, Matevska-Geskovska N, Panovski M, Angelovska B, Mitrevski N, Ristevski M, Jovanovic R, Dimovski AJ. Molecular Basis of Inherited Colorectal Carcinomas in the Macedonian Population: An Update. Balkan J Med Genet. 2019 Dec 21;22(2):5-16. doi: 10.2478/bjmg-2019-0027. eCollection 2019 Dec.

Stavber L, Hovnik T, Kotnik P, Lovrečić L, Kovač J, Tesovnik T, Bertok S, Dovč K, Debeljak M, Battelino T, Avbelj Stefanija M. High frequency of pathogenic ACAN variants including an intragenic deletion in selected individuals with short stature. Eur J Endocrinol. 2020 Mar;182(3):243-253. doi: 10.1530/EJE-19-0771.

Stenton SL, Sheremet NL, Catarino CB, Andreeva NA, Assouline Z, Barboni P, Barel O, Berutti R, Bychkov I, Caporali L, Capristo M, Carbonelli M, Cascavilla ML, Charbel Issa P, Freisinger P, Gerber S, Ghezzi D, Graf E, Heidler J, Hempel M, Heon E, Itkis YS, Javasky E, Kaplan J, Kopajtich R, Kornblum C, Kovacs-Nagy R, Krylova TD, Kunz WS, La Morgia C, Lamperti C, Ludwig C, Malacarne PF, Maresca A, Mayr JA, Meisterknecht J, Nevinitsyna TA, Palombo F, Pode-Shakked B, Shmelkova MS, Strom TM, Tagliavini F, Tzadok M, van der Ven AT, Vignal-Clermont C, Wagner M, Zakharova EY, Zhorzholadze NV, Rozet JM, Carelli V, Tsygankova PG, Klopstock T, Wittig I, Prokisch H. Impaired complex I repair causes recessive Leber's hereditary optic neuropathy. J Clin Invest. 2021 Mar 15;131(6):e138267. doi: 10.1172/JCI138267.

Stenton SL, Tesarova M, Sheremet NL, Catarino C, Carelli V, Ciara E, Curry K, Engvall M, Fleming LR, Freisinger P, Iwanicka-Pronicka K, Jurkiewicz E, Klopstock T, Koenig MK, Kolářová H, Kousal B, Krylova T, La Morgia C, Nosková L, Piekutowska-Abramczuk D, Russo SN, Stránecký V, Tóthová I, Träisk F, Prokisch H. DNAJC30 defect: a frequent cause of recessive Leber hereditary optic neuropathy and Leigh syndrome. Brain. 2022 Feb 10:awac052. doi: 10.1093/brain/awac052. Online ahead of print.

Stegel V, Krajc M, Zgajnar J, Teugels E, De Grève J, Hočevar M, Novaković S. The occurrence of germline BRCA1 and BRCA2 sequence alterations in Slovenian population. BMC Med Genet. 2011 Jan 14;12:9. doi: 10.1186/1471-2350-12-9.

Stehlíková K, Skálová D, Zídková J, Haberlová J, Voháňka S, Mazanec R, Mrázová L, Vondráček P, Ošlejšková H, Zámečník J, Honzík T, Zeman J, Magner M, Šišková D, Langová M, Gregor V, Godava M, Smolka V, Fajkusová L. Muscular dystrophies and myopathies: the spectrum of mutated genes in the Czech Republic. Clin Genet. 2017 Mar;91(3):463-469. doi: 10.1111/cge.12839. Epub 2016 Sep 26.

Stolarski B, Pronicka E, Korniszewski L, Pollak A, Kostrzewa G, Rowińska E, Włodarski P, Skórka A, Gremida M, Krajewski P, Ploski R. Molecular background of polyendocrinopathy-candidiasis-ectodermal dystrophy syndrome in a Polish population: novel AIRE mutations and an estimate of disease prevalence. Clin Genet. 2006 Oct;70(4):348-54. doi: 10.1111/j.1399-0004.2006.00690.x.

Sułek-Piatkowska A, Zdzienicka E, Raczyńska-Rakowicz M, Krysa W, Rajkiewicz M, Szirkowiec W, Zaremba J. The occurrence of spinocerebellar ataxias caused by dynamic mutations in Polish patients. Neurol Neurochir Pol. 2010 May-Jun;44(3):238-45. doi: 10.1016/s0028-3843(14)60037-2.

Suspitsin E, Sokolenko A, Bizin I, Tumakova A, Guseva M, Sokolova N, Vakhlyarskaya S, Kondratenko I, Imyanitov E. ATM mutation spectrum in Russian children with ataxia-telangiectasia. Eur J Med Genet. 2020 Jan;63(1):103630. doi: 10.1016/j.ejmg.2019.02.003. Epub 2019 Feb 14.

Susswein LR, Marshall ML, Nusbaum R, Vogel Postula KJ, Weissman SM, Yackowski L, Vaccari EM, Bissonnette J, Booker JK, Cremona ML, Gibellini F, Murphy PD, Pineda-Alvarez DE, Pollevick GD, Xu Z, Richard G, Bale S, Klein RT, Hruska KS, Chung WK. Pathogenic and likely pathogenic variant prevalence among the first 10.000 patients referred for next-generation cancer panel testing. Genet Med. 2016 Aug;18(8):823-32. doi: 10.1038/gim.2015.166. Epub 2015 Dec 17.

Suszynska M, Kluzniak W, Wokolorczyk D, Jakubowska A, Huzarski T, Gronwald J, Debniak T, Szwiec M, Ratajska M, Klonowska K, Narod S, Bogdanova N, Dörk T, Lubinski J, Cybulski C, Kozlowski P. BARD1 is A Low/Moderate Breast Cancer Risk Gene: Evidence Based on An Association Study of the Central European p.Q564X Recurrent Mutation. Cancers (Basel). 2019 May 28;11(6). pii: E740. doi: 10.3390/cancers11060740.

Synková I, Bébarová M, Andršová I, Chmelikova L, Švecová O, Hošek J, Pásek M, Vít P, Valášková I, Gaillyová R, Navrátil R, Novotný T. Long-QT founder variant T309I-Kv7.1 with dominant negative pattern may predispose delayed afterdepolarizations under β-adrenergic stimulation. Sci Rep. 2021 Feb 11;11(1):3573. doi: 10.1038/s41598-021-81670-1.

Szaflarska A, Rutkowska-Zapała M, Kotula M, Gruca A, Grabowska A, Lenart M, Surman M, Trzyna E, Mordel A, Pituch-Noworolska A, Siedlar M. Mutation c.256_257delAA in RAG1 Gene in Polish Children with Severe Combined Immunodeficiency: Diversity of Clinical Manifestations. Arch Immunol Ther Exp (Warsz). 2016 Dec;64(Suppl 1):177-183. doi: 10.1007/s00005-016-0447-1. Epub 2017 Jan 12

Szczałuba K, Mierzewska H, Śmigiel R, Kosińska J, Koppolu A, Biernacka A, Stawiński P, Pollak A, Rydzanicz M, Płoski R. AP4B1-associated hereditary spastic paraplegia: expansion of phenotypic spectrum related to homozygous p.Thr387fs variant. J Appl Genet. 2020 Mar 12. doi: 10.1007/s13353-020-00552-w. [Epub ahead of print]

Szczecinska W, Nesteruk D, Wertheim-Tysarowska K, Greenblatt DT, Baty D, Browne F, Liu L, Ozoemena L, Terron-Kwiatkowski A, McGrath JA, Mellerio JE, Morton J, Woźniak K, Kowalewski C, Has C, Moss C. Under-recognition of acral peeling skin syndrome: 59 new cases with 15 novel mutations. Br J Dermatol. 2014 Nov;171(5):1206-10. doi: 10.1111/bjd.12964. Epub 2014 Oct 20.

Tasic V, Dervisov D, Koceva S, Weber S, Konrad M. Hypomagnesemia with hypercalciuria and nephrocalcinosis: case report and a family study. Pediatr Nephrol. 2005 Jul;20(7):1003-6. doi: 10.1007/s00467-005-1853-5. Epub 2005 Apr 26.

Telatar M, Teraoka S, Wang Z, Chun HH, Liang T, Castellvi-Bel S, Udar N, Borresen-Dale AL, Chessa L, Bernatowska-Matuszkiewicz E, Porras O, Watanabe M, Junker A, Concannon P, Gatti RA. Ataxia-telangiectasia: identification and detection of founder-effect mutations in the ATM gene in ethnic populations.Am J Hum Genet. 1998 Jan;62(1):86-97. doi: 10.1086/301673.

Terzic M, Jakimovska M, Fustik S, Jakovska T, Sukarova-Stefanovska E, Plaseska-Karanfilska D. Cystic Fibrosis Mutation Spectrum in North Macedonia: A Step Toward Personalized Therapy. Balkan J Med Genet. 2019 Aug 28;22(1):35-40. doi: 10.2478/bjmg-2019-0009. eCollection 2019 Jun.

Tighe O, Dunican D, O'Neill C, Bertorelle G, Beattie D, Graham C, Zschocke J, Cali F, Romano V, Hrabincova E, Kozak L, Nechyporenko M, Livshits L, Guldberg P, Jurkowska M, Zekanowski C, Perez B, Desviat LR, Ugarte M, Kucinskas V, Knappskog P, Treacy E, Naughten E, Tyfield L, Byck S,

Scriver CR, Mayne PD, Croke DT. Genetic diversity within the R408W phenylketonuria mutation lineages in Europe. Hum Mutat. 2003 Apr;21(4):387-93. doi: 10.1002/humu.10195.

Tikhonovich YuV, Kolodkina AA, Kylikova KS et al. Idiopatic hypercalcemia in infancy. Problemic endocrinologii, 2017;63(1):51-57. https://doi.org/10.14341/probl201763151-57 (in Russian).

Tiranti V, Hoertnagel K, Carrozzo R, Galimberti C, Munaro M, Granatiero M, Zelante L, Gasparini P, Marzella R, Rocchi M, Bayona-Bafaluy MP, Enriquez JA, Uziel G, Bertini E, Dionisi-Vici C, Franco B, Meitinger T, Zeviani M. Mutations of SURF-1 in Leigh disease associated with cytochrome c oxidase deficiency. Am J Hum Genet. 1998 Dec;63(6):1609-21. doi: 10.1086/302150.

Tirosh I, Yamazaki Y, Frugoni F, Ververs FA, Allenspach EJ, Zhang Y, Burns S, Al-Herz W, Noroski L, Walter JE, Gennery AR, van der Burg M, Notarangelo LD, Lee YN. Recombination activity of human recombination-activating gene 2 (RAG2) mutations and correlation with clinical phenotype. J Allergy Clin Immunol. 2019 Feb;143(2):726-735. doi: 10.1016/j.jaci.2018.04.027. Epub 2018 Jun 18.

Thomasová D, Zelinová M, Libik M, Geryk J, Votýpka P, Rajnochová Bloudíčková S, Krejčí K, Reiterová J, Jančová E, Machová J, Kollárová M, Rychík I, Havrda M, Horáčková M, Putzová M, Šafránek R, Kollár M, Macek M Jr. The most common founder pathogenic variant c.868G > A (p.Val290Met) in the NPHS2 gene in a representative adult Czech cohort with focal segmental glomerulosclerosis is associated with a milder disease and its underdiagnosis in childhood.Front Med (Lausanne). 2023 Dec 19;10:1320054. doi: 10.3389/fmed.2023.1320054. eCollection 2023.

Todorov T, Savov A, Jelev H, Panteleeva E, Konstantinova D, Krustev Z, Mihaylova V, Tournev I, Tankova L, Tzolova N, Kremensky I. Spectrum of mutations in the Wilson disease gene (ATP7B) in the Bulgarian population. Clin Genet. 2005 Nov;68(5):474-6. doi: 10.1111/j.1399-0004.2005.00516.x.

Todorova A, Georgieva B, Tournev I, Todorov T, Bogdanova N, Mitev V, Mueller CR, Kremensky I, Horst J. A large deletion and novel point mutations in the calpain 3 gene (CAPN3) in Bulgarian LGMD2A patients. Neurogenetics. 2007 Aug;8(3):225-9. doi: 10.1007/s10048-007-0083-3. Epub 2007 Feb 23.

Tomanin R, Karageorgos L, Zanetti A, Al-Sayed M, Bailey M, Miller N, Sakuraba H, Hopwood JJ. Mucopolysaccharidosis type VI (MPS VI) and molecular analysis: Review and classification of published variants in the ARSB gene. Hum Mutat. 2018 Dec;39(12):1788-1802. doi: 10.1002/humu.23613. Epub 2018 Sep 17.

Tomasic NL, Piterkova L, Huff C, Bilic E, Yoon D, Miasnikova GY, Sergueeva AI, Niu X, Nekhai S, Gordeuk V, Prchal JT. The phenotype of polycythemia due to Croatian homozygous VHL (571C>G:H191D) mutation is different from that of Chuvash polycythemia (VHL 598C>T:R200W).Haematologica. 2013 Apr;98(4):560-7. doi: 10.3324/haematol.2012.070508. Epub 2013 Feb 12.

Tracewska AM, Kocyła-Karczmarewicz B, Rafalska A, Murawska J, Jakubaszko-Jablonska J, Rydzanicz M, Stawiński P, Ciara E, Khan MI, Henkes A, Hoischen A, Gilissen C, van de Vorst M, Cremers FPM, Płoski R, Chrzanowska KH. Genetic Spectrum of ABCA4-Associated Retinal Degeneration in Poland. Genes (Basel). 2019 Nov 21;10(12):959. doi: 10.3390/genes10120959.

Trizuljak J, Petruchová T, Blaháková I, Vrzalová Z, Hořínová V, Doubková M, Michalka J, Mayer J, Pospíšilová Š, Doubek M. Diagnosis of Bloom Syndrome in a Patient with Short Stature, Recurrence of Malignant Lymphoma, and Consanguineous Origin. Mol Syndromol. 2020 Jun;11(2):73-82. doi: 10.1159/000507006. Epub 2020 Mar 21.

Tsygankova, P., Mikhaĭlova, S., Zakharova, E., Pichkur, N., Il'ina, E., Nikolaeva, E., Rudenskaia, G., Dadali, E., Kolpakchi, L. and Fedoniuk, ID Matiushchenko, G. (2010) [Syndrome Leigh caused by mutations in the SURF1 gene: clinical and molecular-genetic characteristics]. Zh Nevrol Psikhiatr Im S S Korsakova, 110, 25–32.

Tichý L, Freiberger T, Zapletalová P, Soška V, Ravčuková B, Fajkusová L. The molecular basis of familial hypercholesterolemia in the Czech Republic: spectrum of LDLR mutations and genotype-phenotype correlations. Atherosclerosis. 2012 Aug;223(2):401-8. doi: 10.1016/j.atherosclerosis.2012.05.014. Epub 2012 May 23.

Usher JL, Ascher DB, Pires DE, Milan AM, Blundell TL, Ranganath LR. Analysis of HGD Gene Mutations in Patients with Alkaptonuria from the United Kingdom: Identification of Novel Mutations. JIMD Rep. 2015;24:3-11. doi: 10.1007/8904_2014_380. Epub 2015 Feb 15.

van der Graaf A, Avis HJ, Kusters DM, Vissers MN, Hutten BA, Defesche JC, Huijgen R, Fouchier SW, Wijburg FA, Kastelein JJ, Wiegman A. Molecular basis of autosomal dominant hypercholesterolemia: assessment in a large cohort of hypercholesterolemic children. Circulation. 2011 Mar 22;123(11):1167-73. doi: 10.1161/CIRCULATIONAHA.110.979450. Epub 2011 Mar 7.

van der Velden JJ, Jonkman MF, McLean WH, Hamm H, Steijlen PM, van Steensel MA, van Geel M. A recurrent mutation in the TGM5 gene in European patients with acral peeling skin syndrome. J Dermatol Sci. 2012 Jan;65(1):74-6. doi: 10.1016/j.jdermsci.2011.10.002. Epub 2011 Oct 13.

van Dorland HA, Taleghani MM, Sakai K, Friedman KD, George JN, Hrachovinova I, Knöbl PN, von Krogh AS, Schneppenheim R, Aebi-Huber I, Bütikofer L, Largiadèr CR, Cermakova Z, Kokame K, Miyata T, Yagi H, Terrell DR, Vesely SK, Matsumoto M, Lämmle B, Fujimura Y, Kremer Hovinga JA; Hereditary TTP Registry. The International Hereditary Thrombotic Thrombocytopenic Purpura Registry: key findings at enrollment until 2017. Haematologica. 2019 Oct;104(10):2107-2115. doi: 10.3324/haematol.2019.216796. Epub 2019 Feb 21.

Vilboux T, Kayser M, Introne W, Suwannarat P, Bernardini I, Fischer R, O'Brien K, Kleta R, Huizing M, Gahl WA. Mutation spectrum of homogentisic acid oxidase (HGD) in alkaptonuria. Hum Mutat. 2009 Dec;30(12):1611-9. doi: 10.1002/humu.21120.

Vojtková J, Čiljaková M, Jeseňák M, Mišovicová N, Bánovčin P. Bloom syndrome without typical sun-sensitive skin lesions in three Slovak siblings. Int J Dermatol. 2016 Jun;55(6):687-690. doi: 10.1111/ijd.13009. Epub 2015 Sep 4.

Vrabelova S, Letocha O, Borsky M, Kozak L. Mutation analysis of the ATP7B gene and genotype/phenotype correlation in 227 patients with Wilson disease. Mol Genet Metab. 2005 Sep-Oct;86(1-2):277-85. doi: 10.1016/j.ymgme.2005.05.004. Epub 2005 Jun 20.

Vrzalová Z, Hrubá Z, St'ahlová Hrabincová E, Pouchlá S, Votava F, Kolousková S, Fajkusová L. Identification of CYP21A2 mutant alleles in Czech patients with 21-hydroxylase deficiency. Int J Mol Med. 2010 Oct;26(4):595-603. doi: 10.3892/ijmm_00000504.

Vodnjov N, Toplišek J, Maver A, Čuturilo G, Jaklič H, Teran N, Višnjar T, Škrjanec Pušenjak M, Hodžić A, Miljanović O, Peterlin B, Writzl K. A novel splice-site FHOD3 founder variant is a common cause of hypertrophic cardiomyopathy in the population of the Balkans-A cohort study. PLoS One. 2023 Dec 5;18(12):e0294969. doi: 10.1371/journal.pone.0294969. eCollection 2023.

Volkova NI, Davidenko IY, Reshetnikov IB, Brovkina SS. [Allgrove syndrome: how to suspect the problem? Endocrinologists experience]. Probl Endokrinol (Mosk). 2020 Aug 4;66(1):64-69. doi: 10.14341/probl10296. Article in Russian

Wagner TM, Möslinger RA, Muhr D, Langbauer G, Hirtenlehner K, Concin H, Doeller W, Haid A, Lang AH, Mayer P, Ropp E, Kubista E, Amirimani B, Helbich T, Becherer A, Scheiner O, Breiteneder H, Borg A, Devilee P, Oefner P, Zielinski C. BRCA1-related breast cancer in Austrian breast and ovarian cancer families: specific BRCA1 mutations and pathological characteristics. Int J Cancer. 1998 Jul 29;77(3):354-60. doi: 10.1002/(sici)1097-0215(19980729)77:3<354::aid-ijc8>3.0.co;2-n.

Walczak-Sztulpa J, Wawrocka A, Leszczynska B, Mikulska B, Arts HH, Bukowska-Olech E, Daniel M, Krawczynski MR, Latos-Bielenska A, Obersztyn E. Prenatal genetic diagnosis of cranioectodermal dysplasia in a Polish family with compound heterozygous variants in WDR35. Am J Med Genet A. 2020 Oct;182(10):2417-2425. doi: 10.1002/ajmg.a.61785. Epub 2020 Aug 17.

Walsh T, Casadei S, Coats KH, Swisher E, Stray SM, Higgins J, Roach KC, Mandell J, Lee MK, Ciernikova S, Foretova L, Soucek P, King MC. Spectrum of mutations in BRCA1, BRCA2, CHEK2, and TP53 in families at high risk of breast cancer. JAMA. 2006 Mar 22;295(12):1379-88. doi: 10.1001/jama.295.12.1379.

Wang CY, Davoodi-Semiromi A, Huang W, Connor E, Shi JD, She JX. Characterization of mutations in patients with autoimmune polyglandular syndrome type 1 (APS1). Hum Genet. 1998 Dec;103(6):681-5. doi: 10.1007/s004390050891.

Weber S, Schneider L, Peters M, Misselwitz J, Rönnefarth G, Böswald M, Bonzel KE, Seeman T, Suláková T, Kuwertz-Bröking E, Gregoric A, Palcoux JB, Tasic V, Manz F, Schärer K, Seyberth HW, Konrad M. Novel paracellin-1 mutations in 25 families with familial hypomagnesemia with hypercalciuria and nephrocalcinosis. J Am Soc Nephrol. 2001 Sep;12(9):1872-81.

Weronska A, De la Morena-Barrio B, Goldman-Mazur S, De la Morena-Barrio ME, Padilla J, Miñano A, Garrido P, Treliński J, Potaczek DP, Szczepanek A, Undas A, Corral J, Wypasek E. Functional, biochemical, molecular and clinical characterization of antithrombin c.1157T>C (p.Ile386Thr), a recurrent Polish variant with a founder effect. Haematologica. 2023 Oct 1;108(10):2803-2807. doi: 10.3324/haematol.2022.282459.

Wertheim-Tysarowska K, Sobczyńska-Tomaszewska A, Kowalewski C, Kutkowska-Kaźmierczak A, Woźniak K, Niepokój K, Klausegger A, Sypniewska-Jutkiewicz J, Stępień A, Bal J. Novel and recurrent COL7A1 mutation in a Polish population. Eur J Dermatol. 2012 Jan-Feb;22(1):23-8. doi: 10.1684/ejd.2011.1583.

Wesół-Kucharska D, Kaczor M, Pajdowska M, Ehmke Vel Emczyńska-Seliga E, Bogdańska A, Kozłowski D, Piekutowska-Abramczuk D, Ciara E, Rokicki D. Clinical picture and treatment effects in 5 patients with Methylmalonic aciduria related to MMAA mutations. Mol Genet Metab Rep. 2020 Jan 8;22:100559. doi: 10.1016/j.ymgmr.2019.100559. eCollection 2020 Mar.

Wintjens R, Bozon D, Belabbas K, MBou F, Girardet JP, Tounian P, Jolly M, Boccara F, Cohen A, Karsenty A, Dubern B, Carel JC, Azar-Kolakez A, Feillet F, Labarthe F, Gorsky AM, Horovitz A, Tamarindi C, Kieffer P, Lienhardt A, Lascols O, Di Filippo M, Dufernez F. Global molecular analysis and APOE mutations in a cohort of autosomal dominant hypercholesterolemia patients in France. J Lipid Res. 2016 Mar;57(3):482-91. doi: 10.1194/jlr.P055699. Epub 2016 Jan 22.

Witsch-Baumgartner M, Ciara E, Löffler J, Menzel HJ, Seedorf U, Burn J, Gillessen-Kaesbach G, Hoffmann GF, Fitzky BU, Mundy H, Clayton P, Kelley RI, Krajewska-Walasek M, Utermann G. Frequency gradients of DHCR7 mutations in patients with Smith-Lemli-Opitz syndrome in Europe: evidence for different origins of common mutations. Eur J Hum Genet. 2001 Jan;9(1):45-50. doi: 10.1038/sj.ejhg.5200579.

Witsch-Baumgartner M, Schwentner I, Gruber M, Benlian P, Bertranpetit J, Bieth E, Chevy F, Clusellas N, Estivill X, Gasparini G, Giros M, Kelley RI, Krajewska-Walasek M, Menzel J, Miettinen T, Ogorelkova M, Rossi M, Scala I, Schinzel A, Schmidt K, Schönitzer D, Seemanova E, Sperling K, Syrrou M, Talmud PJ, Wollnik B, Krawczak M, Labuda D, Utermann G. Age and origin of major Smith-Lemli-Opitz syndrome (SLOS) mutations in European populations. J Med Genet. 2008 Apr;45(4):200-9. doi: 10.1136/jmg.2007.053520. Epub 2007 Oct 26.

Yanus GA, Akhapkina TA, Iyevleva AG, Kornilov AV, Suspitsin EN, Kuligina ES, Ivantsov AO, Aleksakhina SN, Sokolova TN, Sokolenko AP, Togo AV, Imyanitov EN. The spectrum of Lynch syndrome-associated germ-line mutations in Russia. Eur J Med Genet. 2020 Mar;63(3):103753. doi: 10.1016/j.ejmg.2019.103753. Epub 2019 Sep 3.

Yanus GA, Akhapkina TA, Ivantsov AO, Preobrazhenskaya EV, Aleksakhina SN, Bizin IV, Sokolenko AP, Mitiushkina NV, Kuligina ES, Suspitsin EN, Venina AR, Holmatov MM, Zaitseva OA, Yatsuk OS, Pashkov DV, Belyaev AM, Togo AV, Imyanitov EN, Iyevleva AG. Spectrum of APC and MUTYH germ-line mutations in Russian patients with colorectal malignancies. Clin Genet. 2018 May;93(5):1015-1021. doi: 10.1111/cge.13228. Epub 2018 Mar 9.

Yanus GA, Akhapkina TA, Whitehead AJ, Bizin IV, Iyevleva AG, Kuligina ES, Aleksakhina SN, Anisimova MO, Holmatov MM, Romanko AA, Zaitseva OA, Yatsuk OS, Zagorodnev KA, Matsneva MA, Koloskov AV, Togo AV, Suspitsin EN, Imyanitov EN. Exome-based search for recurrent disease-causing alleles in Russian population. Eur J Med Genet. 2019 Jul;62(7):103656. doi: 10.1016/j.ejmg.2019.04.013. Epub 2019 Apr 24.

Yanus GA, Savonevich EL, Sokolenko AP, Romanko AA, Ni VI, Bakaeva EK, Gorustovich OA, Bizin IV, Imyanitov EN. Founder vs. non-founder BRCA1/2 pathogenic alleles: the analysis of Belarusian breast and ovarian cancer patients and review of other studies on ethnically homogenous populations. Fam Cancer. 2022 May 21. doi: 10.1007/s10689-022-00296-y. Online ahead of print.

Zariwala MA, Leigh MW, Ceppa F, Kennedy MP, Noone PG, Carson JL, Hazucha MJ, Lori A, Horvath J, Olbrich H, Loges NT, Bridoux AM, Pennarun G, Duriez B, Escudier E, Mitchison HM, Chodhari R, Chung EM, Morgan LC, de Iongh RU, Rutland J, Pradal U, Omran H, Amselem S, Knowles MR. Mutations of DNAI1 in primary ciliary dyskinesia: evidence of founder effect in a common mutation. Am J Respir Crit Care Med. 2006 Oct 15;174(8):858-66. doi: 10.1164/rccm.200603-370OC. Epub 2006 Jul 20.

Zakharova EY, Rudenskaya GE. [A new form of hereditary neurodegeneration with brain iron accumulation: clinical and molecular-genetic characteristics]. Zh Nevrol Psikhiatr Im S S Korsakova. 2014;114(1):4-12. Russian. PMID: 24637810.

Zatková A, de Bernabé DB, Poláková H, Zvarík M, Feráková E, Bosák V, Ferák V, Kádasi L, de Córdoba SR. High frequency of alkaptonuria in Slovakia: evidence for the appearance of multiple mutations in HGO involving different mutational hot spots. Am J Hum Genet. 2000 Nov;67(5):1333-9. doi: 10.1016/S0002-9297(07)62964-4. Epub 2000 Oct 2.

Zatkova A, Sedlackova T, Radvansky J, Polakova H, Nemethova M, Aquaron R, Dursun I, Usher JL, Kadasi L. Identification of 11 Novel Homogentisate 1,2 Dioxygenase Variants in Alkaptonuria Patients and Establishment of a Novel LOVD-Based HGD Mutation Database.JIMD Rep. 2012;4:55-65. doi: 10.1007/8904_2011_68. Epub 2011 Oct 20.

Zavodna K, Bujalkova M, Krivulcik T, Alemayehu A, Skorvaga M, Marra G, Fridrichova I, Jiricny J, Bartosova Z. Novel and recurrent germline alterations in the MLH1 and MSH2 genes identified in hereditary nonpolyposis colorectal cancer patients in Slovakia. Neoplasma. 2006;53(4):269-76.

Zekanowski C, Nowacka M, Radomyska B, Cabalska B. Should newborn mutation scanning for hyperphenylalaninaemia and galactosaemia be implemented? A Polish experience. J Med Screen. 2001;8(3):132-6. doi: 10.1136/jms.8.3.132.

Zietkiewicz E, Bukowy-Bieryllo Z, Rabiasz A, Daca-Roszak P, Wojda A, Voelkel K, Rutkiewicz E, Pogorzelski A, Rasteiro M, Witt M. CFAP300: Mutations in Slavic Patients with Primary Ciliary Dyskinesia and a Role in Ciliary Dynein Arms Trafficking. Am J Respir Cell Mol Biol. 2019 Oct;61(4):440-449. doi: 10.1165/rcmb.2018-0260OC.

Ziętkiewicz E, Nitka B, Voelkel K, Skrzypczak U, Bukowy Z, Rutkiewicz E, Humińska K, Przystałowska H, Pogorzelski A, Witt M. Population specificity of the DNAI1 gene mutation spectrum in primary ciliary dyskinesia (PCD). Respir Res. 2010 Dec 8;11:174. doi: 10.1186/1465-9921-11-174.

Ziętkiewicz E, Rutkiewicz E, Pogorzelski A, Klimek B, Voelkel K, Witt M. CFTR mutations spectrum and the efficiency of molecular diagnostics in Polish cystic fibrosis patients. PLoS One. 2014 Feb 26;9(2):e89094. doi: 10.1371/journal.pone.0089094. eCollection 2014.

Zimowski J, Kulczycki J, Lojkowska W, Szpak G, Krysa W, Szirkowiec W, Limon-Sztencel A, Zaremba J. Hereditary form of prion disease in Poland.Neurol Neurochir Pol. 2012 Nov-Dec;46(6):509-18. doi: 10.5114/ninp.2012.32353.

Zmyslowska A, Borowiec M, Antosik K, Ploski R, Ciechanowska M, Iwaniszewska B, Jakubiuk-Tomaszuk A, Janczyk W, Krawczynski M, Salmonowicz B, Stelmach M, Mlynarski W. Genetic evaluation of patients with Alström syndrome in the Polish population. Clin Genet. 2016 Apr;89(4):448-453. doi: 10.1111/cge.12656. Epub 2015 Sep 24.

Zupan A, Fakin A, Battelino S, Jarc-Vidmar M, Hawlina M, Bonnet C, Petit C, Glavač D. Clinical and Haplotypic Variability of Slovenian USH2A Patients Homozygous for the c. 11864G>A Nonsense Mutation. Genes (Basel). 2019 Dec 5;10(12):1015. doi: 10.3390/genes10121015.

Żurowska AM, Bielska O, Daca-Roszak P, Jankowski M, Szczepańska M, Roszkowska-Bjanid D, Kuźma-Mroczkowska E, Pańczyk-Tomaszewska M, Moczulska A, Drożdż D, Hadjipanagi D, Deltas C, Ostalska-Nowicka D, Rabiega A, Taraszkiewicz J, Taranta-Janusz K, Wieczorkiewicz-Plaza A, Jobs K, Mews J, Musiał K, Jakubowska A, Nosek H, Jander AE, Koutsofti C, Stanisławska-Sachadyn A, Kuleszo D, Ziętkiewicz E, Lipska-Ziętkiewicz BS. Mild X-linked Alport syndrome due to the COL4A5 G624D variant originating in the Middle Ages is predominant in Central/East Europe and causes kidney failure in midlife. Kidney Int. 2021 Jun;99(6):1451-1458. doi: 10.1016/j.kint.2020.10.040. Epub 2020 Dec 10.

Supplementary Table S2 – Spectrum and share of recurrent pathogenic alleles characteristic for Slavs and four well-known founder populations (Jewish, Icelandic, Finnish, French Canadians)

**Disease category**	**Founder alleles**				
	**Slavic populations**	**Jewish populations**	**Icelandic population**	**Finnish population**	**French Canadians**
**Inborn errors of metabolism**	**Phenylketonuria (PKU)** *PAH* c.1222C>T (p.Arg408Trp) **Wilson disease** *ATP7B* c.3207C>A (p.His1069Gln) **Alkaptonuria** *HGD* c.481G>A (p.Gly161Arg); also reported as c.648G>A **Mucopolysaccharidosis VI (Maroteaux-Lamy)** *ARSB* c.454C>T (p.Arg152Trp) **Glycogen storage disease type 1b** *SLC37A4* c.1042_1043delCT (p.Leu348Valfs*53) **Smith-Lemli-Opitz syndrome** *DHCR7* c.452G>A (p.Trp151*); c.976G>T (p.Val326Leu) **Alpha-mannosidosis** *MAN2B1* c.2248C>T (p.Arg750Trp, also reported as p.Arg749Trp)	**Gaucher disease** *GBA1* c.84dup (p.Leu29fs) c.1226A>G (p.Asn409Ser) [Diaz et al., 2000] **Tay-Sachs disease** *HEXA* c.1274_1277dup (p.Tyr427fs) [Frisch et al., 2004] **Mucolipidosis type IV** *MCOLN1* c.406-2A>G exon 1-7 deletion [Bargal et al., 2000] **Glycogen storage disease Ia** *G6PC1* c.247C>T (p.Arg83Cys) [Ekstein et al., 2004] **Glycogen storage disease VII** *PFKM* c.237+1G>A (also reported as NM_001166686.2:c.450+1G>A); c.2003del (p.Pro668fs) [Sherman et al., 1994] **Maple syrup urine disease type 1b** *BCKDHB* c.548G>C (p.Arg183Pro) [Edelmann et al., 2001] **Tyrosinemia type 1** *FAH* c.782C>T (p.Pro261Leu)	**Phenylketonuria** *PAH* c.1129del (p.Tyr377fs) [Guldberg et al., 1997] **Wilson disease** *ATP7B* c.2009_2015del (p.Ile669_Tyr670insTer, also reported as 1950del7, c.2007del7) [Palsson et al., 2001] **cblB-type methylmalonic acidemia** *MMAB* c.571C>T (p.Arg191Trp) [Agnarsdóttir et al., 2022] **GM1-gangliosidosis** *GLB1* c.557 A > C (p.Glu186Ala) [Arnadottir et al., 2022]	**Aspartylglucosaminuria** *AGA* c.488G>C [Mononen et al., 1991] **Lysinuric protein intolerance** *SLC7A7* c.895-2A>T p.Thr299Ilefs*10 [Torrents et al., 1999] **Sialuria, Finnish type (Salla disease)** *SLCL17A5* c.115C>T (p.Arg39Cys) [Aula et al., 2000] **Hereditary tyrosinemia** *FAH* c.786G > A, (p.Trp262X) [Äärelä et al., 2020]	**Tay-Sachs disease** *HEXA* c.805+1G>A; 7.5-kb deletion [Scriver et al., 2001] **Hereditary tyrosinemia** *FAH* c.1062+5G>A [Scriver et al., 2001] **Mucolipidosis II** *GNPTAB* c.3503_3504del (p.Leu1168fs) [Plante et al., 2008] **Hyperornithinaemia-hyperammonaemia-homocitrullinuria (HHH) syndrome** *SLC25A15* c.553TTC[3] (p.Phe188del) [Debray et al., 2009]

		[Wallace and Bean et al., 2018]<br>**Multiple sulphatase deficiency**<br>*SUMF1* c.463T>C (p.Ser155Pro) [Wallace and Bean et al., 2018]<br>**Wilson disease**<br>*ATP7B* c.3207C>A (p.H1069Q); c.3191A>C (p.E1064A) [Wallace and Bean et al., 2018]<br>**Galactosemia**<br>*GALT* del5.5kb [Daas et al., 2023]<br>**Niemann-Pick disease, type A**<br>*SMPD1* c.911T>C (p.Leu304Pro, also reported as L302P); c.996delC (p.Phe333Serfs, also reported as fsP330); c.1493G>T (p.R498L, also reported as R496L) [Schuchman and Miranda, 1997]			
**Hereditary cancer syndromes**	**Hereditary breast and ovarian cancer (HBOC)**<br>*BRCA1* c.5266dupC (p.Gln1756ProfsX74), according to BIC nomenclature: 5382insC (p.Gln1777fs)<br>**Hereditary breast cancer**<br>*PALB2* c.168_171delTTGT (p.Gln60fs, also reported as c.172_175delTTGT); c.509_510delGA (p.Arg170Ilefs)<br>**Low penetrance breast**	**Hereditary breast and ovarian cancer (HBOC)**<br>*BRCA1* c.68_69del (p.Glu23fs, also reported as 185delAG); c.5266dup (p.Gln1756fs, also reported as 5382insC)<br>*BRCA2* c.5946del (p.Ser1982fs, also reported as 6174delT) [Yanus et al., 2022]<br>**Low penetrance breast cancer predisposition**<br>*CHEK2* c.1283C>T	**Hereditary breast and ovarian cancer (HBOC)**<br>*BRCA2* c.771_775del (p.Asn257fs, also reported as 999del5) [Yanus et al., 2022]<br>**Low penetrance breast cancer predisposition**<br>*BARD1* c.1670G>C (p.Cys557Ser) [Stacey et al., 2006]<br>**Ovarian cancer predisposition**<br>*BRIP1* c.2038_2039dupTT	**Lynch syndrome**<br>*MLH1* Ex 16 deletion; c.454–1G>A [Salovaara et al., 2000]<br>**Hereditary breast cancer**<br>*PALB2* c.1592delT (p.Leu531fs) [Erkko et al., 2007]<br>**Ovarian cancer predisposition**<br>*RAD51C* c.837 + 1G > A c.93delG (p.Phe32fs); partial duplication	**Hereditary breast and ovarian cancer (HBOC)**<br>*BRCA1* c.4327C>T (p.Arg1443*, also reported as 4446C>T);<br>*BRCA2* c.8537_8538del (p.Glu2846fs, also reported as 8761delAG) [Cavallone et al., 2010]<br>**Low penetrance breast cancer predisposition**<br>*PALB2* c.2323C>T (p.Gln775Ter)<br>**Ovarian cancer**

	**cancer predisposition**<br>*CHEK2* c.(908+1_909-1)_(1095+1_1096-1)del; also reported as 5395 bp deletion, 5567 deletion, deletion of exons 9 and 10; L303Fdel304_391; c.444+1G>A; also reported as IVS3+1G>A; IVS2+1G>A<br>*ATRIP* c.1152_1155del (p.Thr384_Gly385insTer)<br>**MUTYH-associated polyposis**<br>*MUTYH* c.734G>A (p.Arg245His)	(p.Ser428Phe) [Wallace and Bean, 2018]<br>**Lynch syndrome (HNPCC)**<br>*MSH2* c.1906G>C (p.Ala636Pro)<br>*MSH6* c.3959_3962del (p.Ala1320fs); c.3984_3987dup (p.Leu1330fs)<br>[Ponti et al., 2015]<br>**Hereditary mixed polyposis syndrome**<br>**Hereditary mixed polyposis syndrome**<br>*GREM1* 40-kb duplication in promoter [Wallace and Bean et al., 2018]	(p.Leu680Phefs) [Rafnar et al., 2011]<br>**Lynch syndrome**<br>*MSH6* c.1754T>C (p.Leu585Pro)<br>*PMS2* c.736_741del6ins11 (p.Pro246Cysfs*3); c.2T>A (p.Met1?) [Haraldsdottir et al., 2017]<br>**Melanoma-pancreatic cancer syndrome e**<br>*CDKN2A*<br>c.266G>A (p.Gly89Asp) [Goldstein et al., 2008]	[Pelttari et al., 2018]<br>*RAD51D* c.576+1G>A [Pelttari et al., 2012]<br>**Pituitary adenoma predisposition**<br>*AIP*<br>c.40C>T (p.Gln14Ter) [Vierimaa et al., 2006]<br>**Bone marrow failure/erythroid lineage-restricted acute myeloid leukemia**<br>ERCC6L2 c.1424delT (p.Ile475ThrfsTer36) [Douglas et al., 2019]	**predisposition**<br>*RAD51D* c.620C>T (p.Ser207Leu) [Fierheller et al., 2021]<br>**Lynch syndrome (HNPCC)**<br>*MSH2* c.942+3A>T<br>*MLH1*<br>c.2195_2198dup(p.His733 fs); exon 12 deletion [Ponti et al., 2015]<br>*MSH6*<br>c.10C>T (p.Gln4*)<br>[Castellsagué et al., 2015]
**Neurological and neuromuscular diseases**	**Spinocerebellar ataxia 1**<br>*ATXN1* c.589_591CAGins (p.Gln208_His209ins, also reported as (CAG)n repeat expansion)<br>**Neurodegeneration with brain iron accumulation 4**<br>*C19Orf12* c.204_214del11 (p.Gly69Argfs) (also reported as c.171_181del, p.Gly58fs)<br>**Stress-induced childhood-onset neurodegeneration with variable ataxia and seizures**<br>*ADPRS* (also reported as *ADPRHL2*) c.1004T>G (p.Val335Gly)<br>**Charcot–Marie–Tooth type 4A disease**<br>*GDAP1* c.715C>T (p.Leu239Phe)<br>**Hereditary axonal**	**Canavan disease**<br>*ASPA*<br>c.854A>C (p.Glu285Ala) [Kaul et al., 1993]<br>**Familial dysautonomia**<br>*ELP1* (IKBKAP) c.2204+6T>C [Slaugenhaupt et al., 2001]<br>**Early-Onset Isolated Dystonia**<br>*TOR1A* c.904_906delGAG (p.Glu303del) [Bressman et al., 2000]<br>**Spastic tetraplegia, thin corpus callosum, and progressive microcephaly**<br>*SLC1A4* c.766G>A (p.Glu256Lys) [Heimer et al., 2015]<br>**Amyotrophic lateral sclerosis** (more frequent in North African Jews)	**Mild, early-onset, sensory-negative, axonal polyneuropathy**<br>*PRPH* c.996+1G>A [Bjornsdottir et al., 2019]<br>**Early infantile epileptic encephalopathy, type 25**<br>*SLC13A5* c.655G>A (p.Gly219Arg) [Arnadottir et al., 2022]<br>**Intellectual disability, seizures, microcephaly, and abnormal muscle tone**<br>*CPSF3* c.1403G>A (p.Gly468Glu) [Arnadottir et al., 2022]<br>**Brown-Vialetto-Van Laere syndrome, type 2**<br>SLC52A2 c.1016 T>C (p.Leu339Pro) [Gudbjartsson et al., 2015]	**Unverricht-Lundborg disease (Progressive Myoclonic Epilepsy Type 1A)**<br>CSTB g.4900_4935CCCCGCC CCGCG (c.-210_-199(30_125)) repeat expansion [Moulard et al., 2002]<br>**Ceroid lipofuscinosis, neuronal, 1**<br>PPT1 c.364A>T (p.Arg122Trp) [Vesa et al., 1995]<br>**Cohen syndrome**<br>*VPS13B*<br>c.3348_3349delCT (p.Cys1117Phefs*8) [Kolehmainen et al., 2003]<br>**Lethal congenital**	**Spastic ataxia of Charlevoix-Saguenay**<br>*SACS* c.8844del (p.Ile2949fs) [Scriver et al., 2001<br>**Agenesis of the corpus callosum and peripheral neuropathy (Andermann syndrome)**<br>*SLC12A6* c.2436delG (p.Thr813Profs); c.1584_1585delCTinsG (Phe529fsX531) [Dupre et al., 2003]<br>**Oculopharyngeal muscular dystrophy**<br>*PABPN1* (GCG)6 repeat expansion [Scriver et al., 2001]<br>**Myotonic dystrophy type 1 (dystrophia myotonica or Steinert disease)**

	**neuropathy accompanied by neuromyotonia**<br>*HINT1* c.110G>C (p.Arg37Pro)<br>**Hereditary spastic paraplegia (SPG47)**<br>*AP4B1* c.1160_1161del (p.Thr387Argfs)	*OPTN* c.381_382insAG (p.Asp128ArgfsTer22, also reported as 691_692insAG [Goldstein et al., 2016; Zlotogora et al., 2018]<br>**Arthrogryposis, mental retardation, and seizures (AMRS)**<br>*SLC35A3* c.886A>G (p.Ser296Gly) [Wallace and Bean, 2018]<br>**Pontocerebellar hypoplasia type 1A**<br>*VRK1* c.1072C>T (p.Arg358Ter) [Wallace and Bean, 2018]<br>**Congenital disorder of glycosylation Ia**<br>*PMM2* c.422G>A (p.Arg141His) [Wallace and Bean, 2018]<br>**3-phosphoglycerate dehydrogenase deficiency**<br>*PHGDH* c.1468G>A (p.Val490Met) [Wallace and Bean, 2018]<br>**Walker-Warburg syndrome**<br>*FKTN* c.1167dupA (p.Phe390fs) [Wallace and Bean et al., 2018]<br>**GBE1 Adult Polyglucosan Body Disease**<br>*GBE1* c.986A>C (p.Tyr329Ser) c.2053-5289_2053-5297delinsTGTTTTTTACATGACAGGT [Akman et al., 2015]		**contracture syndrome 1 (fetal motoneuron disease, Herva disease)**<br>*GLE1* c.433-10A>G [Nousiainen et al., 2008]<br>**Glycine encephalopathy**<br>*GLDC* c.1691G>T (p.Ser564Ile) [Kure et al., 1992]<br>**Muscular dystrophy-dystroglycanopathy (congenital with brain and eye anomalies), type A, 3**<br>*POMGNT1* c.1539+1G-A [Diesen et al., 2004]<br>**Progressive encephalopathy with edema, hypsarrhythmia, and optic atrophy (PEHO) syndrome**<br>*ZNHIT3* c.92C>T (p.Ser31Leu) [Anttonen et al., 2017]<br>**Frontotemporal predominant pachygyria**<br>*CRADD* c.509G>A (p.Arg170His) [Polla et al., 2019]	*DMPK* (CTG)n repeat expansion [Yotova et al., 2005]<br>**Zellweger syndrome**<br>*PEX6* c.802_815del, (p.[Val207_Gln294del, Val76_Gln294del]) [Levesque et al., 2012]<br>**Chorea-acanthocytosis**<br>*VPS13A*<br>exon 70-73 deletion [Dobson-Stone et al., 2005]

		**Cerebellar ataxia and developmental delay/epileptic encephalopathy** *THG1L* c.153C>G (p.Cys51Trp); c.164T>C (p.Val55Ala) [Rabin et al., 2018]<br>**Choreoacanthosis** *VPS13A* c.6058delC (p.Pro2020LeufsTer9) [Lossos et al., 2005]<br>**Childhood-onset dystonia and optic atrophy** *MECR* c.830+2_830+3insT (p.Glu303del); c.695G>A (p.Gly232Glu) [Heimer et al., 2016]<br>**Hereditary sensory autonomic neuropathy with intellectual disability** *TECPR2* c.1319delT (p.Leu440ArgfsTer19) [Neuser et al., 2021]<br>**Infantile onset leukoencephalopathy** *VPS11* c. 2536T>G (p.Cys846Gly) [Zhang et al., 2016]<br>***HIKESHI*-Related hypomyelinating leukodystrophy** *HIKESHI/C11ORF73* c.160G>C (p.Val54Leu**)** [Helman et al., 2021]			
**Hereditary endocrinopathies**	**Combined pituitary hormone deficiency-2** *PROP1* c.301_302delGA (p.Leu102Cysfs); c.150del (p.Arg53Aspfs)	**Congenital hyperinsulinism of infancy** *ABCC8* c.3989-9G>A (also reported as 3992-9G>A,		**Autoimmune polyendocrinopathy–candidiasis–ectodermal dystrophy (APECED)** *AIRE* c.769C>T	**Pseudo vitamin D deficiency rickets** *CYP27B1* c.262del (p.Val88fs) [Scriver et al., 2001]

	**Hypogonadotropic hypogonadism 7 without anosmia (normosmic congenital hypogonadotropic hypogonadism)**<br>*GNRHR* c.416G>A (p.Arg139His)<br>**Allgrove syndrome (triple A syndrome)**<br>*AAAS* c.787T>C (p.Ser263Pro); c.43C>A (p.Gln15Lys, also reported as p.Gly14Valfs*45 reflecting the impact of this substitution on splicing)<br>**Autoimmune polyendocrinopathy–candidiasis–ectodermal dystrophy (APECED)**<br>*AIRE* c.769C>T (p.Arg257*) (common between Slavs and Finns, probably of Finnish origin)	exon alpha del); c.4160_4162del (p.Phe1387del) [Glaser et al., 2011]<br>**Non-classic primary adrenal hyperplasia**<br>CYP21A2 c.844G>C (p.Val282Leu) [Hannah-Shmouni et al., 2017]		(p.Arg257*) (common between Slavs and Finns, probably of Finnish origin)	**Autoimmune polyendocrinopathy–candidiasis–ectodermal dystrophy (APECED)**<br>*AIRE* c.1616C>T (p.Pro539Leu) [Cruz Marino et al., 2022b]
**Primary immunodeficiencies and bone marrow failure syndromes**	**Severe combined immunedeficiency (SCID)**<br>*RAG1* c.256_257delAA (p.Lys86Valfs) [Sharapova et al., 2020]<br>**C2 complement deficiency**<br>*C2* c.841_849 + 19del (p.Val281Profs) [Blazina et al., 2018; Yanus et al., 2019]<br>**Complement component 8B deficiency**<br>*C8B* c.1282C>T (p.Arg428*)<br>**Ataxia-telangiectasia**<br>*ATM* c.5932G>T (p.Glu1978Ter)<br>**Bloom syndrome**<br>*BLM* c.1642C>T (p.Gln548*)	**Severe combined immunedeficiency (SCID)**<br>*DCLRE1C* c.1299_1306dupAGGATGCT [Rechavi et al., 2017]; *IL7Rα* c.120C>G (p. Phe40Leu) [Rechavi et al., 2017]<br>**C2 complement deficiency**<br>*C2* c.841_849 + 19del (p.Val281Profs) [gnomAD database]<br>**Bloom syndrome** *BLM* c.2207_2212delinsTAGATTC (p.Tyr736fs) [Arora et al., 2014]	**Chronic granulomatous disease**<br>*CYBC1* c.6C>G (p.Tyr2Ter) [Arnadottir et al., 2018]	**Hyper-IgM syndrome type 2**<br>*AICDA* c.416T>C (p.Met139Thr) [Trotta et al., 2016]<br>**ADA2 deficiency**<br>*CECR1* c.506G>A (p.Arg169Gln) [Trotta et al., 2018]	**CARD9 deficiency (invasive fungal disease susceptibility)**<br>*CARD9* c.439T>C (p.Y91H); unidentified variant in cis with c.-529T>C SNP [Gavino et al., 2016]

	**Nijmegen breakage syndrome**<br>*NBN* c.657_661del (p.Lys219fs) (also reported as c.657del5)	**Fanconi Anemia (FA) complementation group C**<br>*FANCC* c.456 + 4A > T (IVS4+4A > T) [Verlander et al., 1995]<br>**Aicardi-Goutieres syndrome**<br>*SAMHD1* exon 1 deletion [Straussberg et al., 2014]<br>**Mediterranean periodic fever**<br>*MEFV* c.2177T>C (p.Val726Ala) [Adato et al., 2022; Papadopoulos et al., 2008]			
**Kidney diseases**	**Hypomagnesemia 3, renal (Familial hypomagnesaemia with hypercalciuria and nephro-calcinosis (FHHNC))**<br>*CLDN16*: c.453G>T (p.Leu151Phe)<br>**Idiopathic infantile hypercalcemia**<br>*CYP24A1* c.1186C>T (p.Arg396Trp)<br>**Nephrotic syndrome, type 12**<br>*NUP93* c.1772G>T (p.Gly591Val)<br>**Alport syndrome-1**<br>*COL4A5* c.1871G>A (p.Gly624Asp)<br>**Nephrotic syndrome type 2**<br>*NPHS2* c.868G>A (p.Val290Met)	**Primary hyperoxaluria type III**<br>*HOGA1* c.860G>T (p.Gly287Val); c.944_946delAGG (p.Glu315del) [Abid, 2021]<br>**Autosomal recessive polycystic kidney disease**<br>*PKHD1* c.3761_3762delCCinsG (p.Ala1254GlyfsX49) [Quint et al., 2015]<br>**Alport syndrome** *COL4A3* c.40_63del (p.Leu14_Leu21del) [Webb et al., 2014]<br>**Cystinuria**<br>*SLC3A1* c.808C>T (p.Arg270*) [Pras et al., 1995]<br>**Steroid resistant nephrotic syndrome and prenatal onset ventriculomegaly with kidney disease**	**Adenine phosphoribosyltransferase deficiency**<br>*APRT* c.194A>T (p.Asp65Val) [Runolfsdottir et al., 2021]	**Nephrotic syndrome, type 1 (Finnish congenital nephrosis)**<br>*NPHS1* c.121_122del (p.Leu41fs); c.3325C>T (p.Arg1109Ter) [Kestilä et al., 1998]	**Cystinosis**<br>*CTNS* c. 414G>A (p.Trp138*) [McGowan-Jordan et al., 1999]

		CRB2 c.2400C>G (p.Asn800Lys) [Slavotinek et al., 2015; Jaron et al., 2016]			
**Hearing loss and eye diseases**	**Leber hereditary optic neuropathy (Leigh syndrome phenotype may occur)** *DNAJC30* c.152A>G (p.Tyr51Cys) **Usher syndrome type IB** *MYO7A* c.52C>T (p.Gln18*) **Usher syndrome type IIA** *USH2A* c.11864G>A (p.Trp3955*)	**Retinitis pigmentosa** (various types) *MAK* c.1297_1298ins353 (also reported as c.1297ins(Alu)) *DHDDS* c.124A>G (p.Lys42Glu) *FAM161A* c.1355_1356delCA *HGSNAT* c.370A>T (p.Arg124Trp) [Zlotogora et al., 2018] **X-linked incomplete congenital stationary night blindness** *CACNA1F* c.2225T>G (p.Phe742Cys) [Kimchi et al., 2019] **Congenital stationary night blindness** *TRPM1* exons 2-7 deletion [AlTalbishi et al., 2019] **AR hearing impairment (DFNB1)** *GJB2* c.167delT (p.Leu56fs) **AR hearing impairment (DFNB4)** *SLC26A4* c.349C>T p.(Leu117Phe) [Brownstein et al., 2020] **AR hearing impairment** (various types) *LOXHD1* c.4714C>T (p.Arg1572Ter) [Wallace and Bean et al., 2018]	**Sveinsson's chorioretinal atrophy** *TEAD1* c.1261T>C (p.Tyr421His) [Fossdal et al., 2004] **Retinitis pigmentosa** (various types) *RLBP1* c.832C>T (p.Gln278*); c.677T>A (p.Met226Lys) *PRPF31* c.1073 + 5G > A [Thorsteinsson et al., 2021] **X-linked retinoschisis** *RS1* c.441G>A (p.Trp147X) [Thorsteinsson et al., 2021]	**Retinal dystrophy** *CERKL* c.375C>G (p. Cys125Trp); c.193G>T (p. Glu65Ter) **Retinitis pigmentosa** *EYS* c.1155T>A (p.Cys385Ter) [Avela et al., 2018] **X-linked juvenile retinoschisis** *RS1* c.214G>A (p.Glu72Lys); c.221G>T (p.Gly74Val); c.325G>C (p.Gly109Arg) [Huopaniemi et al., 1999] **Cornea plana 2, autosomal recessive** *KERA* c.740A>G (p.Asn247Ser) [Pellegata et al., 2000] **Gyrate atrophy of choroid and retina with or without ornithinemia** *OAT* c.1205T>C (p.Leu402Pro) [Mitchell et al., 1989] **Usher syndrome, type 3A** *CLRN1* c.528T>G (p.Tyr176Ter, also reported as 300C-T (TYR100TER)) [Joensuu et al., 2001]	**Autosomal-recessive hearing loss** *PDZD7* c.2672AGA[1] (p.Lys892del) [Cruz Marino et al., 2022a] **Usher syndrome type 1** *USH1C* c.216G>A (p.Val72=, also reported as 'Acadian allele') **Usher syndrome type 2** *USH2A* c.4338_4339del (p.Cys1447fs) [Ebermann et al., 2009]

		SLC26A4 c.349C>T p.(Leu117Phe) [Brownstein et al., 2020]<br>**Usher syndrome, type 1F**<br>*PCDH15* c.733C>T (p.Arg245Ter) [Ben-Yosef et al., 2003]<br>**Usher syndrome, type 3A**<br>*CLRN1* c.144T>G (p.Asn48Lys) [Ness et al., 2003]<br>**Enhanced S-cone syndrome**<br>*NR2E3* c.932G>A (p.Arg311Gln) [Bandah et al., 2009]<br>**Leber congenital amaurosis**<br>*LCA5* c.835C>T (p.Gln279Ter) [Jacobson et al., 2009]			
**Skin diseases**	**Autosomal recessive congenital ichthyosis-3 (lamellar ichthyosis; self-healing collodion baby)**<br>*ALOXE3* c.1096C>T (p.Arg366*, also reported as c.700C>T (p. Arg234*))<br>**Acral peeling skin syndrome**<br>*TGM5* c.337G>T (p.Gly113Cys)<br>**Dystrophic epidermolysis bullosa**<br>*COL7A1* c.425A>G (p.Lys142Arg)	**Pachyonychia congenita**<br>*KRT16* c.380G>A (p.Arg127His) [Pavlovsky et al., 2021]<br>**Dowling-Degos disease**<br>*PSENEN* c.168T>G (p.Y56X) [Pavlovsky et al., 2018]<br>**Peeling skin syndrome**<br>*CDSN* c.164_167dup GCCT (p.Thr57ProfsTer6) [Zlotogora et al., 2018]<br>**Xeroderma pigmentosum**<br>*XPC* c.566_567delAT (p.Tyr189SerfsTer10) [Zlotogora et al., 2018]<br>**Ehlers–Danlos syndrome, dermatosparaxis type**<br>*ADAMTS2* c.673C>T			

		(p.Gln225Ter) [Wallace and Bean et al., 2018]			
**Heart, lungs, vascular and blood diseases**	**Ciliary dyskinesia, primary, 38**<br>*CFAP300* (formerly *C11orf70*) c.198_200delTTTinsCC (p.Phe67Profs)<br>**Primary ciliary dyskinesia, type 1**<br>*DNAI1* c.1612G>A (p.Ala538Thr)<br>**Hypertrophic cardiomyopathy**<br>*MYBPC3* c.3697C>T (p.Gln1233*)	**Coagulation factor XI deficiency**<br>*F11* c.403G>T (p.Glu135Ter, also reported as p.Glu117X or type II); c.901T>C (p.Phe301Leu, also reported as p.Phe283Leu or type III) [Peretz et al., 1997]<br>**Pernicious anemia, juvenile**<br>*CUBN* c.2614_2615delGA (p.Asp872Leufs) [Zlotogora et al., 2018]<br>**Hermansky-Pudlak syndrome**<br>*HPS3* c.1163+1G>A [Wallace and Bean et al., 2018]<br>**Congenital amegakaryocytic thrombocytopenia**<br>*MPL* c.79+2T>A [Jalas et al., 2011]<br>**Familial hypercholesterinemia**<br>*LDLR* c.651TGG[1] (p.Gly219del, also reported as G197del, FH Lithuania, FH Piscataway) [Durst et al., 2001]<br>**Congenital cardiac valve malformations**<br>*PLD1* c.2002A>T (p.Ile668Phe) [Lahrouchi et al., 2021]<br>**Arrhythmogenic or dilated cardiomyopathy**	**Hypertrophic cardiomyopathy**<br>*MYBPC3* c.927-2A>G [Adalsteinsdottir et al., 2017]<br>**Coarctation of the aorta; sick sinus syndrome**<br>*MYH6* c.2161C>T (p.Arg721Trp) [Bjornsson et al., 2018]<br>**Early-onset atrial fibrillation**<br>*MYL4* c.234delC (p.Cys78Trpfs*29) [Gudbjartsson et al., 2015]<br>**Familial hypercholesterolemia**<br>*LDLR* c.694+2T>C [Björnsson et al., 2021]	**Megaloblastic anemia 1**<br>*CUBN* c.3890C>T (p.Pro1297Leu) [Tanner et al., 2012]	**Familial hypercholesterolemia**<br>*LDLR* >15 kb deletion (promoter and exon 1, also reported as Δ>15kb); c.259T>G (p.Trp87Gly, also reported as W66G) [Scriver et al., 2001]<br>**Familial chylomicronemia**<br>*LPL* c.701C>T (p.Pro234Leu) [Scriver et al., 2001]<br>**Beta-Thalassemia**<br>*HBB* c.93-21G>A (also reported as IVS1:110 G>A); c.118C>T (p.Gln40Ter) [Scriver et al., 2001]

		FLNC c.3791-1G>C [Liebman et al., 2021]<br>**Primary ciliary dyskinesia** (various types)<br>*CCDC65* c.877_878del (p.Ile293fs)<br>*CFAP298 (C21orf59)* c.735C>G (p.Tyr245Ter)<br>*DNAH5* c.7502G>C (p.Arg2501Pro)<br>*DNAI2* c.1304G>A (p.Trp435Ter)<br>DNAI1 c.1490G>A (p.Gly497Asp)<br>*DNAH11* c.11929G>T (p.Glu3984Ter)<br>*CCDC114* c.939delT (p.His313Glnfs)<br>*CCNO* c.638T>C (p.Leu213Pro)<br>[Zlotogora et al., 2018]<br>**Cerebral cavernous malformations**<br>*CCM2* c.30+5_30+6delGCinsTT [Wallace and Bean et al., 2018]<br>**Cystic fibrosis**<br>*CFTR* c.3846G>A (p.Trp1282Ter) [Behar et al., 2017]			
**Gastrointestinal disorders**		**Abetalipoproteinemia**<br>*MTTP* c.2212delT (p.Ser738fs); c.2593G>T (p.Gly865Ter) [Wallace and Bean, 2018]<br>**Protein losing enteropathy (PLE)**<br>*DGAT1* c.751+2T>C [Stephen et al., 2016]		**Congenital chloride diarrhea**<br>*SLC26A3* c.951_953del (p.Val318del) [Wedenoja et al., 2011]<br>**Lactase deficiency, congenital**<br>*LCT* c.4170T>A (p.Tyr1390Ter)	

				[Kuokkanen et al., 2006]	
**Skeletal, connective tissue and muscular disorders**	**Limb-girdle muscular dystrophy type 2A**<br>*CAPN3* c.550delA (p.Thr184Argfs)	**Osteopetrosis**<br>*TCIRG1* c.117+4A>T [Anderson et al., 2015]<br>**Nemaline myopathy**<br>*NEB* exon 55 deletion [Wallace and Bean, 2018]<br>**Myasthenia gravis, congenital**<br>*CHRNE* c.1353dupG (p.Asn452GlufsTer4) [Wallace and Bean, 2018]	**RYR1-related disorder (malignant hyperthermia)**<br>*RYR1* c.2870+1G>A [Fridriksdottir et al., 2021]	**Atelosteogeneis Type II and Diastrophic Dysplasia**<br>*SLC26A2* c.-26+2T>C [Hästbacka et al., 1999]<br>**RAPADILINO syndro me**<br>*RECQL4* c.1390+2delT [Siitonen et al., 2009]<br>**Tibial muscular dystrophy (TMD)**<br>*TTN* c.107780_107790delins TGAAAGAAAAA (p.Glu35927_Trp35930d elins ValLysGluLys) [Hackman et 2002]	**Pseudoxanthoma elasticum**<br>*ABCC6* c.3412C>T (p.Arg1138Trp) [LaRusso et al., 2010]
**Infertility/Recurrent stillbirths**		**Early miscarriages / Smith-Lemli-Opitz (SLOS) syndrome**<br>*DHCR7* c.964-1G>C [Daum et al., 2020]	**Early embryonic death**<br>*GLE1* c.1706G>A (p.Arg569His) [Arnadottir et al., 2022]	**Ovarian dysgenesis (FSH-resistant ovaries)**<br>*FSHR* c.566C>T (p.Ala189Val) [Doherty et al., 2002]	
**Mitochondrial diseases**	**Mitochondrial DNA depletion syndrome 3 (hepatocerebral type)**<br>*DGUOK* c.3G>A (p.Met1?)<br>**Mitochondrial complex IV deficiency nuclear type 2/ Leigh syndrome**<br>*SCO2* (also known as *NCAPH2*) c.418G>A (p.Glu140Lys)<br>**Mitochondrial complex IV deficiency, nuclear type 1/ Leigh syndrome**<br>*SURF1* c.841_842delCT (p.Ser282Cysfs); also reported as: 845-846delCT	**Leigh syndrome**<br>*NDUFAF5* c.749G>T (p.Gly250Val) [Zlotogora et al., 2018]<br>**Acyl CoA dehydrogenase deficiency, short / branched chains**<br>*ACADS* c.319C>T (p.Arg107Cys) [Zlotogora et al., 2018]<br>**Carnitine palmitoyl transferase deficiency**<br>*CPT2* c.338C>T (p.Ser113Leu); c.1238_1239delAG (p.Lys414ThrfsTer7)		**mtDNA depletion syndrome 7 (hepatocerebral type) (IOSCA)**<br>*C10ORF2* c.1523A>G (p.Tyr508Cys) [Nikali et al., 2005]<br>**GRACILE syndrome**<br>*BCS1L* c.232A>G (p.Ser78Gly) [Visapää et al., 2002]<br>**Combined oxidative phosphorylation deficiency 3 (probably embryonic lethality)**<br>*TSFM* c.856C>T	**Leigh syndrome, French Canadian type**<br>*LRPPRC* c.1061C>T (p.Ala354Val) [Debray et al., 2011]<br>**HSD10 mitochondrial disease (attenuated phenotype)**<br>*HSD17B10* c.364C>G (p.Leu122Val) [Waters et al., 2019]

		[Taggart et al., 1999]<br>**Leigh syndrome**<br>*ATP5MK* (also known as *USMG5)* c.87 + 1G>C [Barca et al., 2018]<br>**Mitochondrial encephalomyopathy**<br>COQ4 c.718C>T (p.Arg240Cys) [Chung et al., 2015]		(p.Gln286Ter) [Lim et al., 2014]	
**Other multisystemic disorders**	**Cranioectodermal dysplasia** also known as **Sensenbrenner syndrome**<br>*WDR35* c.1889T>G (p.Leu630Ter) (<br>also reported as c.1922T>G p.(Leu641Ter))	**Hoyeraal–Hreidarsson syndrome** (severe type of dyskeratosis congenita)<br>*RTEL1* c.3791G>A (p.R1264H) [Fedick et al., 2015]<br>**Dihydrolipoamide Dehydrogenase Deficiency**<br>*DLD* c.685G>T (p.Gly229Cys) [Zlotogora et al., 2018]<br>**Bardet–Biedl syndrome**<br>*BBS2* c.311A>C (p.Asp104Ala); c.1895G>C (p.Arg632Pro) [Wallace and Bean et al., 2018]<br>**Joubert syndrome**<br>*TMEM216* c.218G>T (p.Arg73Leu) [Wallace and Bean, 2018]<br>**Zellweger spectrum disorder**<br>*PEX2* c.355C>T (p.Arg119Ter) [Wallace and Bean, 2018]<br>**Short stature, developmental delay, and congenital heart defects**<br>*TKT* c.769_770ins18	**Hereditary cystatin C amyloid angiopathy**<br>*CST3* c.281T>A (p.Leu94Gln) [Palsdottir et al., 2006]<br>**Recurrent miscarriage or perinatally lethal multisystemic disorder**<br>*GNE* c.1132 G>T (p.Asp378Tyr) [Arnadottir et al., 2022]	**Cartilage-hair hypoplasia** (dwarfism, anemia, defective antiviral immunity, hair abnormalities)<br>*RMRP* n.263G>T, also reported as A70G [Ridanpää et al., 2002]<br>**Mulibrey nanism**<br>*TRIM37* c.493-2A>G [Avela et al., 2000]<br>**Meckel syndrome (campomelic variant)**<br>*MKS1* c.1408-34_1408-6del [Auber et al., 2007]<br>**Hydrolethalus syndrome 1**<br>*HYLS1* c.632A>G p.Asp211Gly [Mee et al., 2005]<br>**Polycystic lipomembranous osteodysplasia with sclerosing leukoencephalopathy** (adult-onset psychosis, dementia and bone cysts)<br>*TYROBP* exon 1-4 deletion c.-2900_277-1238del [Paloneva et al.,	**Naxos disease**<br>*JUP* c.902A>G (p.Glu301Gly) [Marino et al., 2017]<br>**Joubert syndrome**<br>*C5ORF42* c.4006C>T (p.Arg1336Trp); c.4690G>A (p.Ala1564Thr), c.7400+1G>A [Srour et al., 2012]<br>**Congenital multiple intestinal atresia with combined immune deficiency**<br>*TTC7A* c.1001+3_1001+6del (also reported as c.53344_53347delAAGT) [Samuels et al., 2013]

		(p.Trp257delins7) [Boyle et al., 2016]		2000]<br>**Amyloidosis, Finnish type (FAF)**<br>*GSN* c.654G>A (p.Asp187Asn) [de la Chapelle et al., 1992]	
**Total: disorders (major founder alleles)**	**47 (52)**	**87 (119)**	**27 (29)**	**44 (50)**	**32 (40)**
**Population size**	**>270 million**	**10-14 million**<br>(at least 2.8 million in Israel)	**300 thousand**	**6-7 million**<br>(at least 4.7 million in Finland)	**6.8-10 million**<br>(at least 4.7 million in Canada, according to Census)
**Rough estimate of known founder alleles per 100000 population**	**0.019**	**0.86-1.19**	**9.7**	**0.71-0.83**	**0.4-0.6**
**Selected examples of "endemic" disorders, which rare outside founder population**	**Nijmegen breakage syndrome**	**Familial dysautonomia**<br><br>**Hereditary mixed polyposis syndrome**	**Sveinsson's chorioretinal atrophy**<br><br>**Hereditary cystatin C amyloid angiopathy**	**36 disorders, belonging to "Finnish disease heritage", e.g., aspartylglucosaminuria; hydrolethalus syndrome; sialic acid storage disease, Finnish type (Salla disease); mulibrey nanism; IOSCA syndrome; RAPADILINO syndrome [Norio, 2003]**	**Congenital multiple intestinal atresia with combined immune deficiency**<br><br>**Agenesis of the corpus callosum and peripheral neuropathy (Andermann syndrome)**

References to Supplementary Table S2

Äärelä L, Hiltunen P, Soini T, Vuorela N, Huhtala H, Nevalainen PI, Heikinheimo M, Kivelä L, Kurppa K. Type 1 tyrosinemia in Finland: a nationwide study. Orphanet J Rare Dis. 2020 Oct 12;15(1):281. doi: 10.1186/s13023-020-01547-w.

Abid A. Possible ethnic associations in primary hyperoxaluria type-III-associated HOGA1 sequence variants. Mol Biol Rep. 2021 Apr;48(4):3841-3844. doi: 10.1007/s11033-021-06380-3. Epub 2021 May 4. PMID: 33948853

Adalsteinsdottir B, Burke M, Maron BJ, Danielsen R, Lopez B, Diez J, Jarolim P, Seidman J, Seidman CE, Ho CY, Gunnarsson GT. Hypertrophic cardiomyopathy in myosin-binding protein C (MYBPC3) Icelandic founder mutation carriers. Open Heart. 2020 Apr 5;7(1):e001220. doi: 10.1136/openhrt-2019-001220. eCollection 2020.

Adato O, Brenner R, Levy A, Shinar Y, Shemer A, Dvir S, Ben-Zvi I, Livneh A, Unger R, Kivity S. Determining the origin of different variants associated with familial mediterranean fever by machine-learning. Sci Rep. 2022 Sep 8;12(1):15206. doi: 10.1038/s41598-022-19538-1.

Agnarsdóttir D, Sigurjónsdóttir VK, Emilsdóttir AR, Petersen E, Sigfússon G, Rögnvaldsson I, Franzson L, Vernon H, Bjornsson HT. Early cardiomyopathy without severe metabolic dysregulation in a patient with cblB-type methylmalonic acidemia. Mol Genet Genomic Med. 2022 Jul;10(7):e1971. doi: 10.1002/mgg3.1971. Epub 2022 Jun 16.

Akman HO, Kakhlon O, Coku J, Peverelli L, Rosenmann H, Rozenstein-Tsalkovich L, Turnbull J, Meiner V, Chama L, Lerer I, Shpitzen S, Leitersdorf E, Paradas C, Wallace M, Schiffmann R, DiMauro S, Lossos A, Minassian BA. Deep intronic GBE1 mutation in manifesting heterozygous patients with adult polyglucosan body disease. JAMA Neurol. 2015 Apr;72(4):441-5. doi: 10.1001/jamaneurol.2014.4496.

AlTalbishi A, Zelinger L, Zeitz C, Hendler K, Namburi P, Audo I, Sheffer R, Yahalom C, Khateb S, Banin E, Sharon D. TRPM1 Mutations are the Most Common Cause of Autosomal Recessive Congenital Stationary Night Blindness (CSNB) in the Palestinian and Israeli Populations. Sci Rep. 2019 Aug 19;9(1):12047. doi: 10.1038/s41598-019-46811-7.

Anderson SL, Jalas C, Fedick A, Reid KF, Carpenter TO, Chirnomas D, Treff NR, Ekstein J, Rubin BY. A founder mutation in the TCIRG1 gene causes osteopetrosis in the Ashkenazi Jewish population. Clin Genet. 2015 Jul;88(1):74-9. doi: 10.1111/cge.12448. Epub 2014 Jul 31.

Anttonen AK, Laari A, Kousi M, Yang YJ, Jääskeläinen T, Somer M, Siintola E, Jakkula E, Muona M, Tegelberg S, Lönnqvist T, Pihko H, Valanne L, Paetau A, Lun MP, Hästbacka J, Kopra O, Joensuu T, Katsanis N, Lehtinen MK, Palvimo JJ, Lehesjoki AE. ZNHIT3 is defective in PEHO syndrome, a severe encephalopathy with cerebellar granule neuron loss. Brain. 2017 May 1;140(5):1267-1279. doi: 10.1093/brain/awx040.

Arnadottir GA, Norddahl GL, Gudmundsdottir S, Agustsdottir AB, Sigurdsson S, Jensson BO, Bjarnadottir K, Theodors F, Benonisdottir S, Ivarsdottir EV, Oddsson A, Kristjansson RP, Sulem G, Alexandersson KF, Juliusdottir T, Gudmundsson KR, Saemundsdottir J, Jonasdottir A, Jonasdottir A, Sigurdsson A, Manzanillo P, Gudjonsson SA, Thorisson GA, Magnusson OT, Masson G, Orvar KB, Holm H, Bjornsson S, Arngrimsson R, Gudbjartsson DF, Thorsteinsdottir U, Jonsdottir I, Haraldsson A, Sulem P, Stefansson K. A homozygous loss-of-function mutation leading to CYBC1 deficiency causes chronic granulomatous disease. Nat Commun. 2018 Oct 25;9(1):4447. doi: 10.1038/s41467-018-06964-x.

Arnadottir GA, Oddsson A, Jensson BO, Gisladottir S, Simon MT, Arnthorsson AO, Katrinardottir H, Fridriksdottir R, Ivarsdottir EV, Jonasdottir A, Jonasdottir A, Barrick R, Saemundsdottir J, le Roux L, Oskarsson GR, Asmundsson J, Steffensen T, Gudmundsson KR, Ludvigsson P, Jonsson JJ, Masson G, Jonsdottir I, Holm H, Jonasson JG, Magnusson OT, Thorarensen O, Abdenur J, Norddahl GL, Gudbjartsson DF, Bjornsson HT, Thorsteinsdottir U, Sulem P, Stefansson K. Population-level deficit of homozygosity unveils CPSF3 as an intellectual disability syndrome gene. Nat Commun. 2022 Feb 4;13(1):705. doi: 10.1038/s41467-022-28330-8.

Arora H, Chacon AH, Choudhary S, McLeod MP, Meshkov L, Nouri K, Izakovic J. Bloom syndrome. Int J Dermatol. 2014 Jul;53(7):798-802. doi: 10.1111/ijd.12408. Epub 2014 Mar 6.

Auber B, Burfeind P, Herold S, Schoner K, Simson G, Rauskolb R, Rehder H. A disease causing deletion of 29 base pairs in intron 15 in the MKS1 gene is highly associated with the campomelic variant of the Meckel-Gruber syndrome. Clin Genet. 2007 Nov;72(5):454-9. doi: 10.1111/j.1399-0004.2007.00880.x.

Aula N, Salomäki P, Timonen R, Verheijen F, Mancini G, Månsson JE, Aula P, Peltonen L. The spectrum of SLC17A5-gene mutations resulting in free sialic acid-storage diseases indicates some genotype-phenotype correlation. Am J Hum Genet. 2000 Oct;67(4):832-40. doi: 10.1086/303077. Epub 2000 Aug 17.

Avela K, Lipsanen-Nyman M, Idänheimo N, Seemanová E, Rosengren S, Mäkelä TP, Perheentupa J, Chapelle AD, Lehesjoki AE. Gene encoding a new RING-B-box-Coiled-coil protein is mutated in mulibrey nanism. Nat Genet. 2000 Jul;25(3):298-301. doi: 10.1038/77053.

Avela K, Sankila EM, Seitsonen S, Kuuluvainen L, Barton S, Gillies S, Aittomäki K. A founder mutation in CERKL is a major cause of retinal dystrophy in Finland. Acta Ophthalmol. 2018 Mar;96(2):183-191. doi: 10.1111/aos.13551. Epub 2017 Oct 25.

Bandah D, Merin S, Ashhab M, Banin E, Sharon D. The spectrum of retinal diseases caused by NR2E3 mutations in Israeli and Palestinian patients. Arch Ophthalmol. 2009 Mar;127(3):297-302. doi: 10.1001/archophthalmol.2008.615.

Barca E, Ganetzky RD, Potluri P, Juanola-Falgarona M, Gai X, Li D, Jalas C, Hirsch Y, Emmanuele V, Tadesse S, Ziosi M, Akman HO, Chung WK, Tanji K, McCormick EM, Place E, Consugar M, Pierce EA, Hakonarson H, Wallace DC, Hirano M, Falk MJ. USMG5 Ashkenazi Jewish founder mutation impairs mitochondrial complex V dimerization and ATP synthesis. Hum Mol Genet. 2018 Oct 1;27(19):3305-3312. doi: 10.1093/hmg/ddy231.

Bargal R, Avidan N, Ben-Asher E, Olender Z, Zeigler M, Frumkin A, Raas-Rothschild A, Glusman G, Lancet D, Bach G. Identification of the gene causing mucolipidosis type IV. Nat Genet. 2000 Sep;26(1):118-23. doi: 10.1038/79095.

Behar DM, Inbar O, Shteinberg M, Gur M, Mussaffi H, Shoseyov D, Ashkenazi M, Alkrinawi S, Bormans C, Hakim F, Mei-Zahav M, Cohen-Cymberknoh M, Dagan A, Prais D, Sarouk I, Stafler P, Bar Aluma BE, Akler G, Picard E, Aviram M, Efrati O, Livnat G, Rivlin J, Bentur L, Blau H, Kerem E, Singer A. Nationwide genetic analysis for molecularly unresolved cystic fibrosis patients in a multiethnic society: implications for preconception carrier screening. Mol Genet Genomic Med. 2017 Feb 19;5(3):223-236. doi: 10.1002/mgg3.278. eCollection 2017 May.

Ben Brick AS, Laroussi N, Mesrati H, Kefi R, Bchetnia M, Lasram K, Ben Halim N, Romdhane L, Ouragini H, Marrakchi S, Boubaker MS, Meddeb Cherif M, Castiglia D, Hovnanian A, Abdelhak S, Turki H. Mutational founder effect in recessive dystrophic epidermolysis bullosa families from Southern Tunisia. Arch Dermatol Res. 2014 May;306(4):405-11. doi: 10.1007/s00403-013-1421-y. Epub 2013 Oct 30.

Ben-Farhat K, Ben-Mustapha I, Ben-Ali M, Rouault K, Hamami S, Mekki N, Ben-Chehida A, Larguèche B, Fitouri Z, Abdelmoula S, Khemiri M, Guediche MN, Boukthir S, Barsaoui S, Chemli J, Barbouche MR. A Founder Effect of c.257 + 2T > C Mutation in NCF2 Gene Underlies Severe Chronic Granulomatous Disease in Eleven Patients. J Clin Immunol. 2016 Aug;36(6):547-54. doi: 10.1007/s10875-016-0299-9. Epub 2016 May 25.

Ben Haj Ali A, Messaoud O, Elouej S, Talmoudi F, Ayed W, Mellouli F, Ouederni M, Hadiji S, De Sandre-Giovannoli A, Delague V, Lévy N, Bogliolo M, Surrallés J, Abdelhak S, Amouri A. FANCA Gene Mutations in North African Fanconi Anemia Patients. Front Genet. 2021 Feb 19;12:610050. doi: 10.3389/fgene.2021.610050. eCollection 2021.

Ben-Mustapha I, Ben-Ali M, Mekki N, Patin E, Harmant C, Bouguila J, Elloumi-Zghal H, Harbi A, Béjaoui M, Boughammoura L, Chemli J, Barbouche MR. A 1,100-year-old founder effect mutation in IL12B gene is responsible for Mendelian susceptibility to mycobacterial disease in Tunisian patients.Immunogenetics. 2014 Jan;66(1):67-71. doi: 10.1007/s00251-013-0739-0. Epub 2013 Oct 15.

Ben-Mustapha I, Ben-Farhat K, Guirat-Dhouib N, Dhemaied E, Larguèche B, Ben-Ali M, Chemli J, Bouguila J, Ben-Mansour L, Mellouli F, Khemiri M, Béjaoui M, Barbouche MR. Clinical, immunological and genetic findings of a large tunisian series of major histocompatibility complex class II deficiency patients. J Clin Immunol. 2013 May;33(4):865-70. doi: 10.1007/s10875-013-9863-8. Epub 2013 Jan 13.

Ben Rekaya M, Laroussi N, Messaoud O, Jones M, Jerbi M, Naouali C, Bouyacoub Y, Chargui M, Kefi R, Fazaa B, Boubaker MS, Boussen H, Mokni M, Abdelhak S, Zghal M, Khaled A, Yacoub-Youssef H. A founder large deletion mutation in Xeroderma pigmentosum-Variant form in Tunisia: implication for molecular diagnosis and therapy. Biomed Res Int. 2014;2014:256245. doi: 10.1155/2014/256245. Epub 2014 May 4.

Ben Saïd M, Hmani-Aifa M, Amar I, Baig SM, Mustapha M, Delmaghani S, Tlili A, Ghorbel A, Ayadi H, Van Camp G, Smith RJ, Tekin M, Masmoudi S. High frequency of the p.R34X mutation in the TMC1 gene associated with nonsyndromic hearing loss is due to founder effects. Genet Test Mol Biomarkers. 2010 Jun;14(3):307-11. doi: 10.1089/gtmb.2009.0174.

Ben-Yosef T, Ness SL, Madeo AC, Bar-Lev A, Wolfman JH, Ahmed ZM, Desnick RJ, Willner JP, Avraham KB, Ostrer H, Oddoux C, Griffith AJ, Friedman TB. A mutation of PCDH15 among Ashkenazi Jews with the type 1 Usher syndrome. N Engl J Med. 2003 Apr 24;348(17):1664-70. doi: 10.1056/NEJMoa021502.

Björnsson E, Thorgeirsson G, Helgadóttir A, Thorleifsson G, Sveinbjörnsson G, Kristmundsdóttir S, Jónsson H, Jónasdóttir A, Jónasdóttir Á, Sigurðsson Á, Guðnason T, Ólafsson Í, Sigurðsson EL, Sigurðardóttir Ó, Viðarsson B, Baldvinsson M, Bjarnason R, Danielsen R, Matthíasson SE, Thórarinsson BL, Grétarsdóttir S, Steinthórsdóttir V, Halldórsson BV, Andersen K, Arnar DO, Jónsdóttir I, Guðbjartsson DF, Hólm H, Thorsteinsdóttir U, Sulem P, Stefánsson K. Large-Scale Screening for Monogenic and Clinically Defined Familial Hypercholesterolemia in Iceland. Arterioscler Thromb Vasc Biol. 2021 Oct;41(10):2616-2628. doi: 10.1161/ATVBAHA.120.315904. Epub 2021 Aug 19.

Bjornsson T, Thorolfsdottir RB, Sveinbjornsson G, Sulem P, Norddahl GL, Helgadottir A, Gretarsdottir S, Magnusdottir A, Danielsen R, Sigurdsson EL, Adalsteinsdottir B, Gunnarsson SI, Jonsdottir I, Arnar DO, Helgason H, Gudbjartsson T, Gudbjartsson DF, Thorsteinsdottir U, Holm H, Stefansson K. A rare missense mutation in MYH6 associates with non-syndromic coarctation of the aorta. Eur Heart J. 2018 Sep 7;39(34):3243-3249. doi: 10.1093/eurheartj/ehy142.

Boualla L, Tajir M, Oulahiane N, Lyahyai J, Laarabi FZ, Chafai Elalaoui S, Soulami K, Ait Ouamar H, Sefiani A. AGXT Gene Mutations and Prevalence of Primary Hyperoxaluria Type 1 in Moroccan Population. Genet Test Mol Biomarkers. 2015 Nov;19(11):623-8. doi: 10.1089/gtmb.2015.0136. Epub 2015 Sep 18.

Bouzidi A, Charoute H, Charif M, Amalou G, Kandil M, Barakat A, Lenaers G. Clinical and genetic spectrums of 413 North African families with inherited retinal dystrophies and optic neuropathies. Orphanet J Rare Dis. 2022 May 12;17(1):197. doi: 10.1186/s13023-022-02340-7.

Boyle L, Wamelink MMC, Salomons GS, Roos B, Pop A, Dauber A, Hwa V, Andrew M, Douglas J, Feingold M, Kramer N, Saitta S, Retterer K, Cho MT, Begtrup A, Monaghan KG, Wynn J, Chung WK. Mutations in TKT Are the Cause of a Syndrome Including Short Stature, Developmental Delay, and Congenital Heart Defects. Am J Hum Genet. 2016 Jun 2;98(6):1235-1242. doi: 10.1016/j.ajhg.2016.03.030.

Bressman SB, Sabatti C, Raymond D, de Leon D, Klein C, Kramer PL, Brin MF, Fahn S, Breakefield X, Ozelius LJ, Risch NJ. The DYT1 phenotype and guidelines for diagnostic testing. Neurology. 2000 May 9;54(9):1746-52. doi: 10.1212/wnl.54.9.1746.

Brownstein Z, Gulsuner S, Walsh T, Martins FTA, Taiber S, Isakov O, Lee MK, Bordeynik-Cohen M, Birkan M, Chang W, Casadei S, Danial-Farran N, Abu-Rayyan A, Carlson R, Kamal L, Arnthórsson AÖ, Sokolov M, Gilony D, Lipschitz N, Frydman M, Davidov B, Macarov M, Sagi M, Vinkler C, Poran H, Sharony R, Samra N, Zvi N, Baris-Feldman H, Singer A, Handzel O, Hertzano R, Ali-Naffaa D, Ruhrman-Shahar N, Madgar O, Sofrin-Drucker E, Peleg A, Khayat M, Shohat M, Basel-Salmon L, Pras E, Lev D, Wolf M, Steingrimsson E, Shomron N, Kelley MW, Kanaan MN, Allon-Shalev S, King MC, Avraham KB. Spectrum of genes for inherited hearing loss in the Israeli Jewish population, including the novel human deafness gene ATOH1. Clin Genet. 2020 Oct;98(4):353-364. doi: 10.1111/cge.13817. Epub 2020 Aug 24.

Castellsagué E, Liu J, Volenik A, Giroux S, Gagné R, Maranda B, Roussel-Jobin A, Latreille J, Laframboise R, Palma L, Kasprzak L, Marcus VA, Breguet M, Nolet S, El-Haffaf Z, Australie K, Gologan A, Aleynikova O, Oros-Klein K, Greenwood C, Mes-Masson AM, Provencher D, Tischkowitz M, Chong G, Rousseau F, Foulkes WD. Characterization of a novel founder MSH6 mutation causing Lynch syndrome in the French Canadian population. Clin Genet. 2015 Jun;87(6):536-42. doi: 10.1111/cge.12526. Epub 2014 Nov 22.

Cavallone L, Arcand SL, Maugard CM, Nolet S, Gaboury LA, Mes-Masson AM, Ghadirian P, Provencher D, Tonin PN. Comprehensive BRCA1 and BRCA2 mutation analyses and review of French Canadian families with at least three cases of breast cancer. Fam Cancer. 2010 Dec;9(4):507-17. doi: 10.1007/s10689-010-9372-3.

Charfeddine C, Ktaifi C, Laroussi N, Hammami H, Jmel H, Landoulsi Z, Badri T, Benmously R, Bchetnia M, Boubaker MS, Fenniche S, Abdelhak S, Mokni M. Clinical and molecular investigation of Buschke-Fischer-Brauer in consanguineous Tunisian families. J Eur Acad Dermatol Venereol. 2016 Dec;30(12):2122-2130. doi: 10.1111/jdv.13787. Epub 2016 Jul 12.

Chkioua L, Khedhiri S, Ben Turkia H, Chahed H, Ferchichi S, Ben Dridi MF, Laradi S, Miled A. Hurler disease (mucopolysaccharidosis type IH): clinical features and consanguinity in Tunisian population. Diagn Pathol. 2011 Nov 10;6:113. doi: 10.1186/1746-1596-6-113.

Chung WK, Martin K, Jalas C, Braddock SR, Juusola J, Monaghan KG, Warner B, Franks S, Yudkoff M, Lulis L, Rhodes RH, Prasad V, Torti E, Cho MT, Shinawi M. Mutations in COQ4, an essential component of coenzyme Q biosynthesis, cause lethal neonatal mitochondrial encephalomyopathy. J Med Genet. 2015 Sep;52(9):627-35. doi: 10.1136/jmedgenet-2015-103140. Epub 2015 Jul 16.

Cruz Marino T, Tardif J, Leblanc J, Lavoie J, Morin P, Harvey M, Thomas MJ, Pratte A, Braverman N. First glance at the molecular etiology of hearing loss in French-Canadian families from Saguenay-Lac-Saint-Jean's founder population. Hum Genet. 2022 Apr;141(3-4):607-622. doi: 10.1007/s00439-021-02332-w. Epub 2021 Aug 13.

Cruz Marino T, Villeneuve H, Leblanc J, Duranceau C, Caron P, Morin C, Milot M, Chrétien R, Gagnon MM, Mathieu J, Ellezam B, Buhas D. French-Canadian families from Saguenay-Lac-Saint-Jean: a new founder population for APECED. Endocrine. 2022 Jan;75(1):48-58. doi: 10.1007/s12020-021-02826-7. Epub 2021 Nov 30.

Daas S, Abu Salah N, Anikster Y, Barel O, Damseh NS, Dumin E, Fattal-Valevski A, Falik-Zaccai TC, Habib C, Josefsberg S, Korman SH, Kneller K, Landau Y, Lerman-Sagie T, Mandel H, Manor Y, Moady Abdalla T, Rock R, Rostami N, Saada A, Saraf-Levy T, Shaul Lotan N, Spiegel R, Staretz-Chacham O, Tal G, Ulanovsky I, Vaisid T, Wilnai Y, Almashanu S. Addition of galactose-1-phosphate measurement enhances newborn screening for classical galactosemia. J Inherit Metab Dis. 2023 Mar;46(2):232-242. doi: 10.1002/jimd.12580. Epub 2022 Dec 20.

Daum H, Meiner V, Michaelson-Cohen R, Sukenik-Halevy R, Zalcberg ML, Bar-Ziv A, Weiden AT, Scher SY, Shohat M, Zlotogora J. Smith-Lemli-Opitz syndrome: what is the actual risk for couples carriers of the DHCR7:c.964-1G>C variant? Eur J Hum Genet. 2020 Jul;28(7):938-942. doi: 10.1038/s41431-020-0577-0. Epub 2020 Feb 13.

Debray FG, Lambert M, Lemieux B, Soucy JF, Drouin R, Fenyves D, Dubé J, Maranda B, Laframboise R, Mitchell GA. Phenotypic variability among patients with hyperornithinaemia-hyperammonaemia-homocitrullinuria syndrome homozygous for the delF188 mutation in SLC25A15. J Med Genet. 2008 Nov;45(11):759-64. doi: 10.1136/jmg.2008.059097.

Debray FG, Morin C, Janvier A, Villeneuve J, Maranda B, Laframboise R, Lacroix J, Decarie JC, Robitaille Y, Lambert M, Robinson BH, Mitchell GA. LRPPRC mutations cause a phenotypically distinct form of Leigh syndrome with cytochrome c oxidase deficiency. J Med Genet. 2011 Mar;48(3):183-9. doi: 10.1136/jmg.2010.081976. Epub 2011 Jan 25.

de la Chapelle A, Kere J, Sack GH Jr, Tolvanen R, Maury CP. Familial amyloidosis, Finnish type: G654----a mutation of the gelsolin gene in Finnish families and an unrelated American family. Genomics. 1992 Jul;13(3):898-901. doi: 10.1016/0888-7543(92)90182-r.

Diaz GA, Gelb BD, Risch N, Nygaard TG, Frisch A, Cohen IJ, Miranda CS, Amaral O, Maire I, Poenaru L, Caillaud C, Weizberg M, Mistry P, Desnick RJ. Gaucher disease: the origins of the Ashkenazi Jewish N370S and 84GG acid beta-glucosidase mutations. Am J Hum Genet. 2000 Jun;66(6):1821-32. doi: 10.1086/302946. Epub 2000 Apr 21.

Diesen C, Saarinen A, Pihko H, Rosenlew C, Cormand B, Dobyns WB, Dieguez J, Valanne L, Joensuu T, Lehesjoki AE. POMGnT1 mutation and phenotypic spectrum in muscle-eye-brain disease. J Med Genet. 2004 Oct;41(10):e115. doi: 10.1136/jmg.2004.020701.

Dieterich K, Soto Rifo R, Faure AK, Hennebicq S, Ben Amar B, Zahi M, Perrin J, Martinez D, Sèle B, Jouk PS, Ohlmann T, Rousseaux S, Lunardi J, Ray PF. Homozygous mutation of AURKC yields large-headed polyploid spermatozoa and causes male infertility. Nat Genet. 2007 May;39(5):661-5. doi: 10.1038/ng2027. Epub 2007 Apr 15.

Dobson-Stone C, Velayos-Baeza A, Jansen A, Andermann F, Dubeau F, Robert F, Summers A, Lang AE, Chouinard S, Danek A, Andermann E, Monaco AP. Identification of a VPS13A founder mutation in French Canadian families with chorea-acanthocytosis. Neurogenetics. 2005 Sep;6(3):151-8. doi: 10.1007/s10048-005-0220-9. Epub 2005 Sep 28.

Doherty E, Pakarinen P, Tiitinen A, Kiilavuori A, Huhtaniemi I, Forrest S, Aittomäki K. A Novel mutation in the FSH receptor inhibiting signal transduction and causing primary ovarian failure. J Clin Endocrinol Metab. 2002 Mar;87(3):1151-5. doi: 10.1210/jcem.87.3.8319.

Douglas SPM, Siipola P, Kovanen PE, Pyörälä M, Kakko S, Savolainen ER, Salmenniemi U, Orte K, Kytölä S, Pitkänen E, Porkka K, Kilpivaara O, Wartiovaara-Kautto U. ERCC6L2 defines a novel entity within inherited acute myeloid leukemia. Blood. 2019 Jun 20;133(25):2724-2728. doi: 10.1182/blood-2019-01-896233. Epub 2019 Apr 1.

Dupré N, Howard HC, Mathieu J, Karpati G, Vanasse M, Bouchard JP, Carpenter S, Rouleau GA. Hereditary motor and sensory neuropathy with agenesis of the corpus callosum. Ann Neurol. 2003 Jul;54(1):9-18. doi: 10.1002/ana.77777.

Durst R, Colombo R, Shpitzen S, Avi LB, Friedlander Y, Wexler R, Raal FJ, Marais DA, Defesche JC, Mandelshtam MY, Kotze MJ, Leitersdorf E, Meiner V. Recent origin and spread of a common Lithuanian mutation, G197del LDLR, causing familial hypercholesterolemia: positive selection is not always necessary to account for disease incidence among Ashkenazi Jews. Am J Hum Genet. 2001 May;68(5):1172-88. doi: 10.1086/320123. Epub 2001 Apr 17.

Ebermann I, Koenekoop RK, Lopez I, Bou-Khzam L, Pigeon R, Bolz HJ. An USH2A founder mutation is the major cause of Usher syndrome type 2 in Canadians of French origin and confirms common roots of Quebecois and Acadians. Eur J Hum Genet. 2009 Jan;17(1):80-4. doi: 10.1038/ejhg.2008.143. Epub 2008 Jul 30.

Edelmann L, Wasserstein MP, Kornreich R, Sansaricq C, Snyderman SE, Diaz GA. Maple syrup urine disease: identification and carrier-frequency determination of a novel founder mutation in the Ashkenazi Jewish population. Am J Hum Genet. 2001 Oct;69(4):863-8. doi: 10.1086/323677. Epub 2001 Aug 16.

Ekstein J, Rubin BY, Anderson SL, Weinstein DA, Bach G, Abeliovich D, Webb M, Risch N. Mutation frequencies for glycogen storage disease Ia in the Ashkenazi Jewish population. Am J Med Genet A. 2004 Aug 30;129A(2):162-4. doi: 10.1002/ajmg.a.30232.

Elhayek D, Perez de Nanclares G, Chouchane S, Hamami S, Mlika A, Troudi M, Leban N, Ben Romdane W, Gueddiche MN, El Amri F, Mrabet S, Ben Chibani J, Castaño L, Haj Khelil A, Ariceta G. Molecular diagnosis of distal renal tubular acidosis in Tunisian patients: proposed algorithm for Northern Africa populations for the ATP6V1B1, ATP6V0A4 and SCL4A1 genes. BMC Med Genet. 2013 Nov 20;14:119. doi: 10.1186/1471-2350-14-119.

El Kerch F, Ratbi I, Sbiti A, Laarabi FZ, Barkat A, Sefiani A. Carrier frequency of the c.525delT mutation in the SGCG gene and estimated prevalence of limb girdle muscular dystrophy type 2C among the Moroccan population. Genet Test Mol Biomarkers. 2014 Apr;18(4):253-6. doi: 10.1089/gtmb.2013.0326. Epub 2014 Feb 19.

Erkko H, Xia B, Nikkilä J, Schleutker J, Syrjäkoski K, Mannermaa A, Kallioniemi A, Pylkäs K, Karppinen SM, Rapakko K, Miron A, Sheng Q, Li G, Mattila H, Bell DW, Haber DA, Grip M, Reiman M, Jukkola-Vuorinen A, Mustonen A, Kere J, Aaltonen LA, Kosma VM, Kataja V, Soini Y, Drapkin RI, Livingston DM, Winqvist R. A recurrent mutation in PALB2 in Finnish cancer families. Nature. 2007 Mar 15;446(7133):316-9. doi: 10.1038/nature05609. Epub 2007 Feb 7.

Fedick AM, Shi L, Jalas C, Treff NR, Ekstein J, Kornreich R, Edelmann L, Mehta L, Savage SA. Carrier screening of RTEL1 mutations in the Ashkenazi Jewish population. Clin Genet. 2015 Aug;88(2):177-81. doi: 10.1111/cge.12459. Epub 2014 Sep 5.

Fierheller CT, Alenezi WM, Tonin PN. The Genetic Analyses of French Canadians of Quebec Facilitate the Characterization of New Cancer Predisposing Genes Implicated in Hereditary Breast and/or Ovarian Cancer Syndrome Families. Cancers (Basel). 2021 Jul 7;13(14):3406. doi: 10.3390/cancers13143406.

Fischer J, Bouadjar B, Heilig R, Huber M, Lefèvre C, Jobard F, Macari F, Bakija-Konsuo A, Ait-Belkacem F, Weissenbach J, Lathrop M, Hohl D, Prud'homme JF. Mutations in the gene encoding SLURP-1 in Mal de Meleda. Hum Mol Genet. 2001 Apr 1;10(8):875-80. doi: 10.1093/hmg/10.8.875.

Fossdal R, Jonasson F, Kristjansdottir GT, Kong A, Stefansson H, Gosh S, Gulcher JR, Stefansson K. A novel TEAD1 mutation is the causative allele in Sveinsson's chorioretinal atrophy (helicoid peripapillary chorioretinal degeneration). Hum Mol Genet. 2004 May 1;13(9):975-81. doi: 10.1093/hmg/ddh106. Epub 2004 Mar 11.

Fridriksdottir R, Jonsson AJ, Jensson BO, Sverrisson KO, Arnadottir GA, Skarphedinsdottir SJ, Katrinardottir H, Snaebjornsdottir S, Jonsson H, Eiriksson O, Oskarsson GR, Oddsson A, Jonasdottir A, Jonasdottir A, Sigurdsson GH, Indridason EP, Sigurdsson SB, Bjornsdottir G, Saemundsdottir J, Magnusson OT, Bjornsson HT, Thorsteinsdottir U, Sigurdsson TS, Sulem P, Sigurdsson MI, Stefansson K. Sequence variants in malignant hyperthermia genes in Iceland: classification and actionable findings in a population database. Eur J Hum Genet. 2021 Dec;29(12):1819-1824. doi: 10.1038/s41431-021-00954-2. Epub 2021 Aug 31.

Frisch A, Colombo R, Michaelovsky E, Karpati M, Goldman B, Peleg L. Origin and spread of the 1278insTATC mutation causing Tay-Sachs disease in Ashkenazi Jews: genetic drift as a robust and parsimonious hypothesis. Hum Genet. 2004 Mar;114(4):366-76. doi: 10.1007/s00439-003-1072-8. Epub 2004 Jan 15.

Gavino C, Hamel N, Zeng JB, Legault C, Guiot MC, Chankowsky J, Lejtenyi D, Lemire M, Alarie I, Dufresne S, Boursiquot JN, McIntosh F, Langelier M, Behr MA, Sheppard DC, Foulkes WD, Vinh DC. Impaired RASGRF1/ERK-mediated GM-CSF response characterizes CARD9 deficiency in French-Canadians. J Allergy Clin Immunol. 2016 Apr;137(4):1178-1188.e7. doi: 10.1016/j.jaci.2015.09.016. Epub 2015 Oct 29. PMID: 26521038

Glaser B, Blech I, Krakinovsky Y, Ekstein J, Gillis D, Mazor-Aronovitch K, Landau H, Abeliovich D. ABCC8 mutation allele frequency in the Ashkenazi Jewish population and risk of focal hyperinsulinemic hypoglycemia. Genet Med. 2011 Oct;13(10):891-4. doi: 10.1097/GIM.0b013e31821fea33.

Goldstein O, Nayshool O, Nefussy B, Traynor BJ, Renton AE, Gana-Weisz M, Drory VE, Orr-Urtreger A. OPTN 691_692insAG is a founder mutation causing recessive ALS and increased risk in heterozygotes. Neurology. 2016 Feb 2;86(5):446-53. doi: 10.1212/WNL.0000000000002334. Epub 2016 Jan 6.

Goldstein AM, Stacey SN, Olafsson JH, Jonsson GF, Helgason A, Sulem P, Sigurgeirsson B, Benediktsdottir KR, Thorisdottir K, Ragnarsson R, Kjartansson J, Kostic J, Masson G, Kristjansson K, Gulcher JR, Kong A, Thorsteinsdottir U, Rafnar T, Tucker MA, Stefansson K. CDKN2A mutations and melanoma risk in the Icelandic population. J Med Genet. 2008 May;45(5):284-9. doi: 10.1136/jmg.2007.055376. Epub 2008 Jan 4.

Gudbjartsson DF, Helgason H, Gudjonsson SA, Zink F, Oddson A, Gylfason A, Besenbacher S, Magnusson G, Halldorsson BV, Hjartarson E, Sigurdsson GT, Stacey SN, Frigge ML, Holm H, Saemundsdottir J, Helgadottir HT, Johannsdottir H, Sigfusson G, Thorgeirsson G, Sverrisson JT, Gretarsdottir S, Walters GB, Rafnar T, Thjodleifsson B, Bjornsson ES, Olafsson S, Thorarinsdottir H, Steingrimsdottir T, Gudmundsdottir TS, Theodors A, Jonasson JG, Sigurdsson A, Bjornsdottir G, Jonsson JJ, Thorarensen O, Ludvigsson P, Gudbjartsson H, Eyjolfsson GI, Sigurdardottir O, Olafsson I, Arnar DO, Magnusson OT, Kong A, Masson G, Thorsteinsdottir U, Helgason A, Sulem P, Stefansson K. Large-scale whole-genome sequencing of the Icelandic population. Nat Genet. 2015 May;47(5):435-44. doi: 10.1038/ng.3247. Epub 2015 Mar 25.

Guldberg P, Zschocke J, Dagbjartsson A, Henriksen KF, Güttler F. A molecular survey of phenylketonuria in Iceland: identification of a founding mutation and evidence of predominant Norse settlement. Eur J Hum Genet. 1997 Nov-Dec;5(6):376-81.

Hackman P, Vihola A, Haravuori H, Marchand S, Sarparanta J, De Seze J, Labeit S, Witt C, Peltonen L, Richard I, Udd B. Tibial muscular dystrophy is a titinopathy caused by mutations in TTN, the gene encoding the giant skeletal-muscle protein titin. Am J Hum Genet. 2002 Sep;71(3):492-500. doi: 10.1086/342380. Epub 2002 Jul 26.

Hamza W, Ali Pacha L, Hamadouche T, Muller J, Drouot N, Ferrat F, Makri S, Chaouch M, Tazir M, Koenig M, Benhassine T. Molecular and clinical study of a cohort of 110 Algerian patients with autosomal recessive ataxia. BMC Med Genet. 2015 Jun 12;16:36. doi: 10.1186/s12881-015-0180-3.

Hannah-Shmouni F, Morissette R, Sinaii N, Elman M, Prezant TR, Chen W, Pulver A, Merke DP. Revisiting the prevalence of nonclassic congenital adrenal hyperplasia in US Ashkenazi Jews and Caucasians. Genet Med. 2017 Nov;19(11):1276-1279. doi: 10.1038/gim.2017.46. Epub 2017 May 25.

Haraldsdottir S, Rafnar T, Frankel WL, Einarsdottir S, Sigurdsson A, Hampel H, Snaebjornsson P, Masson G, Weng D, Arngrimsson R, Kehr B, Yilmaz A, Haraldsson S, Sulem P, Stefansson T, Shields PG, Sigurdsson F, Bekaii-Saab T, Moller PH, Steinarsdottir M, Alexiusdottir K, Hitchins M, Pritchard CC, de la Chapelle A, Jonasson JG, Goldberg RM, Stefansson K. Comprehensive population-wide analysis of Lynch syndrome in Iceland reveals founder mutations in MSH6 and PMS2. Nat Commun. 2017 May 3;8:14755. doi: 10.1038/ncomms14755.

Hästbacka J, Kerrebrock A, Mokkala K, Clines G, Lovett M, Kaitila I, de la Chapelle A, Lander ES. Identification of the Finnish founder mutation for diastrophic dysplasia (DTD). Eur J Hum Genet. 1999 Sep;7(6):664-70. doi: 10.1038/sj.ejhg.5200361.

Heimer G, Kerätär JM, Riley LG, Balasubramaniam S, Eyal E, Pietikäinen LP, Hiltunen JK, Marek-Yagel D, Hamada J, Gregory A, Rogers C, Hogarth P, Nance MA, Shalva N, Veber A, Tzadok M, Nissenkorn A, Tonduti D, Renaldo F; University of Washington Center for Mendelian Genomics; Kraoua I, Panteghini C, Valletta L, Garavaglia B, Cowley MJ, Gayevskiy V, Roscioli T, Silberstein JM, Hoffmann C, Raas-Rothschild A, Tiranti V, Anikster Y, Christodoulou J, Kastaniotis AJ, Ben-Zeev B, Hayflick SJ. MECR Mutations Cause Childhood-Onset Dystonia and Optic Atrophy, a Mitochondrial Fatty Acid Synthesis Disorder. Am J Hum Genet. 2016 Dec 1;99(6):1229-1244. doi: 10.1016/j.ajhg.2016.09.021. Epub 2016 Nov 3.

Heimer G, Marek-Yagel D, Eyal E, Barel O, Oz Levi D, Hoffmann C, Ruzzo EK, Ganelin-Cohen E, Lancet D, Pras E, Rechavi G, Nissenkorn A, Anikster Y, Goldstein DB, Ben Zeev B. SLC1A4 mutations cause a novel disorder of intellectual disability, progressive microcephaly, spasticity and thin corpus callosum.Clin Genet. 2015 Oct;88(4):327-35. doi: 10.1111/cge.12637. Epub 2015 Jul 28.

Helman G, Zerem A, Almad A, Hacker JL, Woidill S, Sase S, LeFevre AN, Ekstein J, Johansson MM, Stutterd CA, Taft RJ, Simons C, Grinspan JB, Pizzino A, Schmidt JL, Harding B, Hirsch Y, Viaene AN, Fattal-Valevski A, Vanderver A. Further Delineation of the Clinical and Pathologic Features of HIKESHI-Related Hypomyelinating Leukodystrophy. Pediatr Neurol. 2021 Aug;121:11-19. doi: 10.1016/j.pediatrneurol.2021.04.014. Epub 2021 May 14.

Huopaniemi L, Rantala A, Forsius H, Somer M, de la Chapelle A, Alitalo T. Three widespread founder mutations contribute to high incidence of X-linked juvenile retinoschisis in Finland. Eur J Hum Genet. 1999 Apr;7(3):368-76. doi: 10.1038/sj.ejhg.5200300.

Jacobson SG, Aleman TS, Cideciyan AV, Sumaroka A, Schwartz SB, Windsor EA, Swider M, Herrera W, Stone EM. Leber congenital amaurosis caused by Lebercilin (LCA5) mutation: retained photoreceptors adjacent to retinal disorganization. Mol Vis. 2009 Jun 2;15:1098-106.

Jalas C, Anderson SL, Laufer T, Martimucci K, Bulanov A, Xie X, Ekstein J, Rubin BY. A founder mutation in the MPL gene causes congenital amegakaryocytic thrombocytopenia (CAMT) in the Ashkenazi Jewish population. Blood Cells Mol Dis. 2011 Jun 15;47(1):79-83. doi: 10.1016/j.bcmd.2011.03.006. Epub 2011 Apr 13.

Jaron R, Rosenfeld N, Zahdeh F, Carmi S, Beni-Adani L, Doviner V, Picard E, Segel R, Zeligson S, Carmel L, Renbaum P, Levy-Lahad E. Expanding the phenotype of CRB2 mutations - A new ciliopathy syndrome? Clin Genet. 2016 Dec;90(6):540-544. doi: 10.1111/cge.12764. Epub 2016 May 2.

Joensuu T, Hämäläinen R, Yuan B, Johnson C, Tegelberg S, Gasparini P, Zelante L, Pirvola U, Pakarinen L, Lehesjoki AE, de la Chapelle A, Sankila EM. Mutations in a novel gene with transmembrane domains underlie Usher syndrome type 3. Am J Hum Genet. 2001 Oct;69(4):673-84. doi: 10.1086/323610. Epub 2001 Aug 27.]

Kaul R, Gao GP, Balamurugan K, Matalon R. Cloning of the human aspartoacylase cDNA and a common missense mutation in Canavan disease. Nat Genet. 1993 Oct;5(2):118-23. doi: 10.1038/ng1093-118.

Kestilä M, Lenkkeri U, Männikkö M, Lamerdin J, McCready P, Putaala H, Ruotsalainen V, Morita T, Nissinen M, Herva R, Kashtan CE, Peltonen L, Holmberg C, Olsen A, Tryggvason K. Positionally cloned gene for a novel glomerular protein--nephrin--is mutated in congenital nephrotic syndrome. Mol Cell. 1998 Mar;1(4):575-82. doi: 10.1016/s1097-2765(00)80057-x.

Khachnaoui-Zaafrane K, Ouertani I, Zanati A, Kandara H, Maazoul F, Mrad R. 3M syndrome: A Tunisian seven-cases series. Eur J Med Genet. 2022 Mar;65(3):104448. doi: 10.1016/j.ejmg.2022.104448. Epub 2022 Feb 9.

Khattab A, Haider S, Kumar A, Dhawan S, Alam D, Romero R, Burns J, Li D, Estatico J, Rahi S, Fatima S, Alzahrani A, Hafez M, Musa N, Razzghy Azar M, Khaloul N, Gribaa M, Saad A, Charfeddine IB, Bilharinho de Mendonça B, Belgorosky A, Dumic K, Dumic M, Aisenberg J, Kandemir N, Alikasifoglu A, Ozon A, Gonc N, Cheng T, Kuhnle-Krahl U, Cappa M, Holterhus PM, Nour MA, Pacaud D, Holtzman A, Li S, Zaidi M, Yuen T, New MI. Clinical, genetic, and structural basis of congenital adrenal hyperplasia due to 11β-hydroxylase deficiency. Proc Natl Acad Sci U S A. 2017 Mar 7;114(10):E1933-E1940. doi: 10.1073/pnas.1621082114. Epub 2017 Feb 22.

Kimchi A, Meiner V, Silverstein S, Macarov M, Mor-Shaked H, Blumenfeld A, Audo I, Zeitz C, Mechoulam H, Banin E, Sharon D, Yahalom C. An Ashkenazi Jewish founder mutation in CACNA1F causes retinal phenotype in both hemizygous males and heterozygous female carriers. Ophthalmic Genet. 2019 Oct;40(5):443-448. doi: 10.1080/13816810.2019.1681008. Epub 2019 Oct 25.

Kolehmainen J, Black GC, Saarinen A, Chandler K, Clayton-Smith J, Träskelin AL, Perveen R, Kivitie-Kallio S, Norio R, Warburg M, Fryns JP, de la Chapelle A, Lehesjoki AE. Cohen syndrome is caused by mutations in a novel gene, COH1, encoding a transmembrane protein with a presumed role in vesicle-mediated sorting and intracellular protein transport. Am J Hum Genet. 2003 Jun;72(6):1359-69. doi: 10.1086/375454. Epub 2003 May 2.

Kuokkanen M, Kokkonen J, Enattah NS, Ylisaukko-Oja T, Komu H, Varilo T, Peltonen L, Savilahti E, Jarvela I. Mutations in the translated region of the lactase gene (LCT) underlie congenital lactase deficiency. Am J Hum Genet. 2006 Feb;78(2):339-44. doi: 10.1086/500053. Epub 2005 Dec 15.

Kure S, Takayanagi M, Narisawa K, Tada K, Leisti J. Identification of a common mutation in Finnish patients with nonketotic hyperglycinemia. J Clin Invest. 1992 Jul;90(1):160-4. doi: 10.1172/JCI115831.

Lahrouchi N, Postma AV, Salazar CM, De Laughter DM, Tjong F, Piherová L, Bowling FZ, Zimmerman D, Lodder EM, Ta-Shma A, Perles Z, Beekman L, Ilgun A, Gunst Q, Hababa M, Škorić-Milosavljević D, Stránecký V, Tomek V, de Knijff P, de Leeuw R, Robinson JY, Burn SC, Mustafa H, Ambrose M, Moss T, Jacober J, Niyazov DM, Wolf B, Kim KH, Cherny S, Rousounides A, Aristidou-Kallika A, Tanteles G, Ange-Line B, Denommé-Pichon AS, Francannet C, Ortiz D, Haak MC, Ten Harkel AD, Manten GT, Dutman AC, Bouman K, Magliozzi M, Radio FC, Santen GW, Herkert JC, Brown HA, Elpeleg O, van den Hoff MJ, Mulder B, Airola MV, Kmoch S, Barnett JV, Clur SA, Frohman MA, Bezzina CR. Biallelic loss-of-function variants in PLD1 cause congenital right-sided cardiac valve defects and neonatal cardiomyopathy.J Clin Invest. 2021 Mar 1;131(5):e142148. doi: 10.1172/JCI142148.

Larusso J, Ringpfeil F, Uitto J. Pseudoxanthoma elasticum: a streamlined, ethnicity-based mutation detection strategy. Clin Transl Sci. 2010 Dec;3(6):295-8. doi: 10.1111/j.1752-8062.2010.00243.x.

Levesque S, Morin C, Guay SP, Villeneuve J, Marquis P, Yik WY, Jiralerspong S, Bouchard L, Steinberg S, Hacia JG, Dewar K, Braverman NE. A founder mutation in the PEX6 gene is responsible for increased incidence of Zellweger syndrome in a French Canadian population. BMC Med Genet. 2012 Aug 15;13:72. doi: 10.1186/1471-2350-13-72.

Liebman SW, Palaganas H, Kobany H. A founder mutation in FLNC is likely a major cause of idiopathic dilated cardiomyopathy in Ashkenazi Jews. Int J Cardiol. 2021 Jan 15;323:124. doi: 10.1016/j.ijcard.2020.08.052. Epub 2020 Aug 15.

Lim ET, Würtz P, Havulinna AS, Palta P, Tukiainen T, Rehnström K, Esko T, Mägi R, Inouye M, Lappalainen T, Chan Y, Salem RM, Lek M, Flannick J, Sim X, Manning A, Ladenvall C, Bumpstead S, Hämäläinen E, Aalto K, Maksimow M, Salmi M, Blankenberg S, Ardissino D, Shah S, Horne B, McPherson R, Hovingh GK, Reilly MP, Watkins H, Goel A, Farrall M, Girelli D, Reiner AP, Stitziel NO, Kathiresan S, Gabriel S, Barrett JC, Lehtimäki T, Laakso M, Groop L, Kaprio J, Perola M, McCarthy MI, Boehnke M, Altshuler DM, Lindgren CM, Hirschhorn JN, Metspalu A, Freimer NB, Zeller T, Jalkanen S, Koskinen S, Raitakari O, Durbin R, MacArthur DG, Salomaa V, Ripatti S, Daly MJ, Palotie A; Sequencing Initiative Suomi (SISu) Project. Distribution and medical impact of loss-of-function variants in the Finnish founder population. PLoS Genet. 2014 Jul 31;10(7):e1004494. doi: 10.1371/journal.pgen.1004494. eCollection 2014 Jul.

Loeuillet C, Dhellemmes M, Cazin C, Kherraf ZE, Fourati Ben Mustapha S, Zouari R, Thierry-Mieg N, Arnoult C, Ray PF. A recurrent ZP1 variant is responsible for oocyte maturation defect with degenerated oocytes in infertile females. Clin Genet. 2022 Jul;102(1):22-29. doi: 10.1111/cge.14144. Epub 2022 Jun 1.

Lossos A, Dobson-Stone C, Monaco AP, Soffer D, Rahamim E, Newman JP, Mohiddin S, Fananapazir L, Lerer I, Linetsky E, Reches A, Argov Z, Abramsky O, Gadoth N, Sadeh M, Gomori JM, Boher M, Meiner V. Early clinical heterogeneity in choreoacanthocytosis. Arch Neurol. 2005 Apr;62(4):611-4. doi: 10.1001/archneur.62.4.611.

Ludviksson BR, Sigurdardottir ST, Johannsson JH, Haraldsson A, Hardarson TO. Epidemiology of Primary Immunodeficiency in Iceland. J Clin Immunol. 2015 Jan;35(1):75-9. doi: 10.1007/s10875-014-0107-3. Epub 2014 Oct 15.

Marino TC, Maranda B, Leblanc J, Pratte A, Barabas M, Dupéré A, Lévesque S. Novel founder mutation in French-Canadian families with Naxos disease. Clin Genet. 2017 Oct;92(4):451-453. doi: 10.1111/cge.12971. Epub 2017 Feb 22.

McGowan-Jordan J, Stoddard K, Podolsky L, Orrbine E, McLaine P, Town M, Goodyer P, MacKenzie A, Heick H. Molecular analysis of cystinosis: probable Irish origin of the most common French Canadian mutation. Eur J Hum Genet. 1999 Sep;7(6):671-8. doi: 10.1038/sj.ejhg.5200349.

Mee L, Honkala H, Kopra O, Vesa J, Finnilä S, Visapää I, Sang TK, Jackson GR, Salonen R, Kestilä M, Peltonen L. Hydrolethalus syndrome is caused by a missense mutation in a novel gene HYLS1. Hum Mol Genet. 2005 Jun 1;14(11):1475-88

Messaoud O, Ben Rekaya M, Cherif W, Talmoudi F, Boussen H, Mokhtar I, Boubaker S, Amouri A, Abdelhak S, Zghal M. Genetic homogeneity of mutational spectrum of group-A xeroderma pigmentosum in Tunisian patients. Int J Dermatol. 2010 May;49(5):544-8. doi: 10.1111/j.1365-4632.2010.04421.x.

Mili A, Ben Charfeddine I, Amara A, Mamaï O, Adala L, Ben Lazreg T, Bouguila J, Saad A, Limem K, Gribaa M. A c.3216_3217delGA mutation in AGL gene in Tunisian patients with a glycogen storage disease type III: evidence of a founder effect. Clin Genet. 2012 Dec;82(6):534-9. doi: 10.1111/j.1399-0004.2011.01806.x. Epub 2011 Nov 23

Mitchell GA, Brody LC, Sipila I, Looney JE, Wong C, Engelhardt JF, Patel AS, Steel G, Obie C, Kaiser-Kupfer M, et al. At least two mutant alleles of ornithine delta-aminotransferase cause gyrate atrophy of the choroid and retina in Finns. Proc Natl Acad Sci U S A. 1989 Jan;86(1):197-201. doi: 10.1073/pnas.86.1.197.

Mononen I, Heisterkamp N, Kaartinen V, Williams JC, Yates JR 3rd, Griffin PR, Hood LE, Groffen J. Aspartylglycosaminuria in the Finnish population: identification of two point mutations in the heavy chain of glycoasparaginase. Proc Natl Acad Sci U S A. 1991 Apr 1;88(7):2941-5. doi: 10.1073/pnas.88.7.2941.

Moulard B, Genton P, Grid D, Jeanpierre M, Ouazzani R, Mrabet A, Morris M, LeGuern E, Dravet C, Mauguière F, Utermann B, Baldy-Moulinier M, Belaidi H, Bertran F, Biraben A, Ali Chérif A, Chkili T, Crespel A, Darcel F, Dulac O, Geny C, Humbert-Claude V, Kassiotis P, Buresi C, Malafosse A. Haplotype study of West European and North African Unverricht-Lundborg chromosomes: evidence for a few founder mutations. Hum Genet. 2002 Sep;111(3):255-62. doi: 10.1007/s00439-002-0755-x. Epub 2002 Jul 23.

Ness SL, Ben-Yosef T, Bar-Lev A, Madeo AC, Brewer CC, Avraham KB, Kornreich R, Desnick RJ, Willner JP, Friedman TB, Griffith AJ. Genetic homogeneity and phenotypic variability among Ashkenazi Jews with Usher syndrome type III. J Med Genet. 2003 Oct;40(10):767-72. doi: 10.1136/jmg.40.10.767.

Neuser S, Brechmann B, Heimer G, Brösse I, Schubert S, O'Grady L, Zech M, Srivastava S, Sweetser DA, Dincer Y, Mall V, Winkelmann J, Behrends C, Darras BT, Graham RJ, Jayakar P, Byrne B, Bar-Aluma BE, Haberman Y, Szeinberg A, Aldhalaan HM, Hashem M, Al Tenaiji A, Ismayl O, Al Nuaimi AE, Maher K, Ibrahim S, Khan F, Houlden H, Ramakumaran VS, Pagnamenta AT, Posey JE, Lupski JR, Tan WH, ElGhazali G, Herman I, Muñoz T, Repetto GM, Seitz A, Krumbiegel M, Poli MC, Kini U, Efthymiou S, Meiler J, Maroofian R, Alkuraya FS, Abou Jamra R, Popp B, Ben-Zeev B, Ebrahimi-Fakhari D. Clinical, neuroimaging, and molecular spectrum of TECPR2-associated hereditary sensory and autonomic neuropathy with intellectual disability. Hum Mutat. 2021 Jun;42(6):762-776. doi: 10.1002/humu.24206. Epub 2021 May 11.

Nikali K, Suomalainen A, Saharinen J, Kuokkanen M, Spelbrink JN, Lönnqvist T, Peltonen L. Infantile onset spinocerebellar ataxia is caused by recessive mutations in mitochondrial proteins Twinkle and Twinky. Hum Mol Genet. 2005 Oct 15;14(20):2981-90.

Norio R. The Finnish Disease Heritage III: the individual diseases. Hum Genet. 2003 May;112(5-6):470-526. doi: 10.1007/s00439-002-0877-1. Epub 2003 Mar 8.

Nousiainen HO, Kestilä M, Pakkasjärvi N, Honkala H, Kuure S, Tallila J, Vuopala K, Ignatius J, Herva R, Peltonen L. Mutations in mRNA export mediator GLE1 result in a fetal motoneuron disease. Nat Genet. 2008 Feb;40(2):155-7. doi: 10.1038/ng.2007.65. Epub 2008 Jan 20.

Paloneva J, Kestilä M, Wu J, Salminen A, Böhling T, Ruotsalainen V, Hakola P, Bakker AB, Phillips JH, Pekkarinen P, Lanier LL, Timonen T, Peltonen L. Loss-of-function mutations in TYROBP (DAP12) result in a presenile dementia with bone cysts. Nat Genet. 2000 Jul;25(3):357-61. doi: 10.1038/77153.

Palsdottir A, Snorradottir AO, Thorsteinsson L. Hereditary cystatin C amyloid angiopathy: genetic, clinical, and pathological aspects. Brain Pathol. 2006 Jan;16(1):55-9. doi: 10.1111/j.1750-3639.2006.tb00561.x.

Palsson R, Jonasson JG, Kristjansson M, Bodvarsson A, Goldin RD, Cox DW, Olafsson S. Genotype-phenotype interactions in Wilson's disease: insight from an Icelandic mutation. Eur J Gastroenterol Hepatol. 2001 Apr;13(4):433-6. doi: 10.1097/00042737-200104000-00023.

Papadopoulos VP, Giaglis S, Mitroulis I, Ritis K. The population genetics of familial mediterranean fever: a meta-analysis study. Ann Hum Genet. 2008 Nov;72(Pt 6):752-61. doi: 10.1111/j.1469-1809.2008.00471.x. Epub 2008 Aug 6.

Pavlovsky M, Peled A, Samuelov L, Malki L, Malovitski K, Assaf S, Mohamad J, Meijers O, Eskin-Schwartz M, Sarig O, Sprecher E. Molecular epidemiology of pachyonychia congenita in the Israeli population. Clin Exp Dermatol. 2021 Jun;46(4):663-668. doi: 10.1111/ced.14509. Epub 2020 Dec 20.

Pellegata NS, Dieguez-Lucena JL, Joensuu T, Lau S, Montgomery KT, Krahe R, Kivelä T, Kucherlapati R, Forsius H, de la Chapelle A. Mutations in KERA, encoding keratocan, cause cornea plana. Nat Genet. 2000 May;25(1):91-5. doi: 10.1038/75664.

Pelttari LM, Kiiski J, Nurminen R, Kallioniemi A, Schleutker J, Gylfe A, Aaltonen LA, Leminen A, Heikkilä P, Blomqvist C, Bützow R, Aittomäki K, Nevanlinna H. A Finnish founder mutation in RAD51D: analysis in breast, ovarian, prostate, and colorectal cancer. J Med Genet. 2012 Jul;49(7):429-32. doi: 10.1136/jmedgenet-2012-100852. Epub 2012 May 31.

Pelttari LM, Shimelis H, Toiminen H, Kvist A, Törngren T, Borg Å, Blomqvist C, Bützow R, Couch F, Aittomäki K, Nevanlinna H. Gene-panel testing of breast and ovarian cancer patients identifies a recurrent RAD51C duplication. Clin Genet. 2018 Mar;93(3):595-602. doi: 10.1111/cge.13123. Epub 2018 Jan 12.

Peretz H, Mulai A, Usher S, Zivelin A, Segal A, Weisman Z, Mittelman M, Lupo H, Lanir N, Brenner B, Shpilberg O, Seligsohn U. The two common mutations causing factor XI deficiency in Jews stem from distinct founders: one of ancient Middle Eastern origin and another of more recent European origin. Blood. 1997 Oct 1;90(7):2654-9.

Plante M, Claveau S, Lepage P, Lavoie EM, Brunet S, Roquis D, Morin C, Vézina H, Laprise C. Mucolipidosis II: a single causal mutation in the N-acetylglucosamine-1-phosphotransferase gene (GNPTAB) in a French Canadian founder population. Clin Genet. 2008 Mar;73(3):236-44. doi: 10.1111/j.1399-0004.2007.00954.x. Epub 2008 Jan 7.

Polla DL, Rahikkala E, Bode MK, Määttä T, Varilo T, Loman T, Philips AK, Kurki M, Palotie A, Körkkö J, Vieira P, Avela K, Jacquemin V, Pirson I, Abramowicz M, de Brouwer APM, Kuismin O, van Bokhoven H, Järvelä I. Phenotypic spectrum associated with a CRADD founder variant underlying frontotemporal predominant pachygyria in the Finnish population. Eur J Hum Genet. 2019 Aug;27(8):1235-1243. doi: 10.1038/s41431-019-0383-8. Epub 2019 Mar 26.

Pras E, Raben N, Golomb E, Arber N, Aksentijevich I, Schapiro JM, Harel D, Katz G, Liberman U, Pras M, et al. Mutations in the SLC3A1 transporter gene in cystinuria. Am J Hum Genet. 1995 Jun;56(6):1297-303.

Quint A, Sagi M, Carmi S, Daum H, Macarov M, Ben Neriah Z, Meiner V, Elpeleg O, Lerer I. An Ashkenazi founder mutation in the PKHD1 gene. Eur J Med Genet. 2016 Feb;59(2):86-90. doi: 10.1016/j.ejmg.2015.12.013. Epub 2015 Dec 23.

Rabin R, Hirsch Y, Johansson MM, Ekstein J, Ekstein A, Pappas J. Severe epileptic encephalopathy associated with compound heterozygosity of THG1L variants in the Ashkenazi Jewish population. Am J Med Genet A. 2021 May;185(5):1589-1597. doi: 10.1002/ajmg.a.62147. Epub 2021 Mar 8.

Rafnar T, Gudbjartsson DF, Sulem P, Jonasdottir A, Sigurdsson A, Jonasdottir A, Besenbacher S, Lundin P, Stacey SN, Gudmundsson J, Magnusson OT, le Roux L, Orlygsdottir G, Helgadottir HT, Johannsdottir H, Gylfason A, Tryggvadottir L, Jonasson JG, de Juan A, Ortega E, Ramon-Cajal JM, García-Prats MD, Mayordomo C, Panadero A, Rivera F, Aben KK, van Altena AM, Massuger LF, Aavikko M, Kujala PM, Staff S, Aaltonen LA, Olafsdottir K, Bjornsson J, Kong A, Salvarsdottir A, Saemundsson H, Olafsson K, Benediktsdottir KR, Gulcher J, Masson G, Kiemeney LA, Mayordomo JI, Thorsteinsdottir U, Stefansson K. Mutations in BRIP1 confer high risk of ovarian cancer. Nat Genet. 2011 Oct 2;43(11):1104-7. doi: 10.1038/ng.955.

Rechavi E, Lev A, Simon AJ, Stauber T, Daas S, Saraf-Levy T, Broides A, Nahum A, Marcus N, Hanna S, Stepensky P, Toker O, Dalal I, Etzioni A, Almashanu S, Somech R. First Year of Israeli Newborn Screening for Severe Combined Immunodeficiency-Clinical Achievements and Insights. Front Immunol. 2017 Nov 6;8:1448. doi: 10.3389/fimmu.2017.01448. eCollection 2017.

Richard P, Gaudon K, Haddad H, Ammar AB, Genin E, Bauché S, Paturneau-Jouas M, Müller JS, Lochmüller H, Grid D, Hamri A, Nouioua S, Tazir M, Mayer M, Desnuelle C, Barois A, Chabrol B, Pouget J, Koenig J, Gouider-Khouja N, Hentati F, Eymard B, Hantaï D. The CHRNE 1293insG founder mutation is a frequent cause of congenital myasthenia in North Africa. Neurology. 2008 Dec 9;71(24):1967-72. doi: 10.1212/01.wnl.0000336921.51639.0b.

Ridanpää M, Sistonen P, Rockas S, Rimoin DL, Mäkitie O, Kaitila I. Worldwide mutation spectrum in cartilage-hair hypoplasia: ancient founder origin of the major70A-->G mutation of the untranslated RMRP. Eur J Hum Genet. 2002 Jul;10(7):439-47. doi: 10.1038/sj.ejhg.5200824.

Romdhane L, Kefi R, Azaiez H, Ben Halim N, Dellagi K, Abdelhak S. Founder mutations in Tunisia: implications for diagnosis in North Africa and Middle East. Orphanet J Rare Dis. 2012 Aug 21;7:52. doi: 10.1186/1750-1172-7-52.

Runolfsdottir HL, Sayer JA, Indridason OS, Edvardsson VO, Jensson BO, Arnadottir GA, Gudjonsson SA, Fridriksdottir R, Katrinardottir H, Gudbjartsson D, Thorsteinsdottir U, Sulem P, Stefansson K, Palsson R. Allele frequency of variants reported to cause adenine phosphoribosyltransferase deficiency. Eur J Hum Genet. 2021 Jul;29(7):1061-1070. doi: 10.1038/s41431-020-00805-6. Epub 2021 Mar 11.

Salovaara R, Loukola A, Kristo P, Kääriäinen H, Ahtola H, Eskelinen M, Härkönen N, Julkunen R, Kangas E, Ojala S, Tulikoura J, Valkamo E, Järvinen H, Mecklin JP, Aaltonen LA, de la Chapelle A. Population-based molecular detection of hereditary nonpolyposis colorectal cancer. J Clin Oncol. 2000 Jun;18(11):2193-200. doi: 10.1200/JCO.2000.18.11.2193.

Samuels ME, Majewski J, Alirezaie N, Fernandez I, Casals F, Patey N, Decaluwe H, Gosselin I, Haddad E, Hodgkinson A, Idaghdour Y, Marchand V, Michaud JL, Rodrigue MA, Desjardins S, Dubois S, Le Deist F, Awadalla P, Raymond V, Maranda B. Exome sequencing identifies mutations in the gene TTC7A in French-Canadian cases with hereditary multiple intestinal atresia. J Med Genet. 2013 May;50(5):324-9. doi: 10.1136/jmedgenet-2012-101483. Epub 2013 Feb 19.

Schuchman EH, Miranda SR. Niemann-Pick disease: mutation update, genotype/phenotype correlations, and prospects for genetic testing. Genet Test. 1997;1(1):13-9. doi: 10.1089/gte.1997.1.13.

Scriver CR. Human genetics: lessons from Quebec populations. Annu Rev Genomics Hum Genet. 2001;2:69-101. doi: 10.1146/annurev.genom.2.1.69.

Sharapova SO, Skomska-Pawliszak M, Rodina YA, Wolska-Kuśnierz B, Dabrowska-Leonik N, Mikołuć B, Pashchenko OE, Pasic S, Freiberger T, Milota T, Formánková R, Szaflarska A, Siedlar M, Avčin T, Markelj G, Ciznar P, Kalwak K, Kołtan S, Jackowska T, Drabko K, Gagro A, Pac M, Naumova E, Kandilarova S, Babol-Pokora K, Varabyou DS, Barendregt BH, Raykina EV, Varlamova TV, Pavlova AV, Grombirikova H, Debeljak M, Mersiyanova IV, Bondarenko AV, Chernyshova LI, Kostyuchenko LV, Guseva MN, Rascon J, Muleviciene A, Preiksaitiene E, Geier CB, Leiss-Piller A, Yamazaki Y, Kawai T, Walter JE, Kondratenko IV, Šedivá A, van der Burg M, Kuzmenko NB, Notarangelo LD, Bernatowska E, Aleinikova OV. The Clinical and Genetic Spectrum of 82 Patients With RAG Deficiency Including a c.256_257delAA Founder Variant in Slavic Countries.Front Immunol. 2020 Jun 10;11:900. doi: 10.3389/fimmu.2020.00900. eCollection 2020.

Sherman JB, Raben N, Nicastri C, Argov Z, Nakajima H, Adams EM, Eng CM, Cowan TM, Plotz PH. Common mutations in the phosphofructokinase-M gene in Ashkenazi Jewish patients with glycogenesis VII--and their population frequency. Am J Hum Genet. 1994 Aug;55(2):305-13.

Siitonen HA, Sotkasiira J, Biervliet M, Benmansour A, Capri Y, Cormier-Daire V, Crandall B, Hannula-Jouppi K, Hennekam R, Herzog D, Keymolen K, Lipsanen-Nyman M, Miny P, Plon SE, Riedl S, Sarkar A, Vargas FR, Verloes A, Wang LL, Kääriäinen H, Kestilä M. The mutation spectrum in RECQL4 diseases. Eur J Hum Genet. 2009 Feb;17(2):151-8. doi: 10.1038/ejhg.2008.154. Epub 2008 Aug 20.

Slaugenhaupt SA, Blumenfeld A, Gill SP, Leyne M, Mull J, Cuajungco MP, Liebert CB, Chadwick B, Idelson M, Reznik L, Robbins C, Makalowska I, Brownstein M, Krappmann D, Scheidereit C, Maayan C, Axelrod FB, Gusella JF. Tissue-specific expression of a splicing mutation in the IKBKAP gene causes familial dysautonomia. Am J Hum Genet. 2001 Mar;68(3):598-605. doi: 10.1086/318810. Epub 2001 Jan 22.

Slavotinek A, Kaylor J, Pierce H, Cahr M, DeWard SJ, Schneidman-Duhovny D, Alsadah A, Salem F, Schmajuk G, Mehta L. CRB2 mutations produce a phenotype resembling congenital nephrosis, Finnish type, with cerebral ventriculomegaly and raised alpha-fetoprotein. Am J Hum Genet. 2015 Jan 8;96(1):162-9. doi: 10.1016/j.ajhg.2014.11.013. Epub 2014 Dec 31.

Soufir N, Ged C, Bourillon A, Austerlitz F, Chemin C, Stary A, Armier J, Pham D, Khadir K, Roume J, Hadj-Rabia S, Bouadjar B, Taieb A, de Verneuil H, Benchiki H, Grandchamp B, Sarasin A. A prevalent mutation with founder effect in xeroderma pigmentosum group C from north Africa. J Invest Dermatol. 2010 Jun;130(6):1537-42. doi: 10.1038/jid.2009.409. Epub 2010 Jan 7.

Srour M, Schwartzentruber J, Hamdan FF, Ospina LH, Patry L, Labuda D, Massicotte C, Dobrzeniecka S, Capo-Chichi JM, Papillon-Cavanagh S, Samuels ME, Boycott KM, Shevell MI, Laframboise R, Désilets V; FORGE Canada Consortium, Maranda B, Rouleau GA, Majewski J, Michaud JL. Mutations in C5ORF42 cause Joubert syndrome in the French Canadian population. Am J Hum Genet. 2012 Apr 6;90(4):693-700. doi: 10.1016/j.ajhg.2012.02.011. Epub 2012 Mar 15.

Stacey SN, Sulem P, Johannsson OT, Helgason A, Gudmundsson J, Kostic JP, Kristjansson K, Jonsdottir T, Sigurdsson H, Hrafnkelsson J, Johannsson J, Sveinsson T, Myrdal G, Grimsson HN, Bergthorsson JT, Amundadottir LT, Gulcher JR, Thorsteinsdottir U, Kong A, Stefansson K. The BARD1 Cys557Ser variant and breast cancer risk in Iceland. PLoS Med. 2006 Jul;3(7):e217. doi: 10.1371/journal.pmed.0030217.

Stephen J, Vilboux T, Haberman Y, Pri-Chen H, Pode-Shakked B, Mazaheri S, Marek-Yagel D, Barel O, Di Segni A, Eyal E, Hout-Siloni G, Lahad A, Shalem T, Rechavi G, Malicdan MC, Weiss B, Gahl WA, Anikster Y. Congenital protein losing enteropathy: an inborn error of lipid metabolism due to DGAT1 mutations. Eur J Hum Genet. 2016 Aug;24(9):1268-73. doi: 10.1038/ejhg.2016.5. Epub 2016 Feb 17.

Straussberg R, Marom D, Sanado-Inbar E, Lakovsky Y, Horev G, Shalev SA, Lev D, Lerman-Sagie T, Leshinsky-Silver E. A possible genotype-phenotype correlation in Ashkenazi-Jewish individuals with Aicardi-Goutières syndrome associated with SAMHD1 mutation. J Child Neurol. 2015 Mar;30(4):490-5. doi: 10.1177/0883073814549241. Epub 2014 Sep 22.

Taggart RT, Smail D, Apolito C, Vladutiu GD. Novel mutations associated with carnitine palmitoyltransferase II deficiency. Hum Mutat. 1999;13(3):210-20. doi: 10.1002/(SICI)1098-1004(1999)13:3<210::AID-HUMU5>3.0.CO;2-0.

Tanner SM, Sturm AC, Baack EC, Liyanarachchi S, de la Chapelle A. Inherited cobalamin malabsorption. Mutations in three genes reveal functional and ethnic patterns. Orphanet J Rare Dis. 2012 Aug 28;7:56. doi: 10.1186/1750-1172-7-56.

Thorsteinsson DA, Stefansdottir V, Eysteinsson T, Thorisdottir S, Jonsson JJ. Molecular genetics of inherited retinal degenerations in Icelandic patients. Clin Genet. 2021 Aug;100(2):156-167. doi: 10.1111/cge.13967. Epub 2021 May 7.

Tipping AJ, Pearson T, Morgan NV, Gibson RA, Kuyt LP, Havenga C, Gluckman E, Joenje H, de Ravel T, Jansen S, Mathew CG. Molecular and genealogical evidence for a founder effect in Fanconi anemia families of the Afrikaner population of South Africa. Proc Natl Acad Sci U S A. 2001 May 8;98(10):5734-9. doi: 10.1073/pnas.091402398.

Torrents D, Mykkänen J, Pineda M, Feliubadaló L, Estévez R, de Cid R, Sanjurjo P, Zorzano A, Nunes V, Huoponen K, Reinikainen A, Simell O, Savontaus ML, Aula P, Palacín M. Identification of SLC7A7, encoding y+LAT-1, as the lysinuric protein intolerance gene. Nat Genet. 1999 Mar;21(3):293-6. doi: 10.1038/6809.

Trotta L, Hautala T, Hämäläinen S, Syrjänen J, Viskari H, Almusa H, Lepisto M, Kaustio M, Porkka K, Palotie A, Seppänen M, Saarela J. Enrichment of rare variants in population isolates: single AICDA mutation responsible for hyper-IgM syndrome type 2 in Finland. Eur J Hum Genet. 2016 Oct;24(10):1473-8. doi: 10.1038/ejhg.2016.37. Epub 2016 May 4.

Trotta L, Martelius T, Siitonen T, Hautala T, Hämäläinen S, Juntti H, Taskinen M, Ilander M, Andersson EI, Zavialov A, Kaustio M, Keski-Filppula R, Hershfield M, Mustjoki S, Tapiainen T, Seppänen M, Saarela J. ADA2 deficiency: Clonal lymphoproliferation in a subset of patients. J Allergy Clin Immunol. 2018 Apr;141(4):1534-1537.e8. doi: 10.1016/j.jaci.2018.01.012. Epub 2018 Jan 31. PMID: 29391253 No abstract available.

Tullio-Pelet A, Salomon R, Hadj-Rabia S, Mugnier C, de Laet MH, Chaouachi B, Bakiri F, Brottier P, Cattolico L, Penet C, Bégeot M, Naville D, Nicolino M, Chaussain JL, Weissenbach J, Munnich A, Lyonnet S. Mutant WD-repeat protein in triple-A syndrome. Nat Genet. 2000 Nov;26(3):332-5. doi: 10.1038/8164

Vanier MT, Ferlinz K, Rousson R, Duthel S, Louisot P, Sandhoff K, Suzuki K. Deletion of arginine (608) in acid sphingomyelinase is the prevalent mutation among Niemann-Pick disease type B patients from northern Africa. Hum Genet. 1993 Oct;92(4):325-30. doi: 10.1007/BF01247328.

Verlander PC, Kaporis A, Liu Q, Zhang Q, Seligsohn U, Auerbach AD. Carrier frequency of the IVS4 + 4 A-->T mutation of the Fanconi anemia gene FAC in the Ashkenazi Jewish population. Blood. 1995 Dec 1;86(11):4034-8.

Vesa J, Hellsten E, Verkruyse LA, Camp LA, Rapola J, Santavuori P, Hofmann SL, Peltonen L. Mutations in the palmitoyl protein thioesterase gene causing infantile neuronal ceroid lipofuscinosis. Nature. 1995 Aug 17;376(6541):584-7. doi: 10.1038/376584a0.

Vierimaa O, Georgitsi M, Lehtonen R, Vahteristo P, Kokko A, Raitila A, Tuppurainen K, Ebeling TM, Salmela PI, Paschke R, Gündogdu S, De Menis E, Mäkinen MJ, Launonen V, Karhu A, Aaltonen LA. Pituitary adenoma predisposition caused by germline mutations in the AIP gene. Science. 2006 May 26;312(5777):1228-30. doi: 10.1126/science.1126100.

Visapää I, Fellman V, Vesa J, Dasvarma A, Hutton JL, Kumar V, Payne GS, Makarow M, Van Coster R, Taylor RW, Turnbull DM, Suomalainen A, Peltonen L. GRACILE syndrome, a lethal metabolic disorder with iron overload, is caused by a point mutation in BCS1L. Am J Hum Genet. 2002 Oct;71(4):863-76. doi: 10.1086/342773. Epub 2002 Sep 5.

Wallace SE, Bean LJH. Resources for Genetics Professionals — Genetic Disorders Associated with Founder Variants Common in the Ashkenazi Jewish Population. 2018 Dec 13. In: Adam MP, Mirzaa GM, Pagon RA, et al., editors. GeneReviews® [Internet]. Seattle (WA): University of Washington, Seattle; 1993-2022. Available from: https://www.ncbi.nlm.nih.gov/sites/books/NBK535149/

Waters PJ, Lace B, Buhas D, Gravel S, Cyr D, Boucher RM, Bernard G, Lévesque S, Maranda B. HSD10 mitochondrial disease: p.Leu122Val variant, mild clinical phenotype, and founder effect in French-Canadian patients from Quebec. Mol Genet Genomic Med. 2019 Dec;7(12):e1000. doi: 10.1002/mgg3.1000. Epub 2019 Oct 26.

Wedenoja S, Pekansaari E, Höglund P, Mäkelä S, Holmberg C, Kere J. Update on SLC26A3 mutations in congenital chloride diarrhea. Hum Mutat. 2011 Jul;32(7):715-22. doi: 10.1002/humu.21498. Epub 2011 Jun 7.

Yanus GA, Savonevich EL, Sokolenko AP, Romanko AA, Ni VI, Bakaeva EK, Gorustovich OA, Bizin IV, Imyanitov EN. Founder vs. non-founder BRCA1/2 pathogenic alleles: the analysis of Belarusian breast and ovarian cancer patients and review of other studies on ethnically homogenous populations. Fam Cancer. 2022 May 21. doi: 10.1007/s10689-022-00296-y. Online ahead of print.

Yotova V, Labuda D, Zietkiewicz E, Gehl D, Lovell A, Lefebvre JF, Bourgeois S, Lemieux-Blanchard E, Labuda M, Vézina H, Houde L, Tremblay M, Toupance B, Heyer E, Hudson TJ, Laberge C. Anatomy of a founder effect: myotonic dystrophy in Northeastern Quebec. Hum Genet. 2005 Jul;117(2-3):177-87. doi: 10.1007/s00439-005-1298-8. Epub 2005 May 10.

Zhang J, Lachance V, Schaffner A, Li X, Fedick A, Kaye LE, Liao J, Rosenfeld J, Yachelevich N, Chu ML, Mitchell WG, Boles RG, Moran E, Tokita M, Gorman E, Bagley K, Zhang W, Xia F, Leduc M, Yang Y, Eng C, Wong LJ, Schiffmann R, Diaz GA, Kornreich R, Thummel R, Wasserstein M, Yue Z, Edelmann L. A Founder Mutation in VPS11 Causes an Autosomal Recessive Leukoencephalopathy Linked to Autophagic Defects. PLoS Genet. 2016 Apr 27;12(4):e1005848. doi: 10.1371/journal.pgen.1005848. eCollection 2016 Apr.

Zlotogora J. Forty-seven pathogenic variants causing autosomal recessive disorders are shared by Israeli and Saudi Arabian Arabs. Clin Genet. 2021 Jun;99(6):818-822. doi: 10.1111/cge.13938. Epub 2021 Feb 15.

Zlotogora J, Patrinos GP, Meiner V. Ashkenazi Jewish genomic variants: integrating data from the Israeli National Genetic Database and gnomAD. Genet Med. 2018 Aug;20(8):867-871. doi: 10.1038/gim.2017.193. Epub 2017 Nov 16.
